# Supplementary material for: Sensitivity towards HDAC inhibition is associated with RTK/MAPK pathway activation in gastric cancer
Source: EMBO Mol Med. 2022 Aug 22;14(10):e15705. doi: 10.15252/emmm.202215705 (PMC9549728; doi:10.15252/emmm.202215705)
Supplement: Supplementary file 1 — Expanded View Figures PDF [file EMMM-14-e15705-s002.pdf]

Expanded View Figures

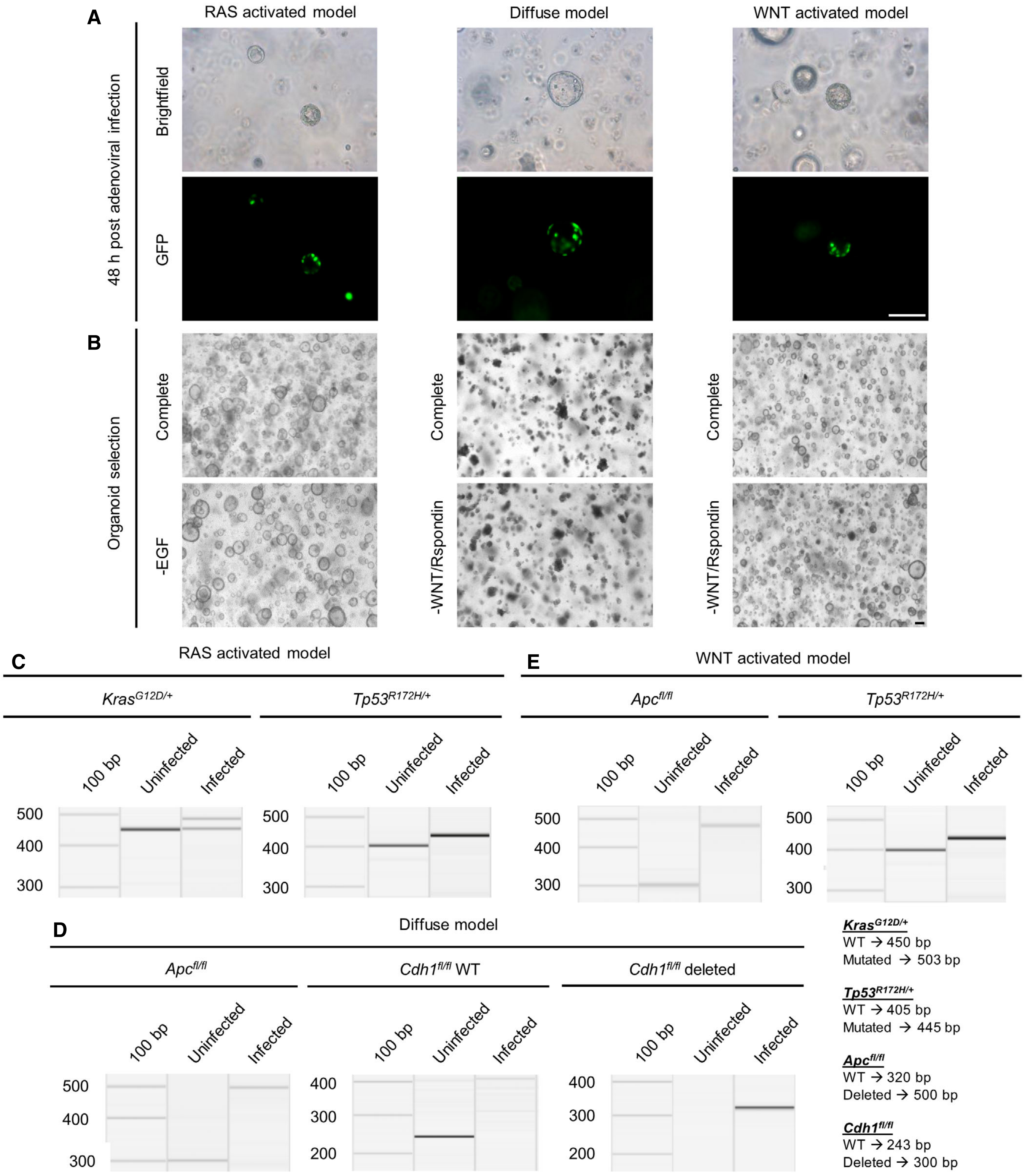

Figure EV1.

◀

Figure EV1. Generation of organoid models.

A Adenoviral infection of organoids with a Cre-GFP expressing recombinase. Fluorescence microscopy 24-h post-infection (scale bar 100 μm).

B Selection of organoids based on altered pathway. The RAS-activated model was selected via EGF removal from the normal cultivation medium. The diffuse and WNT-activated models were enriched by depletion of WNT3A and Rspondin (scale bar 25 μm).

C–E Genotyping PCRs of infected and selected organoid models to document successful recombination. *Tp53*$^{R172H/+}$ in the WNT-activated organoids showed loss of heterozygosity of the wild-type allele after activating the R172H mutation.

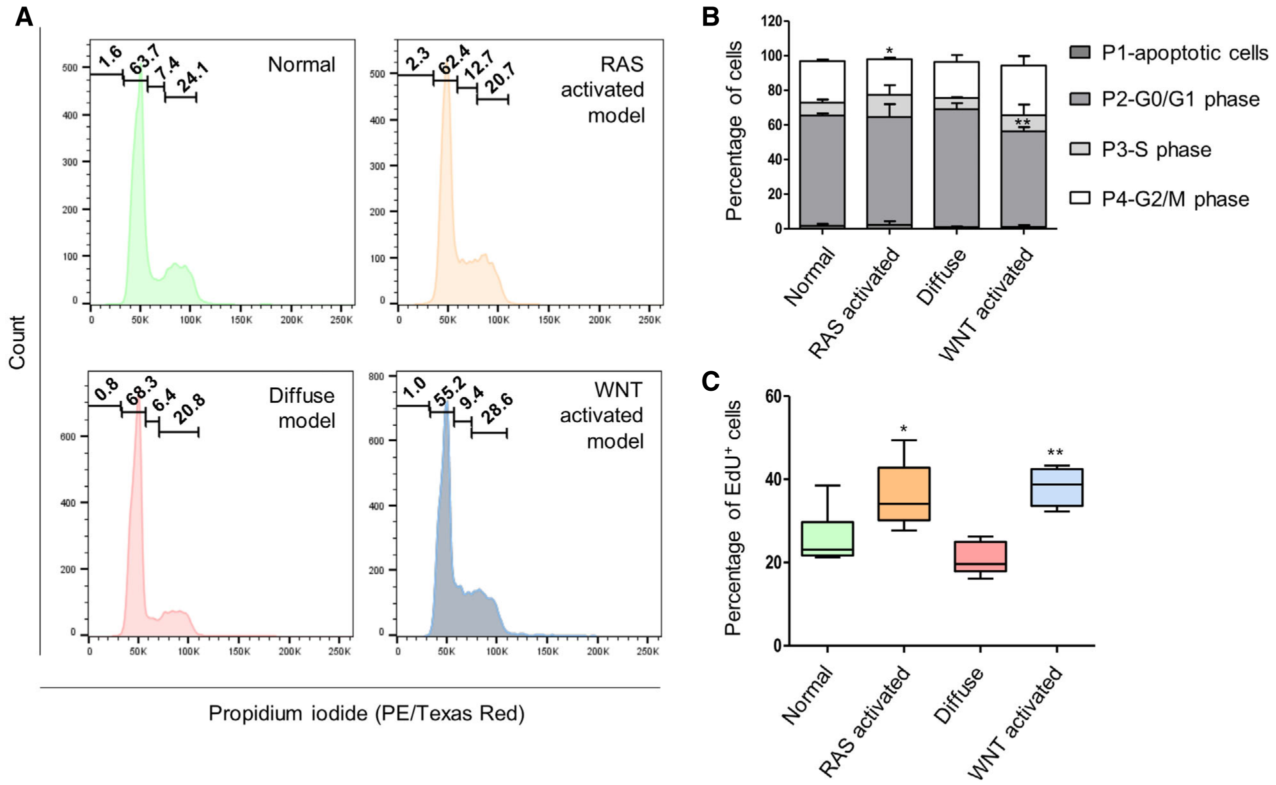

Figure EV2. Proliferative capacity of organoid models.

A Exemplary cell cycle analysis of normal organoids and organoid models.

B Quantitative representation of cell cycle analysis (two-tailed Student's *t*-test model vs. normal; *< 0.05; **< 0.01; RAS-activated P4 G2/M phase $P = 0.0105$, WNT-activated P2 G0/G1 phase $P = 0.0061$, biological replicate $n = 3$, data are shown as mean ± SD).

C Proliferation rate of organoids assessed by EdU proliferation assays. Two-tailed Student's *t*-test organoid models versus normal stomach organoids (*< 0.05; **< 0.01; RAS-activated $P = 0.031$; WNT-activated $P = 0.0017$, biological replicates $n = 3$, data are shown as mean ± SD).

 © 2022 The Authors

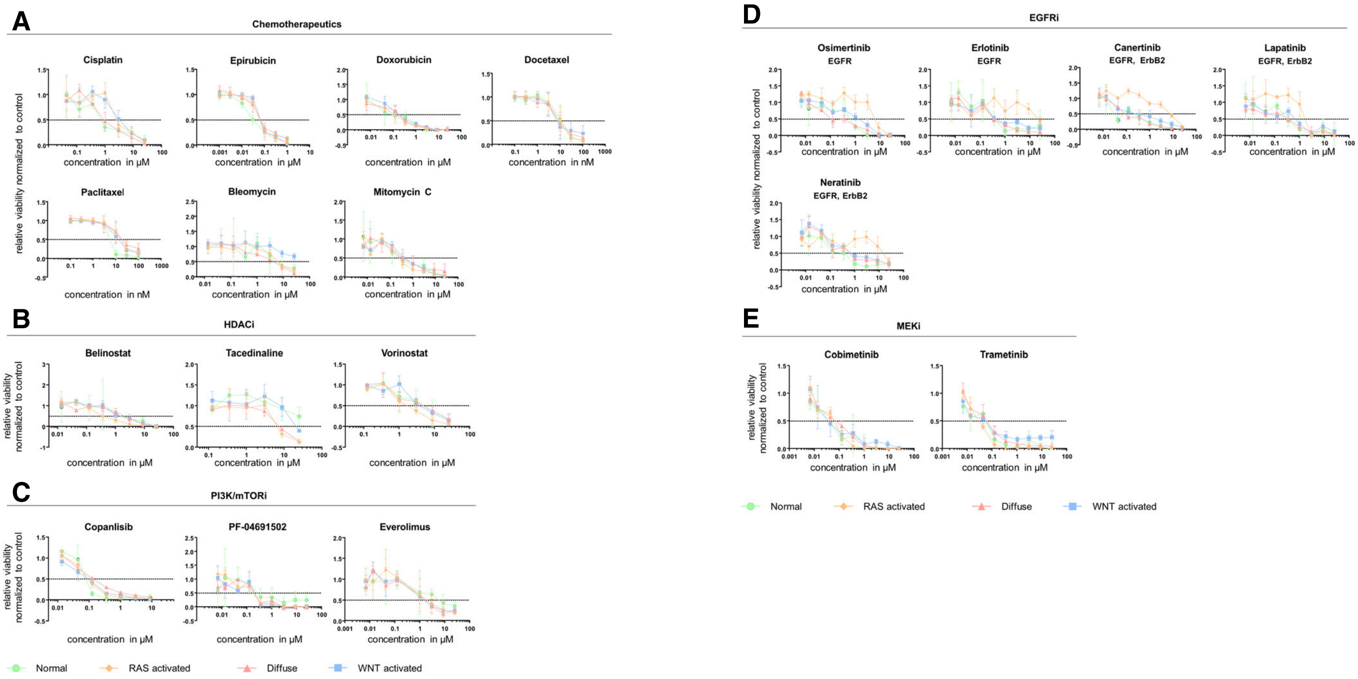

Figure EV3. Dose–response curves of normal and organoid models upon treatment with classical chemotherapeutics and targeted therapies.

A–E Drug response curves upon treatment with (A) classical chemotherapeutics (cisplatin, epirubicin, doxorubicin, docetaxel, paclitaxel, bleomycin, and mitomycin C), (B) HDAC inhibitors (belinostat, tacedenaline, and vorinostat), (C) PI3K/mTOR inhibitors (copanlisib, PF-04691502, and everolimus), (D) EGFR inhibitors (osimertinib, erlotinib, canertinib, lapatinib, and neratinib) and (E) MEK1/2 inhibitors (cobimetinib and trametinib) (biological replicates $n = 3$, data are shown as mean ± SD).

© 2022 The Authors

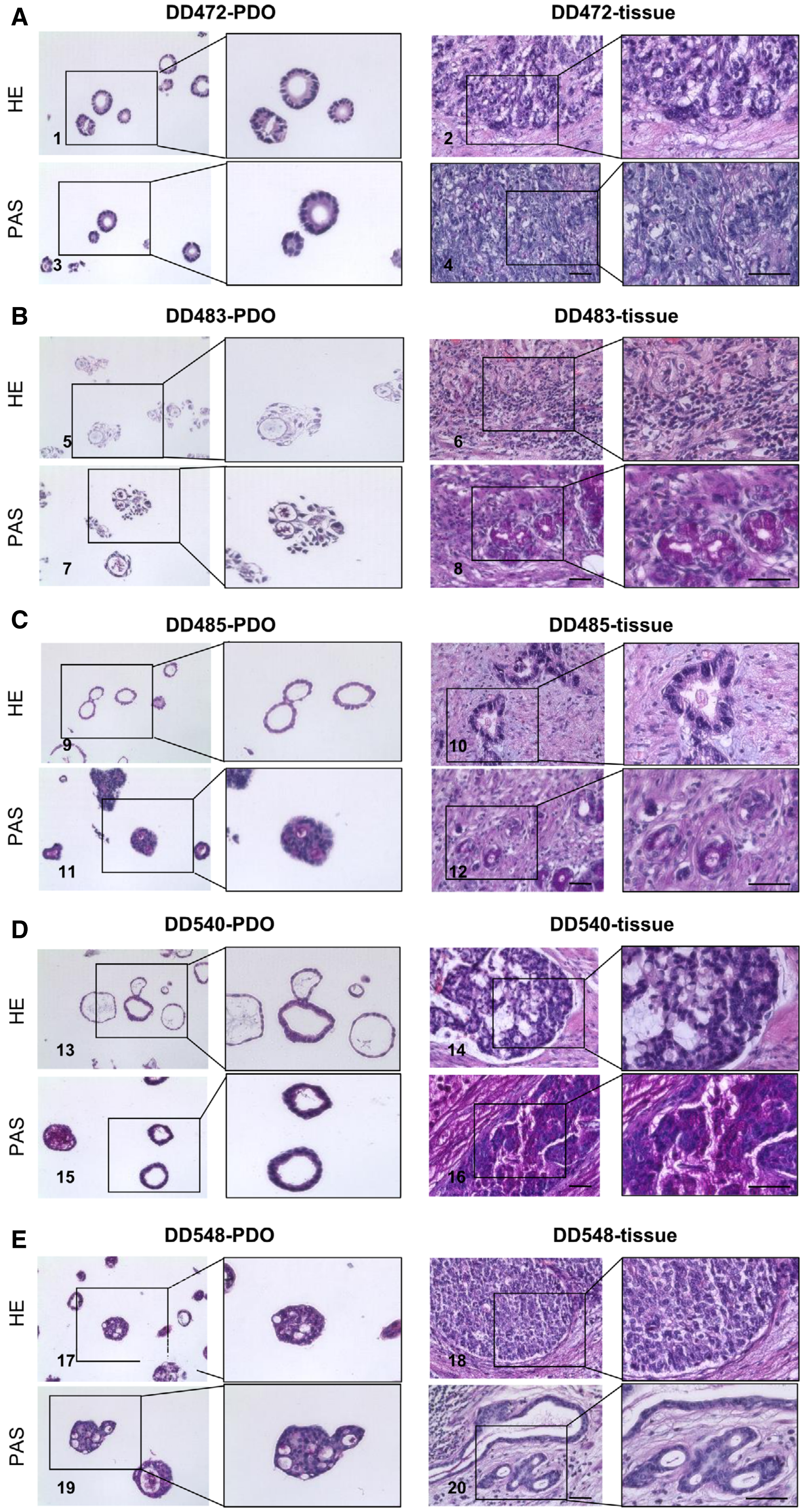

Figure EV4. Histological comparison of PDOs to corresponding primary tissue.

A HE staining (1 and 2) and PAS staining (3 and 4) of DD472 PDO and primary tissue (scale bar 50 μm).
B HE staining (5 and 6) and PAS staining (7 and 8) of DD483 PDO and primary tissue (scale bar 50 μm).
C HE staining (9 and 10) and PAS staining (11 and 12) of DD485 PDO and primary tissue (scale bar 50 μm).
D HE staining (13 and 14) and PAS staining (15 and 16) of DD540 PDO and primary tissue (scale bar 50 μm).
E HE staining (17 and 18) and PAS staining (19 and 20) of DD548 PDO and primary tissue (scale bar 50 μm).

© 2022 The Authors

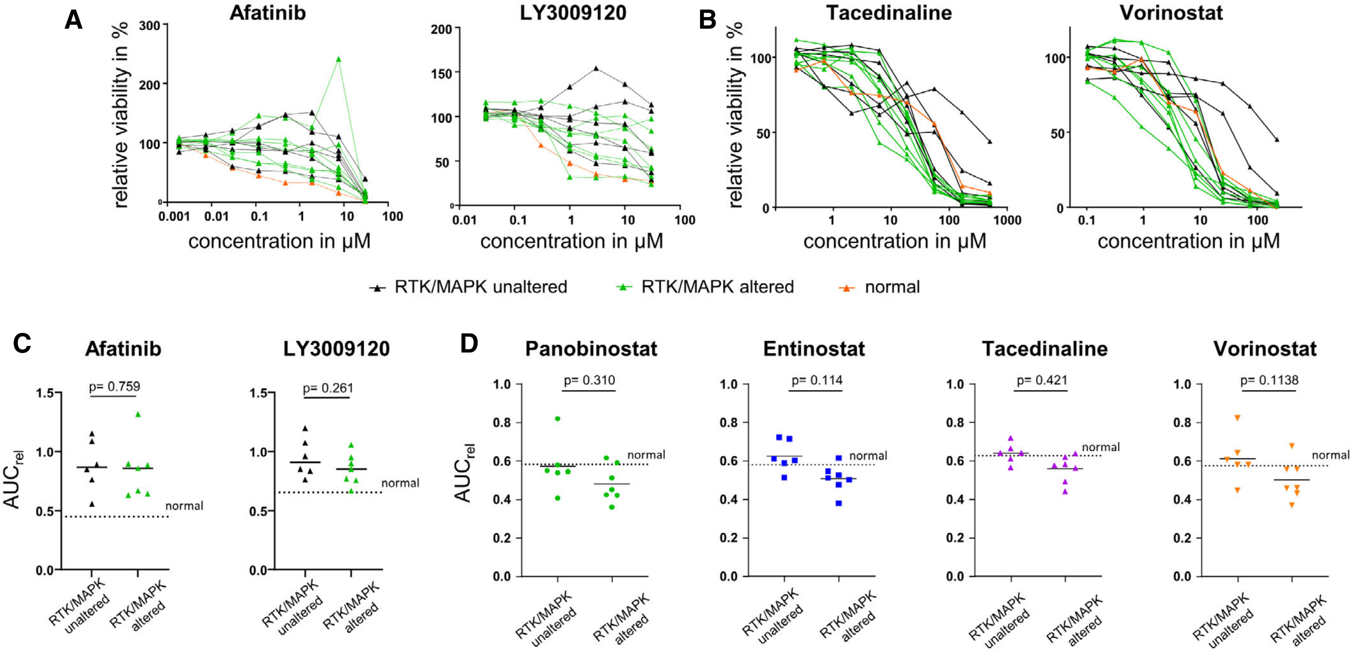

Figure EV5. EGFR, B-RAF, and HDAC inhibition of RTK/MAPK-altered versus non-altered gastric cancer PDOs.

A Drug response curves upon EGFR inhibition with afatinib or B-RAF with LY3009120 (biological replicates $n = 3$, data are presented as mean, SD values are shown in Table EV2).

B Drug response curves upon HDAC inhibition with tacedinaline and vorinostat (biological replicates $n = 3$, data are presented as mean, SD values are shown in Table EV2).

C Comparison of the relative area under the curve (AUC_{rel}) of afatinib and LY3009120-treated RTK/MAPK-altered (biological replicates $n = 7$) versus RTK/MAPK-unaltered human PDOs (biological replicates $n = 6$, two-tailed Student's t-test). The dashed line "normal" represents the AUC_{rel} of normal gastric PDOs as a reference parameter. (two-tailed Student's t-test).

D Comparison of the relative area under the curve (AUC_{rel}) of panobinostat, entinostat, tacedinaline, and vorinostat-treated RTK/MAPK-altered (biological replicates $n = 7$, two-tailed Student's t-test) versus RTK/MAPK-unaltered human PDOs (biological replicates $n = 6$). The dashed line "normal" represents the AUC_{rel} of normal gastric PDOs as a reference parameter.

© 2022 The Authors
