# Supplementary material for: Sensitivity towards HDAC inhibition is associated with RTK/MAPK pathway activation in gastric cancer
Source: EMBO Mol Med. 2022 Aug 22;14(10):e15705. doi: 10.15252/emmm.202215705 (PMC9549728; doi:10.15252/emmm.202215705)
Supplement: Supplementary file 8 — PDF+ [file EMMM-14-e15705-s008.pdf]

Article

TRANSPARENT PROCESS OPEN ACCESS

EMBO Molecular Medicine

Sensitivity towards HDAC inhibition is associated with RTK/MAPK pathway activation in gastric cancer

Therese Seidlitz[1,†], Tim Schmäche[1,2,3,4,†], Fernando García[5,†], Joon Ho Lee[1], Nan Qin[6], Susan Kochall[1], Juliane Fohgrub[1], David Pauck[6], Alexander Rothe[1], Bon-Kyoung Koo[7,8], Jürgen Weitz[1,2,3,4], Marc Remke[6], Javier Muñoz[5,9,10,*] & Daniel E Stange[1,2,3,4,**]

Abstract

Gastric cancer ranks the fifth most common and third leading cause of cancer-related deaths worldwide. Alterations in the RTK/MAPK, WNT, cell adhesion, TP53, TGFβ, NOTCH, and NFκB signaling pathways could be identified as main oncogenic drivers. A combination of altered pathways can be associated with molecular subtypes of gastric cancer. In order to generate model systems to study the impact of different pathway alterations in a defined genetic background, we generated three murine organoid models: a RAS-activated (*Kras*^{G12D}, *Tp53*^{R172H}), a WNT-activated (*Apc*$^{fl/fl}$, *Tp53*^{R172H}), and a diffuse (*Cdh1*$^{fl/fl}$, *Apc*$^{fl/fl}$) model. These organoid models were morphologically and phenotypically diverse, differed in proteome expression signatures and possessed individual drug sensitivities. A differential vulnerability to RTK/MAPK pathway interference based on the different mitogenic drivers and according to the level of dependence on the pathway could be uncovered. Furthermore, an association between RTK/MAPK pathway activity and susceptibility to HDAC inhibition was observed. This finding was further validated in patient-derived organoids from gastric adenocarcinoma, thus identifying a novel treatment approach for RTK/MAPK pathway altered gastric cancer patients.

Keywords gastric cancer; HDACi; MAPK; organoids
Subject Categories Cancer; Signal Transduction
DOI 10.15252/emmm.202215705 | Received 19 January 2022 | Revised 1 August 2022 | Accepted 2 August 2022 | Published online 22 August 2022
EMBO Mol Med (2022) 14: e15705

Introduction

Gastric cancer ranks as the fifth most common and third leading cause of cancer-related deaths worldwide (Bray *et al*, 2018; Ferlay *et al*, 2019). The diagnosis of gastric cancer is often delayed due to the lack of early clinical signs resulting in a high percentage of patients with incurable disease (Hunt *et al*, 2015). The widely used Lauren classification divided gastric cancer based on the morphological appearance into the intestinal, diffuse, and intermediate types (Lauren, 1965). Next to the histology-based classification, several attempts have been made to use molecular data to classify gastric cancer. The Asian Cancer Research Group (ACRG) classified gastric cancer based on gene expression data into four subtypes: (i) microsatellite instability (MSI), (ii) microsatellite stable and epithelial-to-mesenchymal transition (MSS/EMT), (iii) MSS/TP53 active, and (iv) MSS/TP53 inactive (Cristescu *et al*, 2015). The Singapore classification system distinguished three subtypes: proliferative, metabolic, and mesenchymal (Lei *et al*, 2013). The Cancer Genome Atlas (TCGA) consortium developed a molecular classification system based on observed mutational alterations and grouped gastric cancer into four subtypes (The Cancer Genome Atlas Research Network, 2014). One subtype is characterized by an Epstein Barr virus (EBV) infection and named "EBV-positive" subtype. A second subtype shows a high frequency of MSI and is, therefore, named "MSI" subtype. A third subtype is termed "genomically stable" (GS), displaying a diffuse non-coherent cancer morphology due to the loss of proteins involved in cell adhesion, such as *CDH1*. A fourth molecular subtype, named "chromosomal instability" (CIN) subtype, is characterized by a high number of somatic copy

1 Department of Visceral, Thoracic and Vascular Surgery, Medical Faculty and University Hospital Carl Gustav Carus, Technische Universität Dresden, Dresden, Germany
2 National Center for Tumor Diseases (NCT), Dresden, Germany
3 German Cancer Research Center (DKFZ), Heidelberg, Germany
4 Helmholtz-Zentrum Dresden - Rossendorf (HZDR), Dresden, Germany
5 Proteomics Unit, Spanish National Cancer Research Centre (CNIO), Madrid, Spain
6 Department of Pediatric Oncology, Hematology, and Clinical Immunology, Medical Faculty, University Hospital Düsseldorf, Düsseldorf, Germany
7 Institute of Molecular Biotechnology of the Austrian Academy of Sciences (IMBA), Vienna Biocenter (VBC), Vienna, Austria
8 Center for Genome Engineering, Institute for Basic Science, Daejeon, Republic of Korea
9 Biocruces Bizkaia Health Research Institute, Barakaldo, Spain
10 Ikerbasque, Basque Foundation for Science, Bilbao, Spain
*Corresponding author. Tel: +34 946007967; E-mail: jmunozpe@ext.cnio.es
**Corresponding author. Tel: +49 (0)351 458 2742; E-mail: daniel.stange@uniklinikum-dresden.de
[†]These authors contributed equally to this work

© 2022 The Authors. Published under the terms of the CC BY 4.0 license

number alterations (SCNA). Of note, none of the classification systems up to today influence clinical decision-making, as no convincing link has been established between individual subtypes and certain treatment schemes.

Organoids constitute a three-dimensional (3D) cell culture system directly derived from tissue-resident stem cells (Sato *et al*, 2009). Cells are embedded in an extracellular matrix (ECM) and exposed to growth factors present in the native microenvironment. Organoid cultures show self-renewal, self-organization, and long-term proliferation capacities while faithfully recapitulating many aspects of the tissue they are derived from. Organoids from healthy tissue remain genomically stable over long periods of time (Huch *et al*, 2015; Georgakopoulos *et al*, 2020). Initially developed from intestinal stem cells, protocols have been developed to establish organoids from multiple murine and human organs (Fatehullah *et al*, 2016; Bartfeld & Clevers, 2017). They represent an excellent model system to be employed in a broad range of research topics from basic to translational science, that is, organ development, infection studies, or disease modeling. In addition, patient-derived cancer organoids (PDOs) have been shown to be predictive of the patient's response to a certain anticancer treatment (Vlachogiannis *et al*, 2018; Wensink *et al*, 2021). Each PDO line has an individual pattern of molecular alterations with hundreds of mutations and deregulated signaling pathways. Due to this, they represent unique avatars of a patient, rather than models for a particular cancer (subtype).

We thus established organoid models with a defined mutational spectrum altering specific pathways (Seidlitz *et al*, 2019). Here, these two models were complemented by an additional model and all three were extensively characterized concerning their molecular and functional behavior using proteomics and a drug screen. Specific treatment vulnerabilities were then further validated in PDOs from gastric cancer.

Results

Generation and phenotypic characterization of murine gastric organoid models with defined oncogenic pathway alterations

In order to define the most frequently altered mutations in gastric cancer, we analyzed the TCGA dataset and determined alteration frequencies for the four established molecular subtypes (The Cancer Genome Atlas Research Network, 2014). RTK/MAPK pathway alterations combined with *TP53* mutations are characteristic for the CIN subtype, but can also be found in the MSI and GS subtypes. To model this pathway combination, we coupled inducible alleles of $Kras^{G12D}$ and $Tp53^{R172H}$ (RAS-activated model). Cell motility genes such as *CDH1* are frequently mutated in the GS subtype, which largely overlaps with the diffuse subtype according to Lauren, and are associated with additional activations of oncogenic pathways, that is, the TGFβ, RTK/MAPK, or WNT (Smyth *et al*, 2020). We chose to combine a floxed *Cdh1* with a floxed *Apc* allele to model the diffuse subtype. These two models have been previously established (Seidlitz *et al*, 2019). WNT pathway alterations also frequently occur in other gastric cancer subtypes, with 78% of cases most prominently in the MSI subtype, which also contains *TP53* pathway alterations in 77% of cases (The Cancer Genome Atlas Research Network, 2014). A third organoid model was, therefore, established by combining a floxed *Apc* with an inducible $Tp53^{R172H}$ allele (WNT-activated model). Organoids were generated from the gastric corpus and mutations activated via infection with a Cre/GFP expressing adenovirus (Fig EV1A). They were selected via withdrawal of specific growth factors from the cultivation medium, resulting in the outgrowth of only recombined organoids (Fig EV1B–E).

Normal murine gastric corpus organoids had a cystic structure with a thin single-layered epithelium (Fig 1A1 an A3). As described before, the RAS-activated model displayed a multi-layered irregular epithelium (Fig 1A2 and A4; Seidlitz *et al*, 2019). Contrasting to normal organoids, the $Tp53^{R172H}$ mutation resulted in a nuclear accumulation of TP53 (Fig 1A5 and A6). Due to the EGF in the culture medium, normal organoids showed active epidermal growth factor receptor (EGFR) pathway signaling, demonstrated by phosphorylated nuclear ERK1/2 (Fig 1A7). The $Kras^{G12D}$ mutation in the RAS-activated model resulted in an increase in the ERK1/2 phosphorylation level compared with normal organoids (Fig 1A8). The *Cdh1* loss in the diffuse organoid model led to a complete change in organoid morphology toward a grape-like structure (Fig 1B9–B12; Seidlitz *et al*, 2019). *Cdh1*, which encodes for the cell–cell junction protein E-cadherin, was absent in the diffuse model organoids (Fig 1B13 and B14), and the activation of the WNT pathway resulted in a nuclear accumulation of β-catenin (Fig 1B15 and B16). The newly established WNT-activated model was characterized phenotypically by an irregular mono-layered structure with a rather small organoid size (Fig 1C17–C20). Due to the $Tp53^{R172H}$ mutation and *Apc* deletion, a nuclear accumulation of TP53 and β-catenin could be observed (Fig 1C21–C24).

Cell cycle and proliferation analyses revealed different proliferation rates between the three organoid models. The RAS-activated and WNT-activated organoids contained with 12.7 and 9.4% a higher number of cells within the S-phase, respectively, compared with their normal counterpart (7.4%) (Fig EV2A and B). EdU incorporation assays confirmed this finding, both models also had a significantly higher proliferation rate compared with normal gastric organoids (RAS-activated 36.1%, two-tailed Student's *t*-test $P = 0.031$; WNT-activated 38.1%, $P = 0.0017$; Fig EV2C). The diffuse organoid model showed the lowest number of proliferation with 6.4% of cells in the S-phase and 21% of EdU positivity (normal organoids 26.4%; Fig EV2A–C).

Oncogenic pathway activations resulted in individual proteome signatures

To understand the underlying molecular biology present in each organoid model, we conducted global proteomic analyses (Fig 2A–C, Dataset EV1). We analyzed the proteomes of the altered organoids

Figure 1. Immunohistological characterization of the organoid models.

A Brightfield pictures of normal versus RAS-activated organoids (1 and 2), HE staining (3 and 4), TP53 (5 and 6) and pERK1/2 (7 and 8) (scale bar 15 μm).
B Brightfield pictures of normal versus diffuse organoids (9 and 10), HE staining (11 and 12), E-cadherin (13 and 14), and β-catenin (15 and 16) (scale bar 15 μm).
C Brightfield pictures of normal versus WNT-activated organoids (17 and 18), HE staining (19 and 20), TP53 (21 and 22), and β-catenin (23 and 24) (scale bar 15 μm).

 © 2022 The Authors

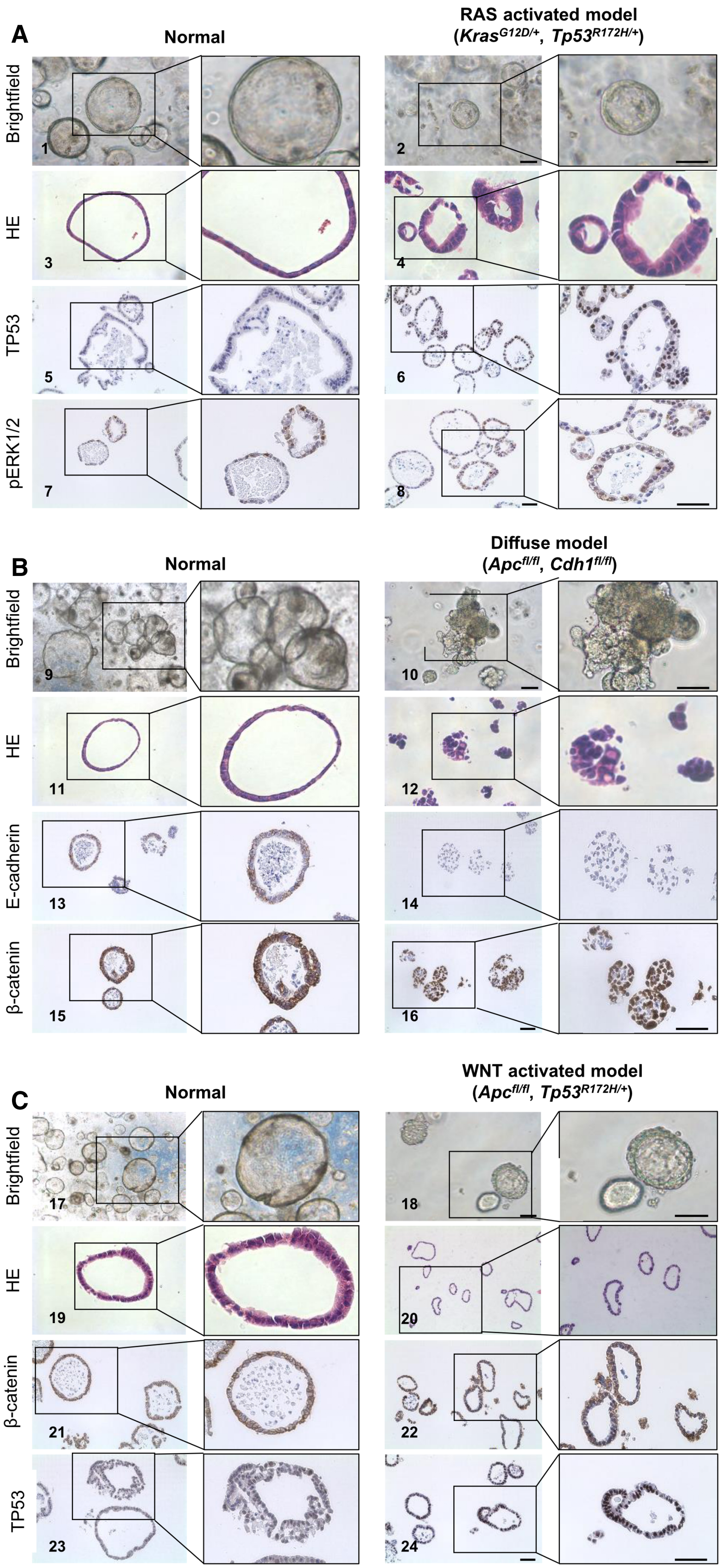
A
Normal
RAS activated model
(KrasG12D/+, Tp53R172H/+)
Brightfield
HE
TP53
pERK1/2
1
2
3
4
5
6
7
8
B
Normal
Diffuse model
(Apcfl/fl, Cdh1fl/fl)
Brightfield
HE
E-cadherin
β-catenin
9
10
11
12
13
14
15
16
C
Normal
WNT activated model
(Apcfl/fl, Tp53R172H/+)
Brightfield
HE
β-catenin
TP53
17
18
19
20
21
22
23
24

Figure 1.

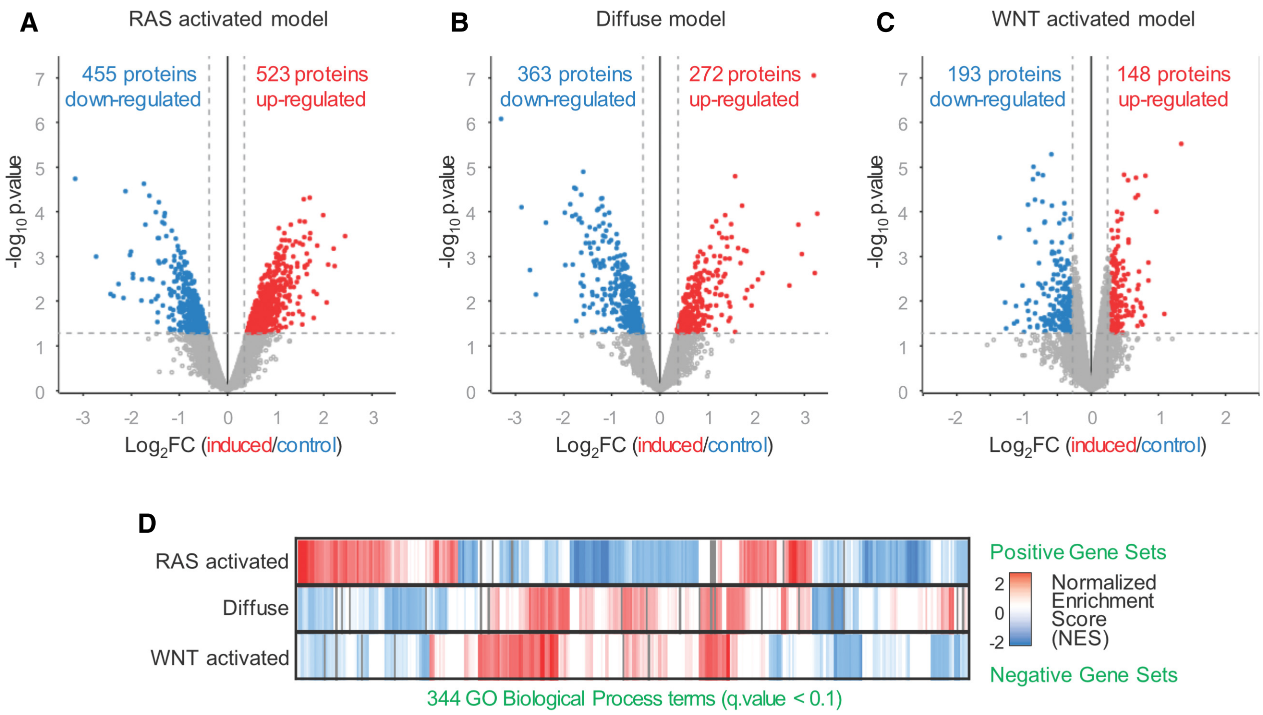

Figure 2. Proteomic characterization of organoid models.

A–C Volcano plots of proteomic analyses of the organoid models. Plots indicate up- and downregulated proteins compared with uninduced controls (biological replicates n = 2–3).

D Comparison of significantly altered biological processes (Gene Ontology) identified by gene set enrichment analyses (GSEA) (q-value < 0.1) between the organoid models.

with respect to the normal (uninduced) organoids of the same genotype. The RAS-activated model showed an altered expression in 978 proteins (455 down- and 523 upregulated) compared with normal gastric organoids (Fig 2A). In the diffuse organoid model, 635 proteins were differentially expressed (363 down- and 272 upregulated; Fig 2B), and in the WNT-activated organoid model, 341 differentially expressed proteins were found (193 down- and 148 upregulated; Fig 2C). As expected, a significantly downregulation of the CDH1 protein was found in the diffuse organoid model (Log2 diffuse/normal −1.595, Limma significance = DOWN < −0.4, P-value = < 0.05; Dataset EV1). Activating the WNT pathway by *Apc* deletion resulted in a significant upregulation of the WNT target gene matrix metallopeptidase 7 (MMP7; Log2 WNT-activated/normal 0.624, Limma significance = UP > 0.4, P-value = < 0.05) in the WNT-activated model, which was not seen in the diffuse model (Dataset EV1).

To understand the proteomic changes present in each model, we performed gene set enrichment analysis (GSEA) of Gene Ontology (GO) terms and found a large number of biological processes displaying both upregulated and downregulated proteins across the three different models (q-value < 0.1; Fig 2D). In detail, the RAS-activated model carrying the R172H mutation in the tumor suppressor *Tp53* showed a downregulation of GO term "cell cycle phase control" (Normalized Enrichment Score (NES) −2.26, q-value < 0.000; Fig 3A; Dataset EV2). Importantly, this was not seen for the WNT-activated model (NES 1.02, q-value = 0.743) harboring the same $Tp53^{R172H}$ mutation. The diffuse model also showed no altered cell cycle control (NES 1.44, q-value = 0.276). Furthermore, in the RAS-activated model a downregulation of the GO term "double strand break repair" (NES −2.01, q-value = 0.007) was seen (Dataset EV2). This was again not observed in the WNT-activated organoids (NES 1.32, q-value = 0.40). For the WNT-activated model, a significant increase in the GO term "nuclear DNA replication" was observed (NES 1.93, q-value = 0.02), while the RAS-activated organoids showed the opposite pattern (NES −1.72, q-value = 0.008; Dataset EV2). An activation of translational processes was detected in the RAS-activated organoids, that is, elongation (NES 2.26, q-value < 0.000) and termination (NES 2.14, q-value = 0.002). This was not recognized for the diffuse (NES −1.39, q-value = 0.316) and WNT-activated models (NES −1.13, q-value = 0.563; Fig 3B; Dataset EV2). The diffuse organoids with a loss of E-cadherin showed a significantly increased adherens junction assembly (NES 1.99, q-value = 0.039; Fig 3C; Dataset EV2). On the single protein level, we could observe a significant upregulation of the cell adhesion molecules podocalyxin (PODXL), MUC18 (Melanoma Cell Adhesion Molecule (MCAM)), and thrombospondin-1 (THBS1; Dataset EV1). At the same time, a downregulation of the desmosome proteins desmoglein-2 (DSG2) and desmocollin-2 (DSC2) as well as the integrins ITGA1 and ITGA7 could be observed (Dataset EV1). Thus, the loss of CDH1 seems to disarrange the adherens signaling pathways of proteins involved in its assembly.

Interestingly, the WNT-activated model presented an activation of the adenosine triphosphate (ATP)-dependent chromatin remodeling (NES 2.11, q-value = 0.006). This was not observed for the other organoid models (Fig 3D; Dataset EV2). Overall, the proteomic data of the three gastric organoid models revealed unique patterns of differentially expressed proteins, which resulted in strikingly diverse

 © 2022 The Authors

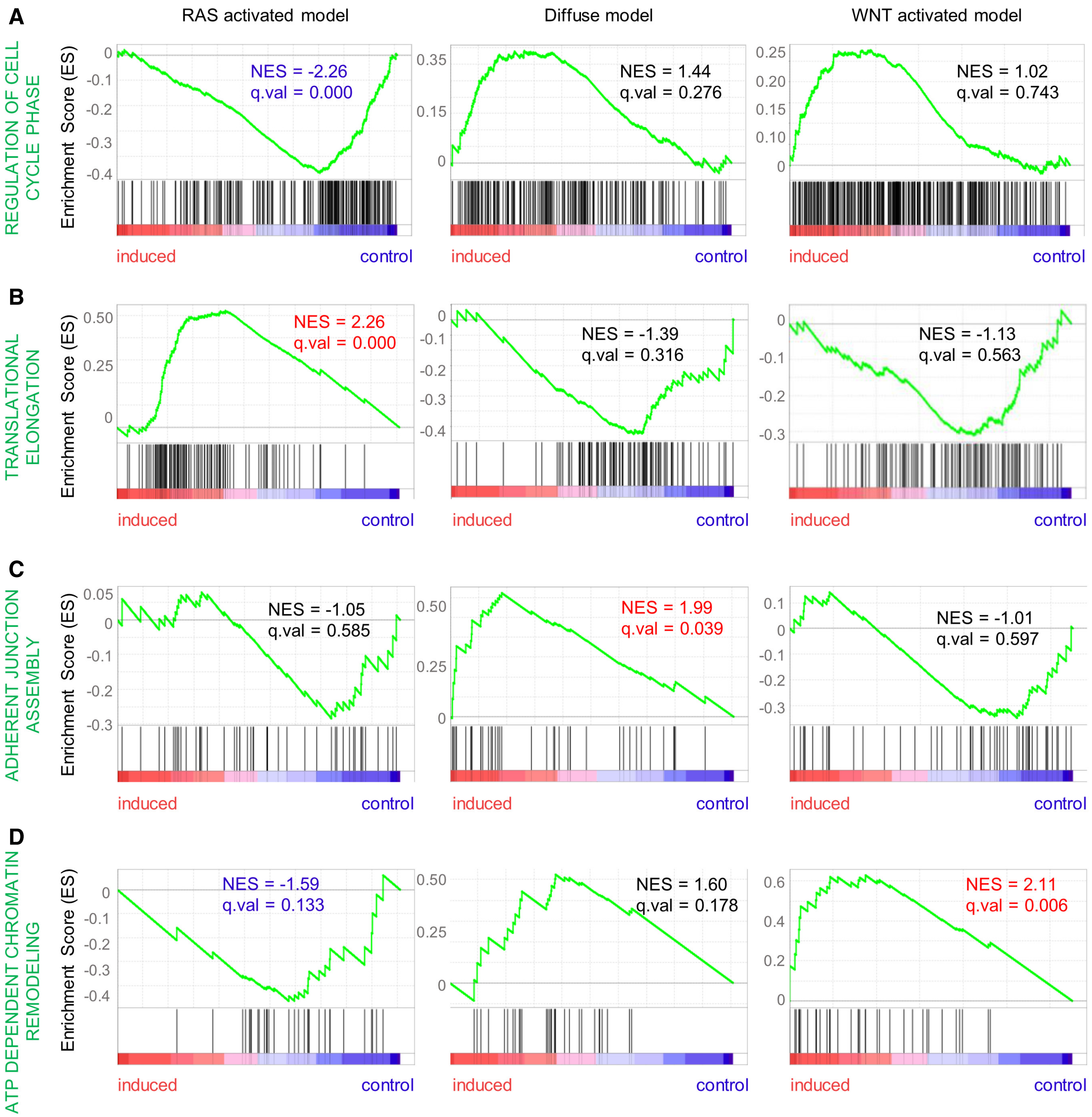

Figure 3. Differentially deregulated biological processes.

A–D Charts of normalized enrichment scores (NES) for differentially deregulated GO biological processes between the organoid models: (A) regulation of cell cycle phase, (B) translational elongation, (C) adherent junction assembly, and (D) ATP-dependent chromatin remodeling.

individual signatures of activated biological processes for each model.

Identification of individual vulnerabilities by drug screening of organoid models

To investigate the differences between treatment responses of the three gastric organoid models, we treated both normal and model organoids with a drug library composed of 196 different compounds including classical chemotherapeutics and targeted drugs (Fig 4A, Dataset EV3). While divergent treatment responses could be observed for the three models, the positive (staurosporine, protein kinase inhibitor preventing the binding of ATP to kinase domains) and negative (DMSO) controls showed the expected results. As a mutation-based positive control, response was analyzed to nutlin-3a treatment, a *MDM2* inhibitor causing accumulation of TP53 and

© 2022 The Authors

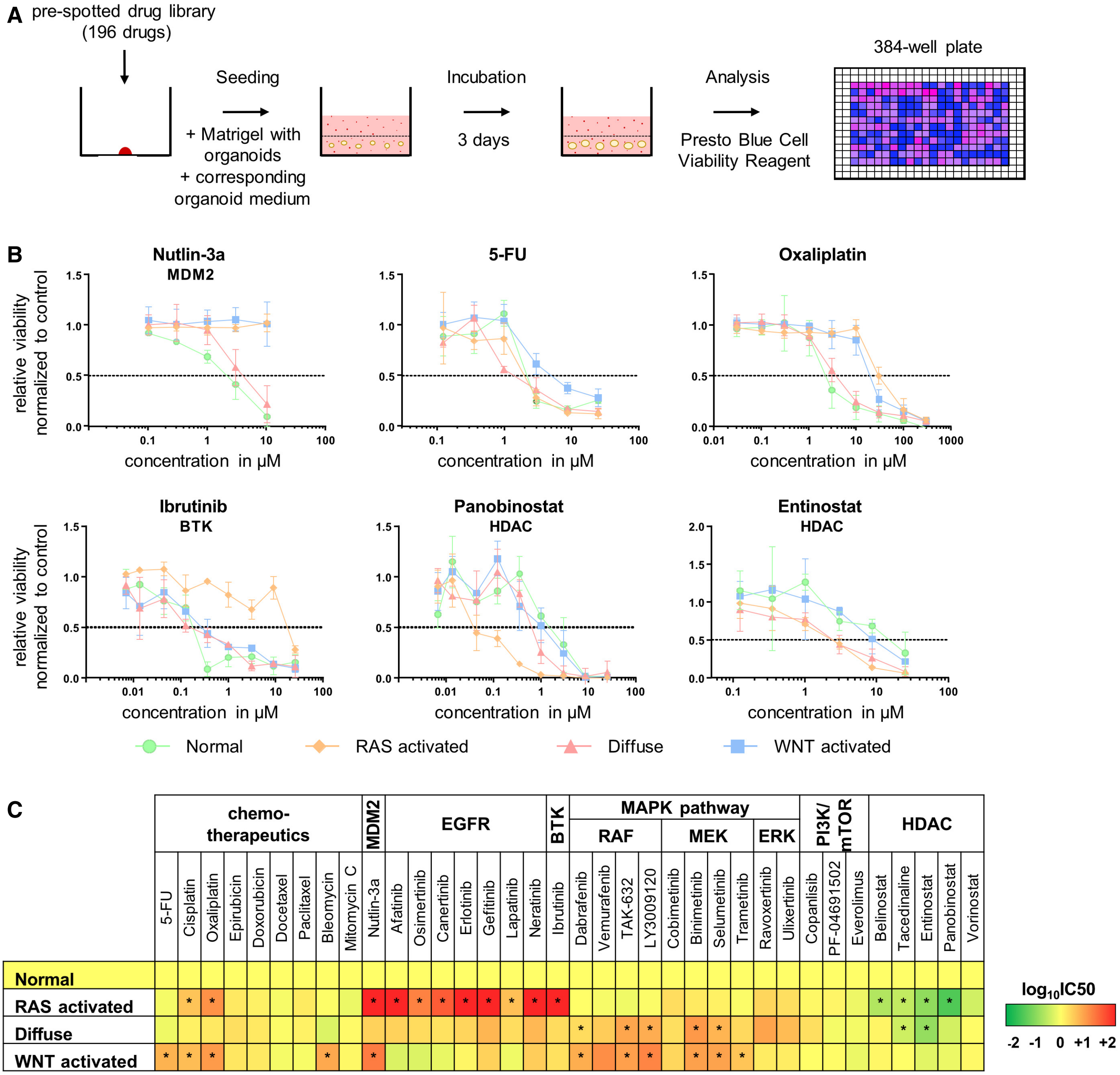

Figure 4. Varying therapy responses depending on altered signaling pathways.

A Scheme of the drug library screen of organoid models.

B Drug response curves upon treatment of organoid models with nutlin-3a, 5-FU, oxaliplatin, ibrutinib, panobinostat, and entinostat (biological replicates *n* = 3, data are shown as mean ± SD).

C Heat map summarizing results of drug library screen categorized by pathway. Color scale indicates log_{10}IC50 change in the organoid models compared with normal gastric organoids. Two-tailed Student's *t*-test organoid model versus normal organoids (*< 0.05).

thus growth inhibition (Matano *et al*, 2015). Both organoid lines carrying the *Tp53*^{R172H} mutation, the RAS- and the WNT-activated model, were not responsive to nutlin-3a treatment, while the two lines with wild-type *Tp53* showed a clear response (Fig 4B and C).

Next, the response to classical chemotherapeutics was tested. Compared with normal organoids, the RAS-activated and WNT-activated organoids were significantly less responsive to the platinum compounds oxaliplatin (two-tailed Student's *t*-test; RAS: $P = 0.001$; WNT $P = 0.004$) and cisplatin (two-tailed Student's *t*-test; RAS: $P = 0.013$; WNT $P = 0.010$; Figs 4B and C, and EV3A; Dataset EV3). The WNT-activated organoids were resistant toward 5-FU and bleomycin. No differences in therapy response between the organoid lines were seen for epirubicin, doxorubicin, docetaxel, paclitaxel, and mitomycin C. Subsequently, the response toward

 © 2022 The Authors

targeted drugs was evaluated. The RAS-activated organoids showed a unique resistance pattern toward the bruton tyrosine kinase (BTK) inhibitor ibrutinib, whereas the other organoid models already responded at a dose of approx. 0.1 μM (Fig 4B and C; Dataset EV3). The RAS-activated and partially also the diffuse model showed increased sensitivity to histone deacetylase (HDAC) inhibitors compared with normal gastric organoids. While the RAS-activated organoids were sensitive to all tested HDAC inhibitors, the sensitivity of the diffuse organoids was restricted to entinostat and tacedinaline. The WNT-activated organoid model showed similar response patterns toward all HDAC inhibitors as the normal organoids (Figs 4B and C, and EV3B; Dataset EV3). Interestingly, the GSEA analysis showed specifically for the RAS-activated organoids a trend of downregulation, although not significant, in several biological processes related to chromatin: "chromatin organization" (NES −1.44, q-value = 0.214), "chromatin remodeling" (NES −1.37, q-value = 0.259), "ATP dependent chromatin remodeling" (NES −1.59, q-value = 0.133), "histone deacetylation" (NES −1.18, q-value = 0.434) and "regulation of histone modification" (NES −1.18, q-value = 0.434) in the RAS-activated organoids (Dataset EV2). It thus seems that the introduced mutations in the RAS-activated organoid line have an impact on chromatin structure and modification. Of note, inhibitors targeting the PI3K/mTOR signaling pathway did not result in differential responses between the models and normal organoids (Figs 4C and EV3C; Dataset EV3). In summary, the response data toward classical chemotherapeutics as well as specific inhibitors revealed vulnerabilities of each model toward individual drugs or drug families.

Variable response upon EGFR/MAPK pathway inhibition in *Kras*^{G12D}-driven organoids

The EGFR/MAPK pathway is one of the most frequently deregulated signaling pathways in gastric cancer (Fig 5A; The Cancer Genome Atlas Research Network, 2014). Targeting this pathway led to varying responses of the different organoid models (Fig 4C). As expected, the RAS-activated organoids were resistant toward EGF receptor inhibitors (Figs 4C, 5B and EV3D; Dataset EV3). Of note, the diffuse and WNT-activated model showed a consistent tendency of a favorable response toward EGFR inhibition. Inhibition of the pathway further downstream by blocking RAF with wild-type B-RAF inhibitors (LY3009120 and TAK-632) reversed the response pattern: RAS-activated organoids were as sensitive as normal organoids, while the diffuse and WNT-activated organoids were significantly less responsive (Figs 4C and 5C; Dataset EV3). Interestingly, also B-RAF^{V600E} inhibition (vemurafenib and dabrafenib) showed a higher sensitivity of RAS-activated and normal organoids; nevertheless, the observed differences were more prominent for the wild-type B-RAF inhibitors (Fig 5C). A similar response pattern, albeit less pronounced, was observed when targeting MEK1/2 with selumetinib, binimetinib, cobimetinib, or trametinib (Figs 4C, 5D and EV3E; Dataset EV3). Targeting the MAPK pathway one step further "down" the signaling cascade, at the level of ERK1/2 (ravoxertinib and ulixertinib), no significant differences in treatment response could be observed (Figs 4C and 5E; Dataset EV3). Combined, targeting the EGFR/MAPK pathway at different levels revealed that the RAS-activated organoid model could be inhibited best close to the mutational activation on the level of B-RAF. *Apc*-mediated WNT pathway activation (in the diffuse and WNT-activated models) was associated with a decreased sensitivity to MAPK pathway inhibitors.

Gastric cancer PDOs show diverging responses to MEK1/2 and HDAC inhibition

To follow up on the observation, that the RAS-activated organoids were sensitive to all tested HDAC inhibitors and extend the analysis to human cancer, PDOs from gastric cancer were investigated (Fig 6A). PDOs have been shown previously to maintain patient individual including growth characteristics (Seidlitz *et al*, 2019). In line with this, PDOs used in this study recapitulated histologically the tissue of origin (Fig EV4). For example, mixed morphologies, that is, intestinal and diffuse growing patterns in the primary tissue of DD483 were also found in the corresponding organoid culture, which showed cystic shapes typical for intestinal tumors as well as poorly adhering cell clusters characteristic for diffuse tumors. PDOs were classified based on the presence or absence of alterations in the RTK/MAPK pathway (see Expanded View Materials and Methods for details). Six out of the 13 PDOs contained an EGFR or HER2 overexpression, frequently found in gastric cancer (The Cancer Genome Atlas Research Network, 2014), or pathogenic mutations in downstream members of the RTK/MAPK pathway (Table EV1). In total, 13 PDO lines were subsequently analyzed with regard to their response to the MEK1/2 inhibitor trametinib, the pan-EGFR inhibitor afatinib, and the B-RAF inhibitor LY3009120 (Figs 6B and EV5A) as well as the HDAC inhibitors panobinostat, entinostat, tacedinaline, and vorinostat (Figs 6C and EV5B, Table EV2). A set of two gastric PDOs from healthy stomach mucosa ("normal PDOs") served as controls. Overall, PDOs showed diverging dose–response patterns to tested inhibitors (Figs 6B and C, and EV5A and B, Table EV2). Compared with cancer PDOs, normal gastric organoids tended to be more responsive to MEK1/2 inhibition with trametinib (area under curve $(AUC)_{rel}$: 0.438 (normal) vs. 0.614 (PDOs)), while they tended to be less responsive to HDAC inhibitors (AUC_{rel} normal vs. PDOs: panobinostat: 0.582 vs. 0.524, entinostat: 0.581 vs. 0.563, tacedinaline: 0.628 vs. 0.598, and vorinostat: 0.576 vs. 0.554) (Table EV3). Similar to MEK1/2 inhibition, normal gastric organoids were more sensitive than cancer PDOs toward EGFR (AUC_{rel}: 0.449 (normal) vs. 0.861 (PDOs)) and B-RAF inhibition (AUC_{rel}: 0.653 (normal) vs. 0.895 (PDOs)). Within the cancer PDOs, a significantly higher sensitivity of PDOs with altered versus unaltered RTK/MAPK pathway toward MEK1/2 inhibition by trametinib could be observed (Fig 6D) (AUC_{rel} (RTK/MAPK-unaltered): 0.7101; AUC_{rel} (RTK/MAPK-altered): 0.5308; two-tailed Student's t-test; $P = 0.0356$). This proofed functionally that RTK/MAPK pathway alterations indeed increased the sensitivity to an interference with the pathway on the level of MEK1/2. Nevertheless, not all RTK/MAPK-altered PDOs were susceptible compared with unaltered PDOs. Furthermore, upstream intervention using afatinib ($P = 0.7588$) or LY3009120 ($P = 0.2612$) did not result in a difference between the PDOs with different RTK/MAPK alteration status (Fig EV5A and C). These findings underline the difficulty of predicting a response based on the presence of a certain alteration due to the large number of further mutations in the genome of cancer PDOs.

Similar to trametinib, a tendency of a higher HDAC inhibitor resistance was seen in RTK/MAPK pathway unaltered PDOs (Figs 6E and EV5B and D). In order to compare AUC_{rel}s of different

© 2022 The Authors

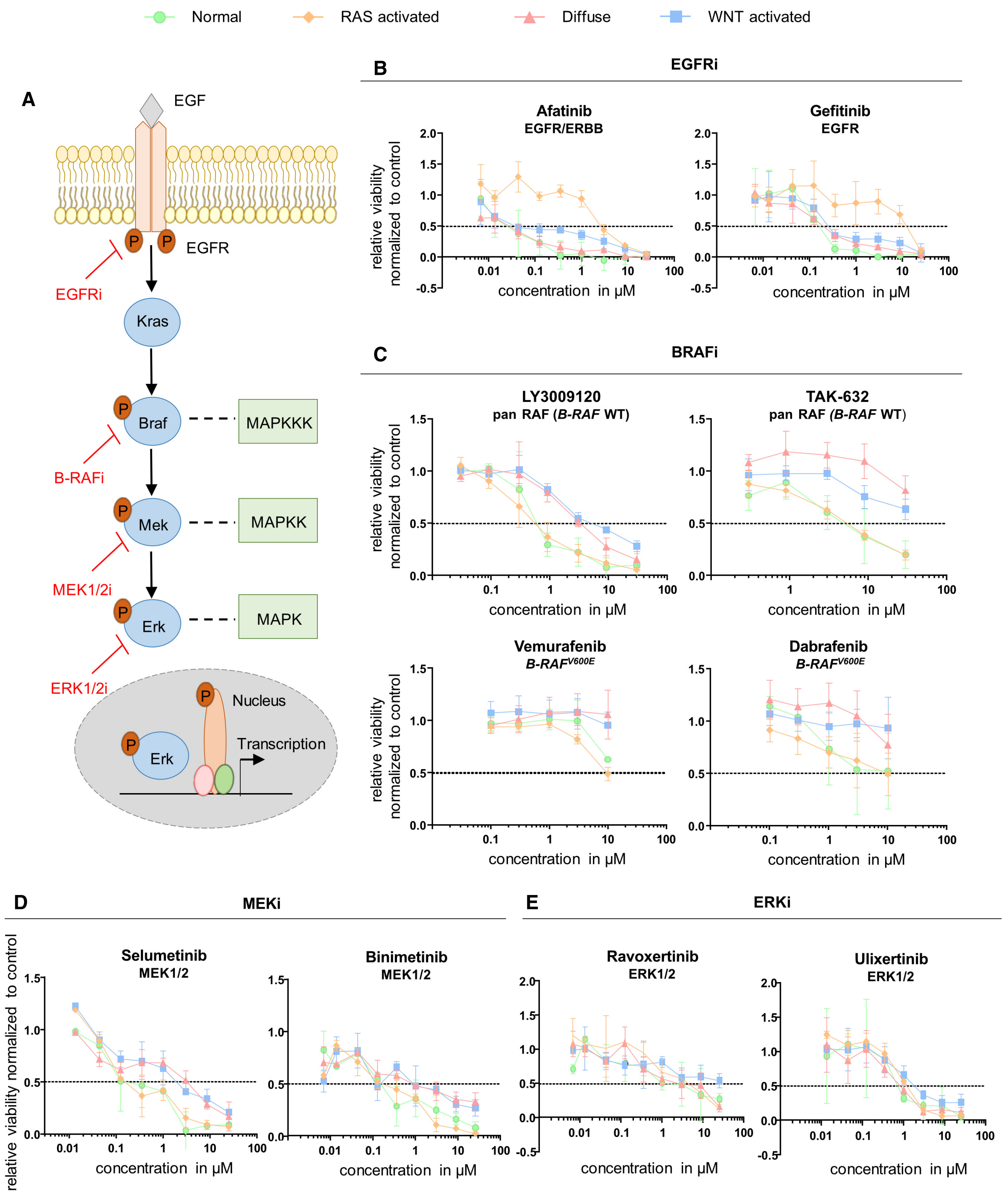

Figure 5. Targeting the EGFR/MAPK pathway at different levels of the signaling cascade.

A Scheme of EGFR/MAPK signaling pathway and targeting inhibitors.

B–E Drug response curves upon treatment with inhibitors for (B) EGFR (afatinib and gefitinib), (C) B-RAF (LY3009120, TAK-632, vemurafenib, dabrafenib), (D) MEK1/2 (selumetinib and binimetinib) and (E) ERK1/2 (ravoxertinib and ulixertinib) (biological replicates $n = 3$, data are shown as mean ± SD).

© 2022 The Authors

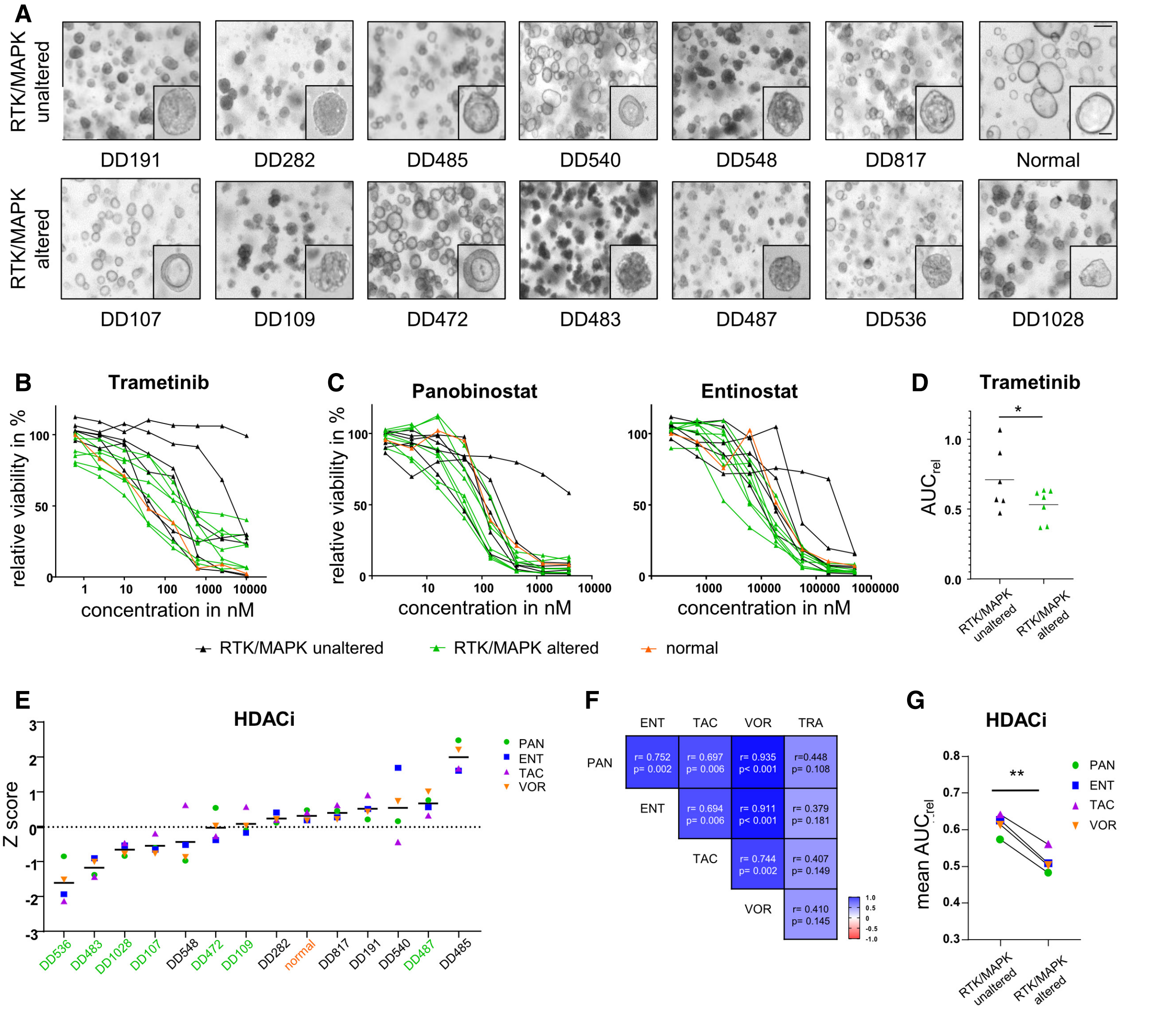

Figure 6. Treatment of human patient-derived organoids with MEK1/2 and HDAC inhibitors.

A Brightfield images of human patient-derived organoids (PDOs) from gastric cancer with and without RTK/MAPK alterations (scale bar 100 μm, zoom in 40 μm).

B Drug response curves of PDOs upon treatment with the MEK1/2 inhibitor trametinib (biological replicates $n = 3$, data are presented as mean, SD values are shown in Table EV2).

C Drug response curves upon HDAC inhibition with panobinostat (PAN) and entinostat (ENT) (biological replicates $n = 3$, data are presented as mean, SD values are shown in Table EV2).

D Comparison of the relative area under the curve (AUC_{rel}) upon trametinib treatment in RTK/MAPK-altered (mean AUC_{rel}: 0.5308; biological replicate $n = 7$) versus non-altered (mean AUC_{rel}: 0.7101; biological replicates $n = 6$) human PDOs (two-tailed Student's t-test; $*P = 0.0356$).

E Z scores from PDOs and normal organoids treated with HDAC inhibitors (green: PDOs with RTK/MAPK alterations; black: PDOs without RTK/MAPK alterations; orange: normal PDOs).

F Correlation coefficients for the MEK1/2 inhibitor trametinib and the HDAC inhibitors PAN, ENT, tacedinaline (TAC) and vorinostat (VOR) by Pearson correlation.

G Comparison of the mean AUC_{rel} of all RTK/MAPK-altered versus RTK/MAPK-unaltered human PDOs upon HDAC inhibition with PAN, ENT, TAC and VOR (biological replicates $n = 4$; two-tailed paired Student's t-test; $**P = 0.0013$).

drugs, a Z-transformation of AUC_{rel} values was performed (Fig 6E, Table EV3). This analysis underlined the correlation between HDAC resistance and RTK/MAPK pathway alterations: Out of the six most resistant PDOs, five were RTK/MAPK-unaltered (labeled in black), while 6/7 most sensitive PDOs were RTK/MAPK-altered (labeled in green). Furthermore, this analysis revealed for some PDOs variations in individual sensitivities to different HDAC inhibitors. Exemplarily, DD540 exhibited a wide response range, with a sensitivity to tacedinaline (Z score: −0.559), moderate response to panobinostat (Z score: −0.033) and vorinostat (Z score: 0.489), while being highly resistant against entinostat (Z score: 1.592). Thus, while for most PDOs a common response to different HDAC inhibitors could be documented, some PDOs depicted a profound heterogeneity in terms of response within the drug class of HDAC inhibitors. The overall concordance in response was substantiated by correlation analyses of the different HDAC inhibitors: All correlation coefficients

© 2022 The Authors

were highly positive (Fig 6F). Furthermore, the correlation coefficient of each HDAC inhibitor with trametinib also showed a positive tendency (panobinostat: $r = 0.45$, $P = 0.108$; entinostat: $r = 0.36$, $P = 0.181$; tacedinaline: $r = 0.41$, $P = 0.149$; vorinostat: $r = 0.41$, $P = 0.145$; Fig 6F). Overall, the group of RTK/MAPK-altered PDOs showed a significantly higher sensitivity to HDAC inhibition compared with the group of non-altered RTK/MAPK PDOs (two-tailed paired Student's *t*-test, $P = 0.0013$; Fig 6G). In summary, the analyses of human gastric cancer PDOs confirmed the findings from the RAS-activated murine organoid model and substantiated the association between the presence of an RTK/MAPK alteration and HDAC inhibitor sensitivity.

Discussion

Human PDOs as avatars of a patient's tumor hold great promise for therapy response prediction with the long-term goal to improve the survival of cancer patients. However, using PDOs as model systems often results in difficult to interpret data, as each PDO carries an individual set of between a few hundred to several thousand mutations. Targeting a single pathway in individual PDOs with a certain pathway alteration results in variable responses depending on the activation state of other pathways. The resulting data can, therefore, often not be generalized, but need to be interpreted bearing in mind the singularity of the analyzed tumor. We, therefore, set out to generate organoid models with a defined mutational pattern by manipulating pathways commonly altered in gastric cancer subtypes (The Cancer Genome Atlas Research Network, 2014). To this aim, we combined different floxed alleles in mice, generated organoids, and activated or deleted genes by Cre recombination *in vitro*.

The generated three organoid models exhibited different morphologies. The RAS-activated organoids (*Kras*^{G12D}; *Tp53*^{R172H}) had a cystic structure with a thickened lumen compared with normal organoids. Organoids of the diffuse model (*Cdh1*$^{fl/fl}$; *Apc*$^{fl/fl}$) showed a complete change in morphology induced by the loss of cell–cell connections, resulting in a grape-like growth pattern. The WNT-activated organoids (*Apc*$^{fl/fl}$; *Tp53*^{R172H}) were characterized by an irregular thin-layered epithelial structure with a smaller organoid size compared with the other lines. Interestingly, proliferation analysis showed differential growth rates, with the RAS-activated and WNT-activated organoids proliferating significantly faster than normal organoids.

To characterize these organoid models at the molecular level, we analyzed changes in protein abundance levels. The use of the normal uninduced counterparts as a reference allowed to study the effect of each mutational pattern in the same background. All three organoid models altered the expression of a significant fraction of their proteome, affecting multiple processes and functions. Importantly, we found numerous proteins uniquely affected in each model, providing molecular signatures that form the basis of the phenotypic differences existing between them. For instance, the RAS pathway activation in combination with *Tp53*^{R172H} loss of function resulted in a downregulation of proteins involved in cell cycle phase control and double-strand break repair. Surprisingly, this was not found in the WNT-activated organoids, which carry the same *Tp53*^{R172H} mutation. This organoid model showed a unique upregulation of DNA packaging, mismatch repair, and replicative processes. The diffuse model with the loss of the cell–cell junction protein E-cadherin showed an increased adherent junction assembly. Similar observations were made by Chen *et al* (2014). Loss of *CDH1* in the breast cancer cell line MCF10A by zinc finger nuclease technology resulted in altered expression of cell–cell adhesion genes (Chen *et al*, 2014). Furthermore, the Qin laboratory analyzed 84 human diffuse gastric cancer for their proteome signatures. One identified subtype of diffuse gastric cancer, the immunological enriched subtype, also showed an enrichment of adhesion pathways (Ge *et al*, 2018). Our observed upregulation of adherens junction assembly is thus in line with published data and suggests a possible compensation mechanism induced by the loss of CDH1 function via upregulation of various proteins and pathways involved in cell adhesion.

In order to evaluate functional effects of the pathway alterations and the resulting changed proteome composition, we performed a medium-scale drug screen with 196 different compounds. Classical chemotherapy treatment in the different organoid lines showed a divergent therapy response. For example, the RAS-activated and WNT-activated models were less responsive to platinum compounds. Eventually, the *Tp53* alteration lead to the observed resistance in both organoid models as it has been previously described in other entities (Gadducci *et al*, 2002; Lin & Howell, 2006; Tung *et al*, 2015). The WNT-activated model showed a resistance to treatment with 5-FU, while the diffuse model with the same activating *Apc* mutation was sensitive to the treatment. Thus, the addition of only one more alteration to a WNT pathway activation can significantly influence the response to a widely used chemotherapeutic drug. In general, organoids of the diffuse model showed an increased sensitivity to classical chemotherapies compared with the RAS- and WNT-activated models.

Classic chemotherapy is still the backbone of gastric cancer treatment up to today. However, targeted therapies are expected to accompany them increasingly in future (Samson & Lockhart, 2017). To reach this aim, further research is necessary to analyze the effects and relationships of deregulated signaling pathways on therapy response. We, therefore, tested a drug library on the three tumor organoid models that contained small molecules against a wide range of cancer-associated pathways, that is, EGFR, MAPK, BTK, and PI3K/mTOR. As a mutation-based positive control, the MDM2 inhibitor nutlin-3a was included. MDM2 negatively regulates TP53 by mediating the ubiquitin-dependent degradation of TP53 (Michael & Oren, 2003; Toledo & Wahl, 2007). Organoids with a *Tp53* mutation were as expected resistant to nutlin-3a treatment (Matano *et al*, 2015). No specific sensitivity or resistance could be observed for inhibitors of the PI3K/mTOR pathway. This is interesting, as it is well established that oncogenic RAS activates besides the MAPK, the PI3K/mTOR pathway (Castellano & Downward, 2011). At least in the generated model system, the activation of *Kras*^{G12D} does not engage the PI3K/mTOR pathway to a level that it can be inhibited by the applied drugs.

The EGFR/MAPK signaling pathway plays a crucial role in the regulation of various cellular activities like proliferation, survival, and differentiation. The pathway is one of the most frequently deregulated signaling pathways in cancer cells. Concerning gastric cancer, amplifications of receptor tyrosine kinases of the ERBB family are observed in about 22% of gastric cancer patients (Bang *et al*, 2010), resulting in an activation of the downstream RAS

 © 2022 The Authors

pathway. The signaling pathway, therefore, constitutes a promising therapeutic target in gastric cancer, which is already therapeutically targeted in HER2-positive cancers. Of note, the EGFR/MAPK pathway can be inhibited at different levels, that is, the level of EGFR, B-RAF, MEK1/2, and ERK1/2. In the RAS-activated model, we activated the pathway using a *Kras*^{G12D} mutation, activating the pathway downstream of the receptor level. In line with this, the RAS-activated model was resistant to inhibitors targeting the EGFR family compared with organoids with wild-type *Kras*. Similar response patterns have also been described for *KRAS*-mutated colorectal and lung cancer (Dempke & Heinemann, 2010; Carter & Giaccone, 2012; Zhao *et al*, 2017). Further downstream inhibition of the RAS pathway with B-RAF or MEK1/2 inhibitors counteracted the *Kras*^{G12D} mutation and resulted in a comparable sensitivity of RAS-activated organoids and normal organoids, which also depend on signaling through this pathway. Of note, a paradoxical activation of ERK signaling after targeting with B-RAF^{V600E} inhibitors (vemurafenib and dabrafenib) was documented by others for *RAS*-mutated and *B-RAF* wild-type tumor cells (Hatzivassiliou *et al*, 2010; Poulikakos *et al*, 2010). We did not observe this phenomenon in our RAS-activated model. Interestingly, the two models carrying an *Apc* mutation (the diffuse and WNT-activated models) were resistant to the B-RAF and MEK1/2 treatment. This indicates that they have become less dependent of the RAS pathway signaling compared with normal organoids. One possible explanation might be that the activation of the WNT pathway compensates for the interference in the RAS pathway by activating different pro-proliferative and anti-apoptotic genes. Of note, at the level of ERK1/2, no differential response between the organoid models and normal organoids could be observed.

An interesting drug response pattern could be seen for the family of HDAC inhibitors. This class of drugs emerged for different cancer entities as promising anticancer agents, particularly in combination with other chemo- or radiotherapy regimens (Suraweera *et al*, 2018). They block gene transcription and induce cell cycle arrest as well as apoptosis. Surprisingly, the RAS-activated organoids showed a significantly increased sensitivity to HDAC treatment (i.e., belinostat, tacedinaline, entinostat, and panobinostat). The strongest effect was observed for panobinostat, a pan-deacetylase inhibitor. The diffuse gastric model also showed a significantly increased sensitivity to HDAC inhibition, but only to the class I HDAC inhibitors tacedinaline and entinostat. Interestingly, the RAS-activated organoids showed a trend of downregulation of several biological processes related to chromatin organization and histone modification. One hypothesis is that these processes are (partly) mediated by HDACs as part of pivotal adaption processes in MAPK-activated gastric cancer to silence tumor suppressors and prevent cell cycle arrest. This adaption process might be reset by HDAC inhibitors. Future research looking into chromatin remodeling is necessary to reveal the molecular mechanisms behind the HDAC inhibitor sensitivity.

To evaluate whether the observed sensitivity of the murine RAS-activated organoids toward HDAC inhibition can be translated to human disease, we made use of our biobank of gastric cancer PDOs (Seidlitz *et al*, 2019). We started out by analyzing the PDO response to the MEK1/2 inhibitor trametinib. PDOs harboring RTK/MAPK pathway alterations displayed a sensitivity to trametinib, comparable to organoids from healthy gastric mucosa as both rely on this mitogenic pathway. Associations between pathway dependence and corresponding sensitivity to downstream interference have been previously described by Wagle and colleagues revealing MEK1/2 inhibitor sensitivity as an indicator of MAPK pathway dependence (Wagle *et al*, 2018). Interestingly, RTK/MAPK-unaltered PDOs were mostly resistant to MEK1/2 inhibition, suggesting a decreased dependence on the MAPK signaling pathway compared with normal organoids. This independence is likely due to mitogenic mutations in other signaling pathways present in the PDOs that overcome MAPK pathway interference. In line with this, our murine organoid models carrying a WNT pathway activating *Apc* mutation were also less affected by MEK1/2 activity blockage. Such compensatory interactions with the MAPK pathway have been described for the PI3K/mTOR signaling axis (Wee *et al*, 2009). PI3K/mTOR pathway-mediated adaption is suggested to be the reason for the failure of MEK1/2 inhibitors in *KRAS*-mutated cancers (Jänne *et al*, 2017; Van Cutsem *et al*, 2018). This hypothesis is supported by the promising data of combinatorial interventions targeting both MAPK and PI3K/mTOR pathway in *RAS*-driven tumors, which might overcome compensatory mechanisms between these two pathways (Merz *et al*, 2021).

Next, we investigated the response of gastric cancer PDOs to HDAC inhibition. Interestingly, similar to the RAS-activated murine organoids RTK/MAPK-altered PDOs showed an overall higher sensitivity to HDAC inhibitors compared with RTK/MAPK-unaltered PDOs. We could thus demonstrate both in the murine as in the human system an association between RTK/MAPK pathway activation and sensitivity to HDAC inhibition. To our knowledge, RTK/MAPK pathway activation has not yet been linked in a cellular system to higher HDAC inhibitor sensitivity. In future, a combinatorial treatment with a MEK1/2 inhibitor and a HDAC inhibitor could lead to increased responses in patients with RTK/MAPK-altered gastric adenocarcinoma. Interestingly, such an approach has also been suggested for *RAS*-driven lung cancer (Yamada *et al*, 2018). Of note, it is currently unclear which HDAC inhibitor class has the highest potential. Our current data show for some PDOs a high variability in their response toward different HDAC inhibitors, suggesting that an individual *in vitro* testing would remain necessary.

In summary, we characterized a set of murine gastric organoid models with common combinations of altered signaling pathways present in subtypes of gastric cancer. The generated models revealed according to their mutational pattern altered phenotypic characteristics, varying proliferation rates, diverse protein signatures, and a divergent therapy response. We outlined a changing vulnerability to RTK/MAPK pathway interference based on the different mitogenic drivers. Furthermore, an association between MAPK pathway activity and susceptibility to HDAC inhibition could be established, uncovering a potential novel treatment approach for RTK/MAPK pathway altered gastric cancer patients.

Materials and Methods

Mouse gastric organoid generation, cultivation, and adenoviral infection

Mouse gastric organoids were generated from three different mouse lines and cultured as previously described (Stange *et al*, 2013).

© 2022 The Authors

Organoids of the first model (*Kras*$^{G12D/+}$; *Tp53*$^{R172H/+}$; RAS-activated model) contain an inducible allele of *Kras*^{G12D} (Kras^{tm4Tyj}) and an inducible allele of *Tp53*^{R172H} (Tp53^{tm2Tyj}) (Jackson *et al*, 2001; Olive, 2004). The second model (*Apc*$^{fl/fl}$; *Tp53*$^{R172H/+}$; WNT-activated model) consists of the combination of a floxed *Apc* allele and an inducible allele of *Tp53*^{R172H}. The third model (*Cdh1*$^{fl/fl}$; *Apc*$^{fl/fl}$; diffuse model) contains a floxed *Cdh1* allele (Cdh1^{tm2Kem}) and a floxed *Apc* allele (Apc^{tm2Rak}) (Boussadia *et al*, 2002; Kuraguchi *et al*, 2006). Organoids were generated from isolated corpus glands. To induce the specific mutations, established murine gastric organoids were infected with an Adeno-CMV-Cre-GFP virus (Gene Transfer Vector Core facility, Boston, USA) as previously described (Seidlitz *et al*, 2019). Organoids with an *Apc* mutation were selected by growing in WNT3A- and Rspondin-free medium. Organoids carrying a *Kras* mutation were selected in medium without EGF. Successful recombination and selection was confirmed by genotyping (Fig EV1). The organoid models were for all performed experiments cultivated in their specific selection media. Mouse experiments were approved by the local animal welfare commission (DD24-5131/367/18).

Genotyping of mouse organoids via PCR

Murine organoid DNA was isolated via phenol/chloroform extraction and isopropanol precipitation according to a standard protocol. PCR reactions were performed with the Hot Start Go Taq polymerase (Promega) using the following primers:

Kras^{G12D} (Kras G12D_3: CTAGCCACCATGGCTTGAGT; Kras G12D_4: ATGTCTTTCCCCAGCACAGT; Kras G12D_5: TCCGAATTC AGTGACTACAGATG).

Tp53^{R172H} (LSL_p53_for: AGCTAGCCACCATGGCTTGAGTAAGT CTGCA; WT_p53_for: CTGTTCGTTCCATTCCGTTT; WT_p53_rev: AGCCACACTGACAATAGGAGGT).

Apc (APC_fwd: GAGAAACCCTGTCTCGAAAAAA; APC_rev: AGT GCTGTTTCTATGAGTCAAC; APC_int14R4: TTGGCAGACTGTGTA TATAAGC).

Cdh1 (mCDH1_E10_f: ACTTTGGTGTGGGTCAGGAA; mCDH1_E10_r: GTGTCCCTCCAAATCCGATAC; mCDH1_I5_f: GCCTGTGAC ACATGAAGCAT).

Patient-derived organoid generation and cultivation

Patient-derived cancer organoids from stomach or esophago-gastric junction adenocarcinoma were generated and cultured according to an adapted protocol from Seidlitz *et al* (2019). Briefly, tumor or normal gastric tissue was cut into small pieces and washed with basal Advanced Dulbecco's modified Eagle medium (DMEM)/F12 (Gibco) supplemented with 1× primocin (Invitrogen), 1× glutamax (Thermo Fisher), and 10 mM HEPES (Thermo Fisher). The tumor tissue was digested at 37°C using 1 mg/ml dispase II (Roche) and 0.1 mg/ml collagenase XI (Sigma-Aldrich). Regular inversion was performed until small, floating tumor patches became visible.

Normal tissues pieces were incubated in chelating buffer (sterile distilled water with 5.6 mmol/l Na_2HPO_4, 8.0 mmol/l KH_2PO_4, 96.2 mmol/l NaCl, 1.6 mmol/l KCl, 43.4 mmol/l sucrose, 54.9 mmol/l D-sorbitol, 0.5 mmol/l DL-dithiothreitol, pH 7) supplemented with 10 mM EDTA for 15 min at room temperature. Stomach mucosa fragments were squeezed to release gastric glandular structures.

Normal and tumor tissue fragments were separated, washed, centrifuged (300 *g*, 5 min), resuspended in Matrigel (Corning), seeded in 20–30 μl droplets into 48-well plates and after solidification overlaid with human stomach medium supplemented with 10 μM ROCK inhibitor Y-27632 (Sigma-Aldrich). The study was approved by the ethical committee of the TU Dresden (EK76032013; EK451122014), and written consent was obtained from all patients. Experiments were performed in accordance with the statements of the Declaration of Helsinki and the Belmont Report from the U.S. Department of Health and Human Services.

Characterization of EGC PDOs for RTK/MAPK pathway alterations

For the analysis of mutations in the RTK/MAPK pathway, DNA sequencing data were obtained from different sequencing technologies. The analysis of DD107, DD109, DD191, and DD282 was described previously (Seidlitz *et al*, 2019). Whole genome sequencing of DD483, DD485, DD487, and DD536 was conducted accordingly. In addition, whole genome sequencing of DD472, DD540, and DD548 was also obtained in the same way, but no paired normal samples were analyzed allowing the identification of somatic mutations. DD826 and DD1028 were analyzed using the TruSight Oncology 500 Kit (Illumina) as described previously (Hennig *et al*, 2022). The criteria to classify RTK/MAPK-altered versus unaltered were chosen based on current knowledge from the literature on the impact of alteration on this pathway. EGFR and ERBB2 amplifications/gains and resulting overexpression are known to result in an activation of RTK and downstream MAPK signaling. As CNV gains alone do not necessarily result in high expressions, the overexpression based on bulk RNA sequencing data was analyzed in addition as previously described (Seidlitz *et al*, 2019) and EGFR or ERBB2 overexpressing organoids (> 3× fold change compared with normal organoids) were considered as RTK/MAPK-altered. Next to expression level changes, mutations in the EGFR family and of downstream signaling mediators such as RAS, RAF, and other MAP-kinases can lead to an activation of MAPK signaling. Organoids with a known pathogenic mutation in either EGFR, ERBB2, RAS, RAF, or MAPK were, therefore, considered RTK/MAPK-altered. Pathogenic mutations were identified using the cosmic database and given FATHMM (Functional Analysis Through Hidden Markov Models) prediction, with scores above 0.90 being considered pathogenic. Classification and decision-relevant alterations are summarized in Table EV1.

Cell cycle assay

Cell cycle phase was analyzed by propidium iodide (PI) intercalation. Organoids were seeded 48 h prior analysis in 48-well plates and covered with corresponding stomach medium. Single cells were prepared using TrypLE (Gibco) and fixed with 70% EtOH at 4°C. For the cell cycle analysis, 250,000 cells were used and prepared according to a standard protocol. Single cells were incubated with 0.1 mg RNase and 0.05 mg PI each at 37°C. Cells were analyzed using the LSRII (BD). Each organoid line was analyzed in three independent experiments. All values per dose were averaged and the standard deviation calculated. Graphs were generated with Prism. Statistical analysis was performed using the two-tailed Student's *t*-test: *< 0.05, **< 0.01.

 © 2022 The Authors

EdU proliferation assay

Proliferation was assessed by EdU incorporation. Here, organoids were seeded in 48-well plates 48 h prior measurement. Single cells were prepared by using TrypLE, and staining was performed with the Click-iT EdU Flow Cytometry Assay (Invitrogen). Cells were further analyzed using the LSRII. Each organoid line was analyzed in three independent experiments. All values per dose were averaged and the standard deviation calculated. Graphs were generated with Prism. Statistical analysis was performed using the two-tailed Student's *t*-test: *< 0.05, **< 0.01.

HE staining, IHC, and imaging

Murine organoid models, human tissues, and human PDOs were fixed with 4% paraformaldehyde, dehydrated and embedded in paraffin. Organoids were sectioned in 2.5 μm sections. HE staining was performed according to standard protocols. Organoid slides were stained in hematoxylin for 1 min and counterstained in eosin for 2 min. Tissue slides were stained for 5 min in hematoxylin and counterstained with eosin for 3 min. IHC was performed according to standard protocols using the following antibodies: TP53 (1:2,000; Leica, CM5), β-catenin (1:1,000; abcam, ab32572), E-cadherin (1:100; Cell Signaling Technology, #14472S, 4A2) and pERK1/2 (1:800; Cell Signaling Technology, #4376S), Periodic acid Schiff's reaction (PAS; Abcam, #150680). Antigen retrieval was performed with 0.01 M citrate buffer, pH6. The Signal Stain Detection Boost IHC/HRP rabbit (Cell Signaling Technology, #8144S) or mouse (Cell Signaling Technology, #8125S) was used for detection. Imaging was performed using an EVOS FL Auto (Life Technologies).

Drug library screening and viability measurement

For the drug library screening of murine organoids, each compound was diluted in DMSO and spotted into 384-well plates to achieve the desired final concentrations of 6.7, 13.3, 43.2, 123, 352.4, 997.5, 2992.5, 8,645 and 25,004 nM in a total volume of 60 μl. Murine organoids were mechanically dissociated, suspended in 15 μl matrigel, and seeded into pre-spotted 384-well plates. Organoids were covered with 45 μl of mouse stomach organoid medium. The organoids were incubated for 72 h, and viability was analyzed using Presto Blue Cell Viability Reagent (Invitrogen). The Presto Blue reagent (final 1×) was added, organoids were incubated for 3 h at 37°C, and fluorescence measured at 560/590 nm using the Varioskan Lux (Thermo Fisher Scientific). Conventional chemotherapeutics oxaliplatin (0.03, 0.1, 0.3, 1, 3, 10, 30, 100, and 300 μM), epirubicin (1, 3, 10, 30, 100, 300, and 1,000 nM), paclitaxel (0.1, 0.3, 1, 3, 10, 30, and 100 nM), docetaxel (0.1, 0.3, 1, 3, 10, 30, and 100 nM) as well as the targeted drugs nutlin-3a (0.1, 0.3, 1, 3, and 10 μM), the B-RAF inhibitors LY3009120 (0.03, 0.09, 0.3, 0.9, 3, 9, and 30 μM), TAK-632 (0.3, 0.9, 3, 9, and 30 μM), vemurafenib (0.1, 0.3, 1, 3, and 10 μM), and dabrafenib (0.1, 0.3, 1, 3, and 10 μM) were individually tested. To this aim, murine organoids were seeded, and drugs in the above-mentioned concentration range added to mouse organoid medium and incubated for 72–144 h at 37°C. Cell viability was determined by using the Presto Blue assay. Each organoid line was tested at three independent passages with two wells as technical replicates. All values per dose ($n = 6$) were averaged, and the standard deviation calculated. In our experience, this setup resulted in reproducible data and low standard deviations. Graphs were generated with Prism (GraphPad 8.4.0; GraphPad, La Jolla, CA, USA), and IC50 was determined with non-linear regression (*< 0.05).

Human PDOs were tested for their sensitivity to HDAC inhibitors. Panobinostat (1.76, 5.28, 15.8, 47.5, 142.5, 427.4, 1,282, and 3,846 nM), entinostat (0.23, 0.69, 2.06, 6.17, 18.5, 55.6, 167, and 500 μM), tacedinaline (0.23, 0.69, 2.06, 6.17, 18.5, 55.6, 167, and 500 μM), and vorinostat (0.1, 0.3, 0.91, 2.74, 8.23, 24.7, 74.1, and 222 μM) as well as the MEK1/2 inhibitor trametinib (0.61, 2.44, 9.77, 39, 156.2, 625, 2,500, and 10,000 nM). Here, PDOs were seeded, and drugs in the above-mentioned concentration range added into the human organoid medium and incubated for 144 h at 37°C. Treatment was refreshed after 72 h of incubation. Cell viability was determined by using the Presto Blue assay. Each organoid line was analyzed in three independent experiments. All values per dose were averaged, and the standard deviation calculated (Table EV2). Graphs were generated with Prism, and the area under the curve (AUC) was calculated. Z transformed AUC values were heatmapped, and associations were determined using Pearson correlation coefficient.

Liquid chromatography–tandem mass spectrometry (LC–MS/MS)

For proteomics, organoids were lysed using 7 M urea, 2 M thiourea, in 100 mM Hepes pH 7.5, supplemented with 1:1,000 (v/v) of benzonase (Novagen) and 1:100 (v/v) of Halt™ phosphatase and protease inhibitor cocktail 100× (Thermo Fisher Scientific). Protein concentration was determined using micro BCA (Thermo) using BSA as standard. 50 μg samples were digested by means of the standard FASP protocol. Proteins were reduced and alkylated (15 mM TCEP, 50 mM CAA, in 100 mM TEAB, 30 min in the dark, room temperature (RT)) and sequentially digested with Lys-C (Wako) (protein:enzyme ratio 1:50, overnight (ON) at RT) and trypsin (Promega) (protein:enzyme ratio 1:100, 6 h at 37°C). Resulting peptides (50 μg) were labeled in 0.5 M TEAB using iTRAQ® reagent 8-plex following the manufacturer's instructions. Samples were mixed in 1:1 ratios based on total peptide amount, which was determined from an aliquot by comparing overall signal intensities on a regular LC–MS/MS run. The mixture was finally desalted using a Sep-Pak C18 cartridge (Waters) and dried prior to high-pH reverse-phase HPLC pre-fractionation. Peptides were pre-fractionated offline by means of high-pH reverse-phase chromatography using an Ultimate 3000 HPLC system equipped with a sample collector. Peptides were dissolved in 100 μl of phase A (10 mM NH_4OH) and loaded onto a XBridge BEH130 C18 column (3.5 μm, 250 mm length, and 4.6 mm ID) (Waters). Phase B was 10 mM NH_4OH in 90% CH_3CN. The following gradient (flow rate of 500 μl/min) was used: 0–50 min 25% B, 50–54 min 60% B, 54–61 min 70% B. Fifty fractions were collected and concatenated into 15 fractions. Phosphopeptides were enriched using TiO_2 micro-columns. Briefly, iTRAQ-labeled peptides were resuspended in 6% TFA and 80% CH_3CN and incubated for 20 min with TiO_2 beads (10 μm particle size) (GL-Science) using a sample: TiO_2 ratio of 1:2. Prior to incubation, TiO_2 beads were pre-conditioned with a solution of 20 mg/ml DHB in 80% CH_3CN 6% TFA for 20 min. Then, beads were sequentially washed with 100 μl of 6% TFA and 10% CH_3CN, 100 μl of 6% TFA and 100 μl of 40% CH_3CN and 6% TFA and 60% CH_3CN. Finally, phosphopeptides were eluted first with 20 μl of 5% NH_4OH and then with 20 μl 5% NH_4OH in 10% CH_3CN in the same vial.

© 2022 The Authors

Phosphopeptides were further fractionated with high-pH reverse-phase micro-columns. Briefly, 45 µl of phase A (20 mM NH_4OH) was added to the sample. Five 16-gauge disks of C18 stage tip were used. Sample was loaded into the tips thrice, and the flow-through was collected to a vial. Next, 50 µl of phase A was loaded and collected in the same vial as the flow-through. Peptides were sequentially eluted increasing the percentage of buffer B (20 mM NH3 in CH3CN) (i.e., 4, 8, 12, 20, 60, and 80%). The 60 and 80% fractions were pooled together. Samples were resuspended in 22 µl of 5% FA for subsequent LC–MS/MS analysis. For RAS-activated and diffuse organoids, LC–MS/MS was done by coupling a nanoLC-Ultra 1D+ system (Eksigent) to an impact mass spectrometer (Bruker) via a Captivespray source (Bruker) supplemented with a nanoBooster operated at 0.2 bar/min with isopropanol as dopant. Peptides were loaded into a trap column (NS-MP-10 BioSphere C18 5 µm, 20 mm length, Nanoseparations) for 10 min at a flow rate of 2.5 µl/min in 0.1% FA. Then, peptides were transferred to an analytical column (ReproSil Pur C18-AQ 1.9 µm, 400 mm length, and 0.075 mm ID) and separated using a 120-min effective linear gradient (buffer A: 4% ACN, 0.1% FA; buffer B: 100% ACN, 0.1% FA) at a flow rate of 300 nl/min. The gradient used was as follows: 0–2 min 2% B, 3–164.5 min 3% B, 165–175 min 98% B, and 176–180 min 2% B. The peptides were electrosprayed (1.35 V) into the mass spectrometer with a heated capillary temperature of 180°C. The mass spectrometer was operated in a data-dependent mode, with an automatic switch between MS (80–1,600 m/z) and MS/MS (80–1,600 m/z) scans using a top 30 method (threshold signal ≥ 500-counts, z ≥ 2 and m/z ≥ 350). An active exclusion of 30s was used. The precursor intensities were re-evaluated in the MS scan (n) regarding their values in the previous MS scan ($n-1$). Any m/z intensity exceeding five times the measured value in the preceding MS scan was reconsidered for MS/MS. Peptides were isolated using a 2 Th window and fragmented using collision-induced dissociation (CID) with a collision energy of 23–56 eV as function of the m/z value. For WNT-activated organoids total protein analysis, LC–MS/MS was done by coupling an UltiMate 3000 RSLCnano LC system to a Q Exactive Plus mass spectrometer (Thermo Fisher Scientific). Five microliters of peptides were loaded into a trap column (Acclaim™ PepMap™ 100 C18 LC Columns 5 µm, 20 mm length) for 3 min at a flow rate of 10 µl/min in 0.1% FA. For total protein analysis, peptides were transferred to an EASY-Spray PepMap RSLC C18 column (Thermo) (2 µm, 75 µm × 50 cm) operated at 45°C and separated using a 150 min effective gradient (buffer A: 0.1% FA; buffer B: 100% ACN, 0.1% FA) at a flow rate of 250 nl/min. The gradient used was, from 4 to 6% of buffer B in 2.5 min, from 6 to 42.5% B in 155 min, plus 10 additional min at 98% B. Peptides were sprayed at 1.8 kV into the mass spectrometer via the EASY-Spray source, and the capillary temperature was set to 300°C. For iTRAQ-labeled samples, the mass spectrometer was operated in a data-dependent mode, with an automatic switch between MS and MS/MS scans using a top 15 method. (Intensity threshold ≥ 5.6e4, dynamic exclusion of 30s and excluding charges unassigned, +1 and ≥ +6). MS spectra were acquired from 375 to 1,500 m/z with a resolution of 70,000 (200 m/z). Ion peptides were isolated using a 1.4 Th window and fragmented using higher-energy collisional dissociation (HCD) with a normalized collision energy of 32. MS/MS spectra were acquired with a fixed first mass of 100 m/z and a resolution of 17,500 (200 m/z). The ion target values were 3e6 for MS (maximum IT of 25 ms) and 1e5 for MS/MS (maximum IT of 45 msec). For iTRAQ-labeled phosphopeptides analysis, samples were separated in the above-described system in an 86.5 min effective gradient from 6 to 42.5% of ACN in H2O containing 0.1% FA, at a flow rate of 250 nl/min. Samples were analyzed in a Q Exactive HF-X Orbitrap-MS. MS spectra were acquired from 350 to 1,400 m/z with a resolution of 60,000 (200 m/z). Ion peptides were isolated using a 1.0 Th window and fragmented using higher-energy collisional dissociation (HCD) with a normalized collision energy of 32. MS/MS spectra were acquired with a fixed first mass of 100 m/z and a resolution of 30,000 (200 m/z). The ion target values were 3e6 for MS (maximum IT of 25 ms) and 1e5 for MS/MS (maximum IT of 5 ms).

Raw files of the MS were processed with MaxQuant (v 1.6.10.43) using the standard settings against a mouse protein database (UniProtKB, 2018, 53,449 sequences) supplemented with contaminants. Reporter ion MS2-based quantification was enabled for iTRAQ 8-plex. Carbamidomethylation of cysteines was set as a fixed modification whereas oxidation of methionines, protein N-term acetylation, deamidation of NQ and, for phosphopeptide identification experiments, phosphorylation of serines, threnonines, and tyrosines, as variable modifications. Minimal peptide length was set

The paper explained

Problem

Gastric cancer ranks the fifth most common and third leading cause of cancer-related deaths worldwide. Patient-derived cancer organoids (PDOs) constitute a three-dimensional cell culture system with self-renewal and self-organization capability recapitulating many aspects of the parental tumor. They also retain the complex individual mutational landscape, carrying between a few hundred to several thousand mutations. Data generated in these PDOs can, therefore, often not be generalized, but need to be interpreted bearing in mind the singularity of the analyzed tumor. The Cancer Genome Atlas (TCGA) consortium developed a molecular classification system of gastric cancer by describing four different subtypes with characteristic mutations and associated deregulated pathways. Organoids with defined pathway alterations could overcome the limitations of PDOs.

Results

We generated three murine organoid models with a defined genetic makeup by activating frequently altered pathways: a RAS-activated (*Kras*^{G12D}, *Tp53*^{R172H}), a WNT-activated (*Apc*$^{fl/fl}$, *Tp53*^{R172H}), and a diffuse (*Cdh1*$^{fl/fl}$, *Apc*$^{fl/fl}$) model. These organoid models were characterized concerning their phenotype, proteome expression, and sensitivity to drug treatment. We observed different organoid morphologies as well as proliferation rates. All three models altered the expression of a significant fraction of their proteome, affecting multiple processes and functions. A divergent response pattern to classical chemotherapy and targeted small molecules was recognized in a drug screen. We analyzed in detail the response of RAS-activated organoids upon interference with the RTK/MAPK pathway at different levels, revealing a sensitivity on the level of B-RAF and MEK1/2, but no differential response on the level of ERK1/2. Furthermore, the RAS-activated organoids showed a significantly increased sensitivity to HDAC inhibition. To evaluate whether this correlation is translatable to human gastric cancer, we analyzed gastric cancer PDOs and could show similar to the murine organoid model a sensitivity of RTK/MAPK-altered PDOs to trametinib and HDAC inhibitors.

Impact

By using murine and human organoids with RTK/MAPK alterations, an association between MAPK pathway activity and susceptibility to HDAC inhibition was uncovered, delineating a novel treatment approach for RTK/MAPK pathway altered gastric cancer patients.

© 2022 The Authors

to seven amino acids and a maximum of two tryptic missed-cleavages were allowed. Results were filtered at 1% FDR (peptide and protein level). The "proteinGroups.txt" or the preprocessed "phospho(STY)sites.txt" file was loaded in Prostar (v1.14) (Wieczorek *et al*, 2017) using the intensity values for further statistical analysis. Briefly, proteins with less than eight valid values in at least one experimental condition were filtered out. A global normalization of log2-transformed intensities across samples was performed using the LOESS function. Differential analysis was done using the empirical Bayes statistics Limma. Proteins with a P-value < 0.05 and a log2 ratio > 1 or < -1 were defined as regulated. The FDR was estimated to be below 5% by Benjamini–Hochberg.

Data availability

The mass spectrometry proteomics data have been deposited to the ProteomeXchange Consortium via the PRIDE partner repository with the dataset identifier PXD022015.

Expanded View for this article is available online.

Acknowledgements
Funding was provided by the Deutsche Krebshilfe (#70113745) and the Horizon 2020 program of the European Union (EPIC-XS consortium, #823839). Open Access funding enabled and organized by Projekt DEAL.

Author contributions
Therese Seidlitz: Conceptualization; data curation; formal analysis; validation; investigation; visualization; methodology; writing – original draft. **Tim Schmäche:** Conceptualization; data curation; formal analysis; validation; investigation; visualization; methodology; writing – original draft. **Fernando García:** Data curation; formal analysis; investigation; visualization; methodology; writing – original draft. **Joon Ho Lee:** Data curation; formal analysis; visualization; methodology. **Nan Qin:** Data curation; formal analysis; visualization; methodology. **Susan Kochall:** Data curation; formal analysis; visualization; methodology. **Juliane Fohgrub:** Data curation; formal analysis; visualization; methodology. **David Pauck:** Formal analysis; funding acquisition; visualization; methodology. **Alexander Rothe:** Data curation; formal analysis; visualization; methodology. **Bon-Kyoung Koo:** Writing – review and editing. **Jürgen Weitz:** Writing – review and editing. **Marc Remke:** Supervision; writing – review and editing. **Javier Muñoz:** Conceptualization; supervision; funding acquisition; validation; investigation; writing – original draft; writing – review and editing. **Daniel E Stange:** Conceptualization; supervision; funding acquisition; validation; investigation; writing – original draft; project administration; writing – review and editing.

Disclosure and competing interests statement
The authors declare that they have no conflict of interest.

References

Bang Y-J, Van Cutsem E, Feyereislova A, Chung HC, Shen L, Sawaki A, Lordick F, Ohtsu A, Omuro Y, Satoh T *et al* (2010) Trastuzumab in combination with chemotherapy versus chemotherapy alone for treatment of HER2-positive advanced gastric or gastro-oesophageal junction cancer (ToGA): a phase 3, open-label, randomised controlled trial. *Lancet* 376: 687 – 697

Bartfeld S, Clevers H (2017) Stem cell-derived organoids and their application for medical research and patient treatment. *J Mol Med* 95: 729 – 738

Boussadia O, Kutsch S, Hierholzer A, Delmas V, Kemler R (2002) E-cadherin is a survival factor for the lactating mouse mammary gland. *Mech Dev* 115: 53 – 62

Bray F, Ferlay J, Soerjomataram I, Siegel RL, Torre LA, Jemal A (2018) Global cancer statistics 2018: GLOBOCAN estimates of incidence and mortality worldwide for 36 cancers in 185 countries. *CA Cancer J Clin* 68: 394 – 424

Carter CA, Giaccone G (2012) Treatment of nonsmall cell lung cancer: overcoming the resistance to epidermal growth factor receptor inhibitors. *Curr Opin Oncol* 24: 123 – 129

Castellano E, Downward J (2011) RAS interaction with PI3K: more than just another effector pathway. *Genes Cancer* 2: 261 – 274

Chen A, Beetham H, Black MA, Priya R, Telford BJ, Guest J, Wiggins GAR, Godwin TD, Yap AS, Guilford PJ (2014) E-cadherin loss alters cytoskeletal organization and adhesion in non-malignant breast cells but is insufficient to induce an epithelial-mesenchymal transition. *BMC Cancer* 14: 552

Cristescu R, Lee J, Nebozhyn M, Kim K-M, Ting JC, Wong SS, Liu J, Yue YG, Wang J, Yu K *et al* (2015) Molecular analysis of gastric cancer identifies subtypes associated with distinct clinical outcomes. *Nat Med* 21: 449 – 456

Dempke WCM, Heinemann V (2010) Ras mutational status is a biomarker for resistance to EGFR inhibitors in colorectal carcinoma. *Anticancer Res* 30: 4673 – 4677

Fatehullah A, Tan SH, Barker N (2016) Organoids as an *in vitro* model of human development and disease. *Nat Cell Biol* 18: 246 – 254

Ferlay J, Colombet M, Soerjomataram I, Mathers C, Parkin DM, Piñeros M, Znaor A, Bray F (2019) Estimating the global cancer incidence and mortality in 2018: GLOBOCAN sources and methods. *Int J Cancer* 144: 1941 – 1953

Gadducci A, Cosio S, Muraca S, Genazzani AR (2002) Molecular mechanisms of apoptosis and chemosensitivity to platinum and paclitaxel in ovarian cancer: biological data and clinical implications. *Eur J Gynaecol Oncol* 23: 390 – 396

Ge S, Xia X, Ding C, Zhen B, Zhou Q, Feng J, Yuan J, Chen R, Li Y, Ge Z *et al* (2018) A proteomic landscape of diffuse-type gastric cancer. *Nat Commun* 9: 1012

Georgakopoulos N, Prior N, Angres B, Mastrogiovanni G, Cagan A, Harrison D, Hindley CJ, Arnes-Benito R, Liau S-S, Curd A *et al* (2020) Long-term expansion, genomic stability and *in vivo* safety of adult human pancreas organoids. *BMC Dev Biol* 20: 4

Hatzivassiliou G, Song K, Yen I, Brandhuber BJ, Anderson DJ, Alvarado R, Ludlam MJC, Stokoe D, Gloor SL, Vigers G *et al* (2010) RAF inhibitors prime wild-type RAF to activate the MAPK pathway and enhance growth. *Nature* 464: 431 – 435

Hennig A, Baenke F, Klimova A, Drukewitz S, Jahnke B, Brückmann S, Secci R, Winter C, Schmäche T, Seidlitz T *et al* (2022) Detecting drug resistance in pancreatic cancer organoids guides optimized chemotherapy treatment. *J Pathol* 257: 607 – 619

Huch M, Gehart H, van Boxtel R, Hamer K, Blokzijl F, Verstegen MMA, Ellis E, van Wenum M, Fuchs SA, de Ligt J *et al* (2015) Long-term culture of genome-stable bipotent stem cells from adult human liver. *Cell* 160: 299 – 312

Hunt RH, Camilleri M, Crowe SE, El-Omar EM, Fox JG, Kuipers EJ, Malfertheiner P, McColl KEL, Pritchard DM, Rugge M *et al* (2015) The stomach in health and disease. *Gut* 64: 1650 – 1668

Jackson EL, Willis N, Mercer K, Bronson RT, Crowley D, Montoya R, Jacks T, Tuveson DA (2001) Analysis of lung tumor initiation and progression using conditional expression of oncogenic K-ras. *Genes Dev* 15: 3243 – 3248

Jänne PA, van den Heuvel MM, Barlesi F, Cobo M, Mazieres J, Crinò L, Orlov S, Blackhall F, Wolf J, Garrido P *et al* (2017) Selumetinib plus docetaxel compared with docetaxel alone and progression-free survival in patients with KRAS-mutant advanced non–small cell lung cancer: the SELECT-1 randomized clinical trial. *JAMA* 317: 1844 – 1853

Kuraguchi M, Wang XP, Bronson RT, Rothenberg R, Ohene-Baah NY, Lund JJ, Kucherlapati M, Maas RL, Kucherlapati R (2006) Adenomatous polyposis coli (APC) is required for normal development of skin and thymus. *PLoS Genet* 2: 1362 – 1374

Lauren P (1965) The two histological main types of gastric carcinoma: diffuse and so-called intestinal-type carcinoma. *Acta Pathol Microbiol Scand* 64: 31 – 49

Lei Z, Tan IB, Das K, Deng N, Zouridis H, Pattison S, Chua C, Feng Z, Guan YK, Ooi CH *et al* (2013) Identification of molecular subtypes of gastric cancer with different responses to PI3-kinase inhibitors and 5-fluorouracil. *Gastroenterology* 145: 554 – 565

Lin X, Howell SB (2006) DNA mismatch repair and p53 function are major determinants of the rate of development of cisplatin resistance. *Mol Cancer Ther* 5: 1239 – 1247

Matano M, Date S, Shimokawa M, Takano A, Fujii M, Ohta Y, Watanabe T, Kanai T, Sato T (2015) Modeling colorectal cancer using CRISPR-Cas9–mediated engineering of human intestinal organoids. *Nat Med* 21: 256 – 262

Merz V, Gaule M, Zecchetto C, Cavaliere A, Casalino S, Pesoni C, Contarelli S, Sabbadini F, Bertolini M, Mangiameli D *et al* (2021) Targeting KRAS: the elephant in the room of epithelial cancers. *Front Oncol* 11: 638360

Michael D, Oren M (2003) The p53–Mdm2 module and the ubiquitin system. *Semin Cancer Biol* 13: 49 – 58

Olive KP (2004) Mutant p53 gain of function in two mouse models of Li-Fraumeni syndrome. *Cell* 119: 847 – 860

Poulikakos PI, Zhang C, Bollag G, Shokat KM, Rosen N (2010) RAF inhibitors transactivate RAF dimers and ERK signalling in cells with wild-type BRAF. *Nature* 464: 427 – 430

Samson P, Lockhart AC (2017) Biologic therapy in esophageal and gastric malignancies: current therapies and future directions. *J Gastrointest Oncol* 8: 418 – 429

Sato T, Vries RG, Snippert HJ, van de Wetering M, Barker N, Stange DE, van Es JH, Abo A, Kujala P, Peters PJ *et al* (2009) Single Lgr5 stem cells build crypt-villus structures *in vitro* without a mesenchymal niche. *Nature* 459: 262 – 265

Seidlitz T, Merker SR, Rothe A, Zakrzewski F, von Neubeck C, Grützmann K, Sommer U, Schweitzer C, Schölch S, Uhlemann H *et al* (2019) Human gastric cancer modelling using organoids. *Gut* 68: 207 – 217

Smyth EC, Nilsson M, Grabsch HI, van Grieken NCT, Lordick F (2020) Gastric cancer. *Lancet* 396: 635 – 648

Stange DE, Koo BK, Huch M, Sibbel G, Basak O, Lyubimova A, Kujala P, Bartfeld S, Koster J, Geahlen JH *et al* (2013) Differentiated Troy+ chief cells act as reserve stem cells to generate all lineages of the stomach epithelium. *Cell* 155: 357 – 368

Suraweera A, O'Byrne KJ, Richard DJ (2018) Combination therapy with histone deacetylase inhibitors (HDACi) for the treatment of cancer: achieving the full therapeutic potential of HDACi. *Front Oncol* 8: 92

The Cancer Genome Atlas Research Network (2014) Comprehensive molecular characterization of gastric adenocarcinoma. *Nature* 513: 202 – 209

Toledo F, Wahl GM (2007) MDM2 and MDM4: p53 regulators as targets in anticancer therapy. *Int J Biochem Cell Biol* 39: 1476 – 1482

Tung M-C, Lin P-L, Wang Y-C, He T-Y, Lee M-C, Yeh S-D, Chen C-Y, Lee H (2015) Mutant p53 confers chemoresistance in non-small cell lung cancer by upregulating Nrf2. *Oncotarget* 6: 41692 – 41705

Van Cutsem E, Hidalgo M, Canon J-L, Macarulla T, Bazin I, Poddubskaya E, Manojlovic N, Radenkovic D, Verslype C, Raymond E *et al* (2018) Phase I/II trial of pimasertib plus gemcitabine in patients with metastatic pancreatic cancer. *Int J Cancer* 143: 2053 – 2064

Vlachogiannis G, Hedayat S, Vatsiou A, Jamin Y, Fernández-mateos J, Khan K, Lampis A, Eason K, Huntingford I, Burke R *et al* (2018) Patient-derived organoids model treatment response of metastatic gastrointestinal cancers. *Science* 359: 920 – 926

Wagle M-C, Kirouac D, Klijn C, Liu B, Mahajan S, Junttila M, Moffat J, Merchant M, Huw L, Wongchenko M *et al* (2018) A transcriptional MAPK Pathway Activity Score (MPAS) is a clinically relevant biomarker in multiple cancer types. *NPJ Precis Oncol* 2: 7

Wee S, Jagani Z, Xiang KX, Loo A, Dorsch M, Yao Y-M, Sellers WR, Lengauer C, Stegmeier F (2009) PI3K pathway activation mediates resistance to MEK inhibitors in KRAS mutant cancers. *Cancer Res* 69: 4286 – 4293

Wensink GE, Elias SG, Mullenders J, Koopman M, Boj SF, Kranenburg OW, Roodhart JML (2021) Patient-derived organoids as a predictive biomarker for treatment response in cancer patients. *NPJ Precis Oncol* 5: 30

Wieczorek S, Combes F, Lazar C, Giai Gianetto Q, Gatto L, Dorffer A, Hesse A-M, Couté Y, Ferro M, Bruley C *et al* (2017) DAPAR & ProStaR: software to perform statistical analyses in quantitative discovery proteomics. *Bioinformatics* 33: 135 – 136

Yamada T, Amann JM, Tanimoto A, Taniguchi H, Shukuya T, Timmers C, Yano S, Shilo K, Carbone DP (2018) Histone deacetylase inhibition enhances the antitumor activity of a MEK inhibitor in lung cancer cells harboring RAS mutations. *Mol Cancer Ther* 17: 17 – 25

Zhao B, Wang L, Qiu H, Zhang M, Sun L, Peng P, Yu Q, Yuan X (2017) Mechanisms of resistance to anti-EGFR therapy in colorectal cancer. *Oncotarget* 8: 3980 – 4000

License: This is an open access article under the terms of the Creative Commons Attribution License, which permits use, distribution and reproduction in any medium, provided the original work is properly cited.

 © 2022 The Authors

Expanded View Figures

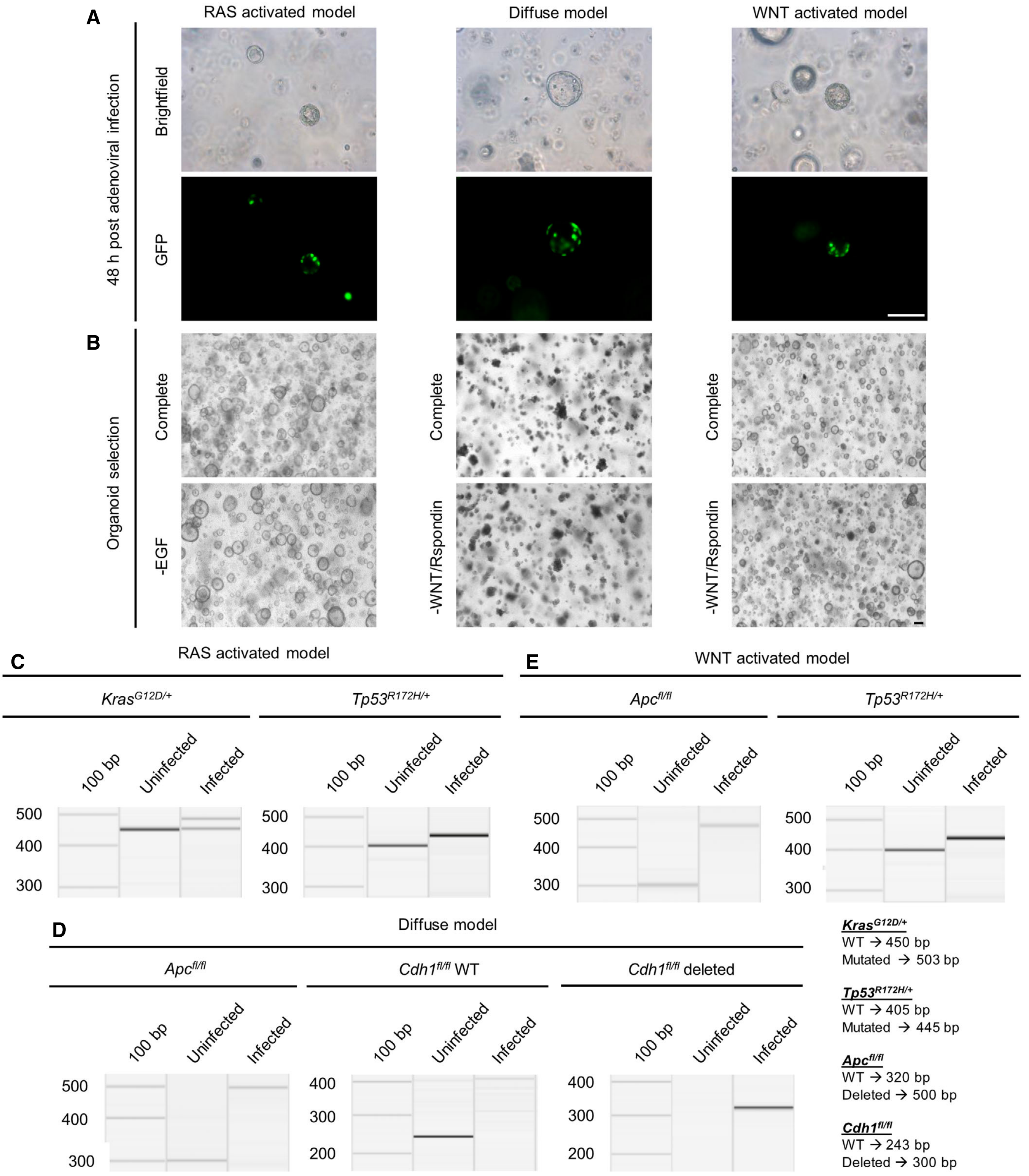

Figure EV1.

◀

Figure EV1. Generation of organoid models.

A Adenoviral infection of organoids with a Cre-GFP expressing recombinase. Fluorescence microscopy 24-h post-infection (scale bar 100 μm).

B Selection of organoids based on altered pathway. The RAS-activated model was selected via EGF removal from the normal cultivation medium. The diffuse and WNT-activated models were enriched by depletion of WNT3A and Rspondin (scale bar 25 μm).

C–E Genotyping PCRs of infected and selected organoid models to document successful recombination. *Tp53*$^{R172H/+}$ in the WNT-activated organoids showed loss of heterozygosity of the wild-type allele after activating the R172H mutation.

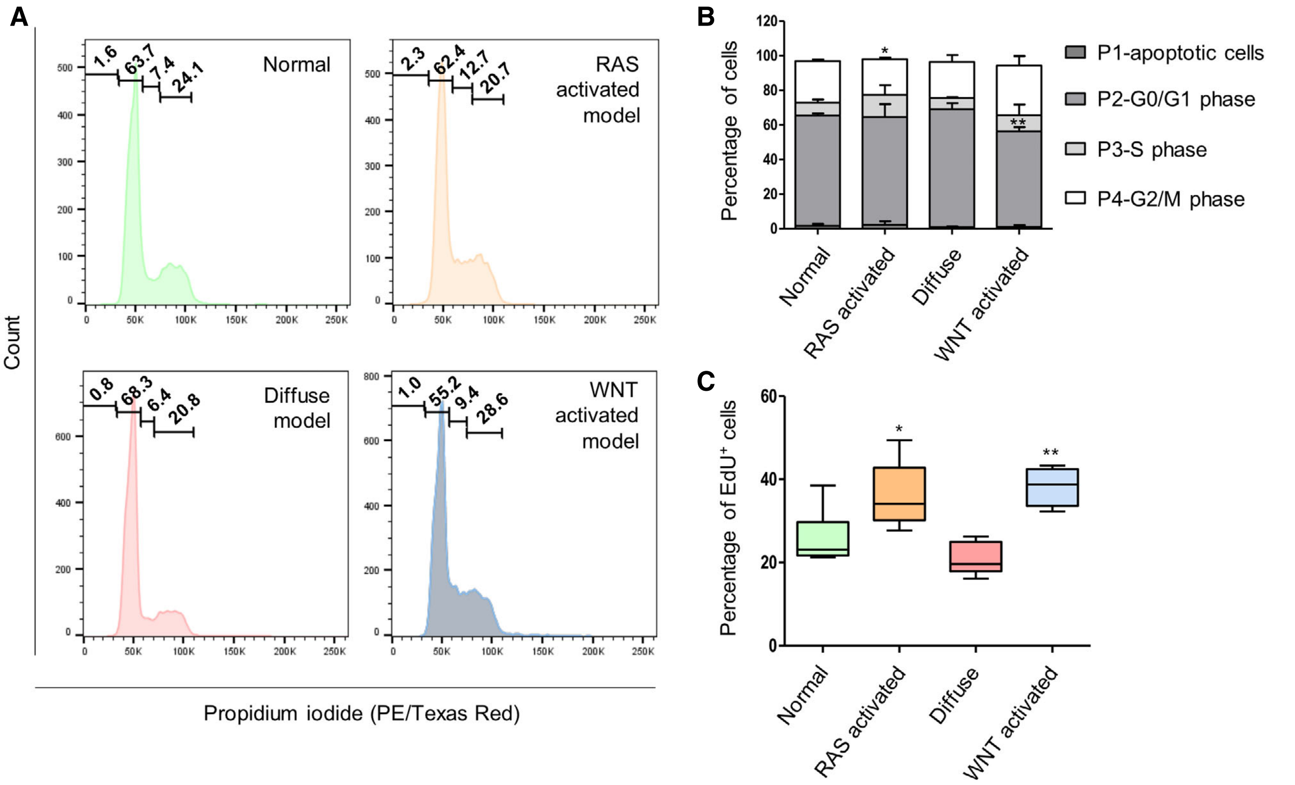

Figure EV2. Proliferative capacity of organoid models.

A Exemplary cell cycle analysis of normal organoids and organoid models.

B Quantitative representation of cell cycle analysis (two-tailed Student's *t*-test model vs. normal; *< 0.05; **< 0.01; RAS-activated P4 G2/M phase $P = 0.0105$, WNT-activated P2 G0/G1 phase $P = 0.0061$, biological replicate $n = 3$, data are shown as mean ± SD).

C Proliferation rate of organoids assessed by EdU proliferation assays. Two-tailed Student's *t*-test organoid models versus normal stomach organoids (*< 0.05; **< 0.01; RAS-activated $P = 0.031$; WNT-activated $P = 0.0017$, biological replicates $n = 3$, data are shown as mean ± SD).

 © 2022 The Authors

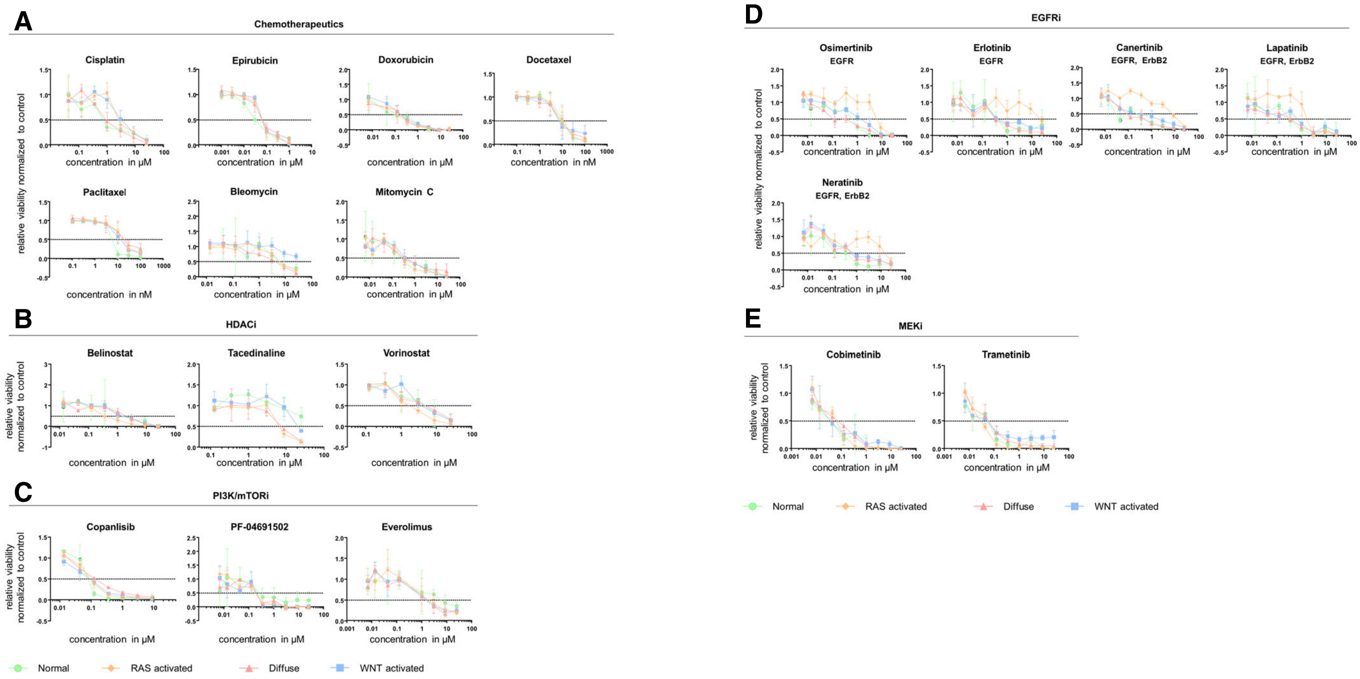

Figure EV3. Dose–response curves of normal and organoid models upon treatment with classical chemotherapeutics and targeted therapies.

A–E Drug response curves upon treatment with (A) classical chemotherapeutics (cisplatin, epirubicin, doxorubicin, docetaxel, paclitaxel, bleomycin, and mitomycin C), (B) HDAC inhibitors (belinostat, tacedenaline, and vorinostat), (C) PI3K/mTOR inhibitors (copanlisib, PF-04691502, and everolimus), (D) EGFR inhibitors (osimertinib, erlotinib, canertinib, lapatinib, and neratinib) and (E) MEK1/2 inhibitors (cobimetinib and trametinib) (biological replicates $n = 3$, data are shown as mean $\pm$ SD).

© 2022 The Authors

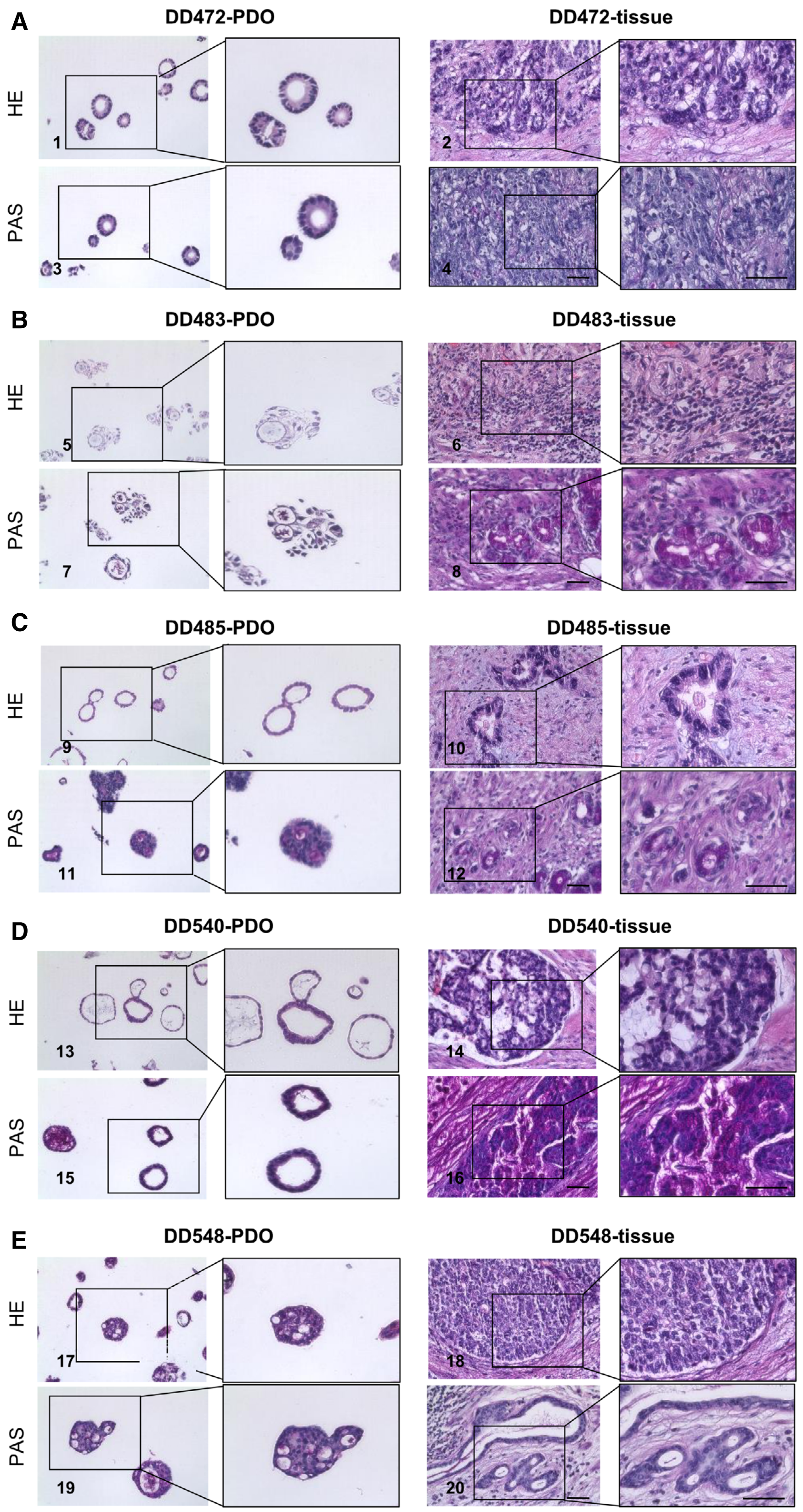

Figure EV4. Histological comparison of PDOs to corresponding primary tissue.

A HE staining (1 and 2) and PAS staining (3 and 4) of DD472 PDO and primary tissue (scale bar 50 µm).

B HE staining (5 and 6) and PAS staining (7 and 8) of DD483 PDO and primary tissue (scale bar 50 µm).

C HE staining (9 and 10) and PAS staining (11 and 12) of DD485 PDO and primary tissue (scale bar 50 µm).

D HE staining (13 and 14) and PAS staining (15 and 16) of DD540 PDO and primary tissue (scale bar 50 µm).

E HE staining (17 and 18) and PAS staining (19 and 20) of DD548 PDO and primary tissue (scale bar 50 µm).

© 2022 The Authors

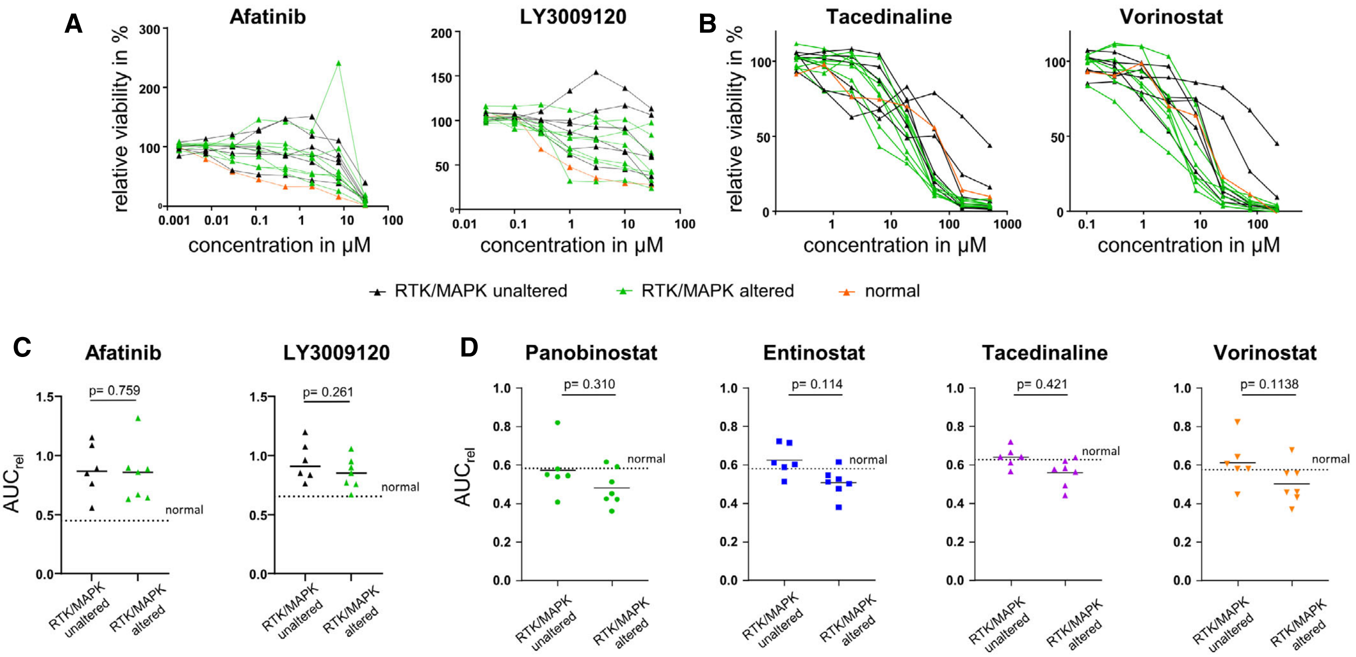

Figure EV5. EGFR, B-RAF, and HDAC inhibition of RTK/MAPK-altered versus non-altered gastric cancer PDOs.

A Drug response curves upon EGFR inhibition with afatinib or B-RAF with LY3009120 (biological replicates n = 3, data are presented as mean, SD values are shown in Table EV2).

B Drug response curves upon HDAC inhibition with tacedinaline and vorinostat (biological replicates n = 3, data are presented as mean, SD values are shown in Table EV2).

C Comparison of the relative area under the curve (AUC_{rel}) of afatinib and LY3009120-treated RTK/MAPK-altered (biological replicates n = 7) versus RTK/MAPK-unaltered human PDOs (biological replicates n = 6, two-tailed Student's t-test). The dashed line "normal" represents the AUC_{rel} of normal gastric PDOs as a reference parameter. (two-tailed Student's t-test).

D Comparison of the relative area under the curve (AUC_{rel}) of panobinostat, entinostat, tacedinaline, and vorinostat-treated RTK/MAPK-altered (biological replicates n = 7, two-tailed Student's t-test) versus RTK/MAPK-unaltered human PDOs (biological replicates n = 6). The dashed line "normal" represents the AUC_{rel} of normal gastric PDOs as a reference parameter.

© 2022 The Authors
