# Supplementary material for: Sensitivity towards HDAC inhibition is associated with RTK/MAPK pathway activation in gastric cancer
Source: EMBO Mol Med. 2022 Aug 22;14(10):e15705. doi: 10.15252/emmm.202215705 (PMC9549728; doi:10.15252/emmm.202215705)
Supplement: Supplementary file 9 [file EMMM-14-e15705-s006.pdf]

EMBO Molecular Medicine

Sensitivity towards HDAC inhibition is associated with RTK/MAPK pathway activation in gastric cancer

Therese Seidlitz, Tim Schmäche, Fernando Garcia, Joon Ho Lee, Nan Qin, Susan Kochall, Juliane Fohgrub, David Pauck, Alexander Rothe, Bon-Kyoung Koo, Jürgen Weitz, Marc Remke, Javier Munoz, and Daniel Stange
DOI: 10.15252/emmm.202215705

Corresponding author: Daniel Stange (daniel.stange@ukdd.de)

Review Timeline:

Submission Date:	19th Jan 22
Editorial Decision:	8th Mar 22
Revision Received:	28th Jun 22
Editorial Decision:	25th Jul 22
Revision Received:	1st Aug 22
Accepted:	2nd Aug 22

Editor: Jingyi Hou

Transaction Report:

(Note: With the exception of the correction of typographical or spelling errors that could be a source of ambiguity, letters and reports are not edited. Depending on transfer agreements, referee reports obtained elsewhere may or may not be included in this compilation. Referee reports are anonymous unless the Referee chooses to sign their reports.)

8th Mar 2022

Dear Dr. Stange,

Thank you again for submitting your work to EMBO Molecular Medicine. We have now heard back from the three referees who agreed to evaluate your manuscript. As you will see from the reports below, the referees acknowledge the potential interest of the study. Still, they raise a series of concerns, which we would ask you to address in a revision of the manuscript.

I think the referees' recommendations are relatively straightforward, so there is no need to reiterate their comments. All issues raised by the referees need to be satisfactorily addressed. Please feel free to contact me in case you would like to discuss in further detail any of the issues raised by the referees.

We would welcome the submission of a revised version within three months for further consideration. Please note that EMBO Molecular Medicine strongly supports a single round of revision. As acceptance or rejection of the manuscript will depend on another round of review, your responses should be as complete as possible.

EMBO Molecular Medicine has a "scooping protection" policy, whereby similar findings that are published by others during review or revision are not a criterion for rejection. Should you decide to submit a revised version, I do ask that you get in touch after three months if you have not completed it to update us on the status.

We are aware that many laboratories cannot function at full efficiency during the current COVID-19/SARS-CoV-2 pandemic and have therefore extended our "scooping protection policy" to cover the period required for a full revision to address the experimental issues. Please let me know should you need additional time, and also if you see a paper with related content published elsewhere.

Please read below for important editorial formatting and consult our author's guidelines for proper formatting of your revised article for EMBO Molecular Medicine.

I look forward to receiving your revised manuscript.

Use this link to login to the manuscript system and submit your revision: Link Not Available

Sincerely,
Jingyi

Jingyi Hou
Editor
EMBO Molecular Medicine

*****

When submitting your revised manuscript, please carefully review the instructions that follow below. We perform an initial quality control of all revised manuscripts before re-review; failure to include requested items will delay the evaluation of your revision.

We require:

1) A .docx formatted version of the manuscript text (including legends for main figures, EV figures and tables). Please make sure that the changes are highlighted to be clearly visible.

2) Individual production quality figure files as .eps, .tif, .jpg (one file per figure). For guidance, download the 'Figure Guide PDF': (https://www.embopress.org/page/journal/17574684/authorguide#figureformat).

3) A .docx formatted letter INCLUDING the reviewers' reports and your detailed point-by-point responses to their comments. As part of the EMBO Press transparent editorial process, the point-by-point response is part of the Review Process File (RPF), which will be published alongside your paper.

4) A complete author checklist, which you can download from our author guidelines (https://www.embopress.org/page/journal/17574684/authorguide#submissionofrevisions). Please insert information in the checklist that is also reflected in the manuscript. The completed author checklist will also be part of the RPF.

5) Please note that all corresponding authors are required to supply an ORCID ID for their name upon submission of a revised manuscript.

6) It is mandatory to include a 'Data Availability' section after the Materials and Methods. Before submitting your revision, primary datasets produced in this study need to be deposited in an appropriate public database, and the accession numbers and database listed under 'Data Availability'. Please remember to provide a reviewer password if the datasets are not yet public (see https://www.embopress.org/page/journal/17574684/authorguide#dataavailability).

In case you have no data that requires deposition in a public database, please state so in this section. Note that the Data Availability Section is restricted to new primary data that are part of this study.

7) For data quantification: please specify the name of the statistical test used to generate error bars and P values, the number (n) of independent experiments (specify technical or biological replicates) underlying each data point and the test used to calculate p-values in each figure legend. The figure legends should contain a basic description of n, P and the test applied. Graphs must include a description of the bars and the error bars (s.d., s.e.m.). See also 'Figure Legend' guidelines: https://www.embopress.org/page/journal/17574684/authorguide#figureformat

8) We would also encourage you to include the source data for figure panels that show essential data. Numerical data should be provided as individual .xls or .csv files (including a tab describing the data). For blots or microscopy, uncropped images should be submitted (using a zip archive if multiple images need to be supplied for one panel). Additional information on source data and instruction on how to label the files are available at

.

9) Our journal encourages inclusion of *data citations in the reference list* to directly cite datasets that were re-used and obtained from public databases. Data citations in the article text are distinct from normal bibliographical citations and should directly link to the database records from which the data can be accessed. In the main text, data citations are formatted as follows: "Data ref: Smith et al, 2001" or "Data ref: NCBI Sequence Read Archive PRJNA342805, 2017". In the Reference list, data citations must be labeled with "[DATASET]". A data reference must provide the database name, accession number/identifiers and a resolvable link to the landing page from which the data can be accessed at the end of the reference. Further instructions are available at .

10) We replaced Supplementary Information with Expanded View (EV) Figures and Tables that are collapsible/expandable online. A maximum of 5 EV Figures can be typeset. EV Figures should be cited as 'Figure EV1, Figure EV2" etc... in the text and their respective legends should be included in the main text after the legends of regular figures.

- For the figures that you do NOT wish to display as Expanded View figures, they should be bundled together with their legends in a single PDF file called *Appendix*, which should start with a short Table of Content. Appendix figures should be referred to in the main text as: "Appendix Figure S1, Appendix Figure S2" etc.

- Additional Tables/Datasets should be labeled and referred to as Table EV1, Dataset EV1, etc. Legends have to be provided in a separate tab in case of .xls files. Alternatively, the legend can be supplied as a separate text file (README) and zipped together with the Table/Dataset file.

See detailed instructions here:

.

11) The paper explained: EMBO Molecular Medicine articles are accompanied by a summary of the articles to emphasize the major findings in the paper and their medical implications for the non-specialist reader. Please provide a draft summary of your article highlighting

- the medical issue you are addressing,

- the results obtained and

- their clinical impact.

This may be edited to ensure that readers understand the significance and context of the research. Please refer to any of our published articles for an example.

12) For more information: There is space at the end of each article to list relevant web links for further consultation by our

readers. Could you identify some relevant ones and provide such information as well? Some examples are patient associations, relevant databases, OMIM/proteins/genes links, author's websites, etc...

13) Author contributions: the contribution of every author must be detailed in a separate section (before the acknowledgments).

14) A Conflict of Interest statement should be provided in the main text.

15) Every published paper now includes a 'Synopsis' to further enhance discoverability. Synopses are displayed on the journal webpage and are freely accessible to all readers. They include a short stand first (maximum of 300 characters, including space) as well as 2-5 one-sentences bullet points that summarizes the paper. Please write the bullet points to summarize the key NEW findings. They should be designed to be complementary to the abstract - i.e. not repeat the same text. We encourage inclusion of key acronyms and quantitative information (maximum of 30 words / bullet point). Please use the passive voice. Please attach these in a separate file or send them by email, we will incorporate them accordingly.

Please also suggest a striking image or visual abstract to illustrate your article as a PNG file 550 px wide x 300-600 px high.

EMBO Molecular Medicine has a "scooping protection" policy, whereby similar findings that are published by others during review or revision are not a criterion for rejection. Should you decide to submit a revised version, I do ask that you get in touch after three months if you have not completed it, to update us on the status.

***** Reviewer's comments *****

Referee #1 (Comments on Novelty/Model System for Author):

The study is original as it sistematically models different molecular types of gastric cancer using organoids with different mutations. in addition drugs are tested and sensitivity to drugs is shown to be different when organoids have different mutations which is relevant from the medical standpoint.

Referee #1 (Remarks for Author):

The study by Seidlitz et al sistematically models different molecular types of gastric cancer using organoids with different mutations. In addition drugs are tested and sensitivity to drugs is shown to be different when organoids have different mutations. The study is original and relevant. The study is very well performed and overall the conclusions are adequate to the results obtained. It is well written although english deserves reviewing for small mistakes.

Referee #2 (Comments on Novelty/Model System for Author):

The current study is mainly based on one mouse organoid per experiment, and validated by a series of human gastric cancer organoids. The data presentation is quite confusing and the major classifier between the RTK altered and unaltered group is not very clear, and the resultant trend is not strong either. Overall the paper needs much more work to substantiate the result.

Referee #2 (Remarks for Author):

Seidlitz et all used 3 murine organoid lines to generate gastric organoid models carrying Kras/Tp53; Apc/Tp53 and Cdh1/Apc mutations/inactivations; aiming to re-capitulate the RTK/MAPK, Wnt and diffuse pathways. They used these three organoid models for proteomic analysis and large scale drug screening, covering 196 drugs. The authors observed that the RAS cancer model is sensitive to HDAC inhibition. They then used 14 human gastric cancer organoids to validate the findings and concluded that there is a novel treatment approach for RTK/MAPK pathway altered gastric cancer.
Key issues:
The manuscript could be improved by providing more detail and clarity on the methods and results as detailed below:
1. What does three experiments mean, is it triplicate wells for each assay, or is it single well for each assay, but 3 biological replicates done on different passage? For the viability plot presented in in figure 3-5 with or without error bar, are they based on viability assay from triplicate wells of one experiment, or an average of three biological replicates but one well per experiment?
2. Table EV4. How are the molecular background of the human gastric cancer organoids determined? The criteria to classify the RTK/MAPK altered versus non-altered are not clear. Is the green text denoting those that were considered altered? Should there be difference between gain and amplification? How is oe (?over-expression) detected, is it by transcriptome or other means? If by transcriptome, what is the cut-off? The un-altered group also has HRAS CNV gain (e.g. DD191_2). How to distinguish path (green color text) from ?non-pathogenic mutation. Are all mutation confirmed to be somatic (i.e. subtracted from paired normal)? The unaltered group seems to have some cases with RTK member with oe also.
3. Two worksheets were provided for table EV3. The column heading of Normal, RAS activated, Diffuse and WNT activated for

the two worksheets do not have same sequence of arrangement and there seems to be a frameshift in the data. The log10IC50 provided in the table also do not seem to be on the same scale as those used in Fig EV4. For example, for the drug Belinostat, the Log10IC50 for the ras-activated group is 2.61 in the first worksheet of Table EV3, 3.2 in the 2nd worksheet of Table EV3. Based on figure EV4B, the dose for IC 50 is between 0.1 to 1 nM (it is not sure whether the log10IC50 is based on nM or microM as Figure EV4B has some drug plotted in nM and some in uM scale); which in log10 uM range should be between -3 to -4. However, the number provided in table EV3 seems to be in a different scale.
Other specific comments:
The supplementary tables should provide table title and legend for clarity.
The authors presented results for Trametinib in Fig 5 (for human gastric cancer organoids), but not in Fig. 4. It would be better to show similar drug response result in Fig. 4 as well to see if they are concordant.
Fig. 5G. The authors presented aggregated data for each HDACi for statistical comparison between the two groups, and showed a significant difference, but the difference for the individual HDACi does not reach statistical significance (Fig. EV5B). Thus the trend seems to be weak.

The manuscript could be improved by better integration of the proteomic part with the drug screening data, e.g. is there any relationship between discoveries from the proteomic profiling with the drug response data?

Referee #3 (Comments on Novelty/Model System for Author):

The authors here generate 3 genetic murine GC organoid subtypes that mimic aspects of the human TCGA subtypes. They perform unique proteomic characterization and drug/compound screening of these organoids. Certain aspects of their findings are recapitulated in actual human PDOs. Key findings are differential RTK/MAPK pathway inhibition in the subtypes and a sensitivity of the RAS activated model to HDAC inhibition. These organoids models are technical and may represent certain key aspects of the human molecular subtypes. However, these organoid models lack a tumor microenvironment and none of these models were explored in vivo. Given the importance and controversies of immunotherapy/anti-PDL1 blockade in GC, this is still a major area that needs to be studied.

Referee #3 (Remarks for Author):

Gastric cancer remains a difficult cancer to treat. Molecular (genomic) characterization efforts led by the TCGA have not yet translated to clinically meaningful improvements in diagnosis or treatment. In this report by Seidlitz et al., they expand their previous efforts at molecularly recapitulating the human molecular subtypes of gastric cancer with the generation of a WNT activated mouse organoid model. The authors then perform proteomic analysis and a 196 drug/compound screen to elucidate further molecular signatures and treatment sensitivities. Findings from these in vitro murine models were explored in human PDOs resulting in the key new finding that gastric cancers with RTK/MAPK pathway activation may be susceptible to HDAC inhibitors.

Major Comments:

1. The authors mention molecular classification based on TCGA studies. They should include mention of the other molecular classification studies including the Singapore-Duke and ACRG studies (PMID 23684942 and 25894828). It would also benefit the authors to make mention (Introduction or Discussion) that these detailed gastric cancer molecular studies have not translated to new improved GC diagnoses or treatments. In addition, these (TCGA) molecular classifications have had limited clinical applicability and are not readily used for any clinical decision making.

2. For the current manuscript, the authors use certain alleles to generate three subtypes. However, in their previous publication (PMID 29703791), Seidlitz et al., used slightly different alleles to generate the corresponding in vivo mouse models. Why have the authors chosen to use differing alleles for these murine organoid models? For example, previous CIN mouse model had KRAS, TP53, and SMAD4 alleles; and current model adopts only KRAS and TP53 alleles. And previous GS models used CDH1, APC, and KRAS alleles and CDH1, KRAS, and SMAD4 alleles; and current model adopts only the CDH1 and APC alleles. Was this done for simplicity or are there fundamental differences between these 3 allele and 2 allele models? In the human disease, mutations are accumulated gradually over many years. How does concomitant activation/inactivation of alleles recapitulate the molecular pathogenesis seen in the human disease?

3. One of the main strengths of this manuscript is the generation of the third molecular GC model. The authors mention that for the MSI TCGA subtype 78% of cases have WNT pathway alterations and 77% of cases have TP53 alterations. Can the authors provide a reference for these numbers. It is inferred by the reviewer that the authors despite naming the model WNT activated are modeling the MSI TCGA subtype. If this is the case, then why have the authors not considered deleting MMR proteins as an allelic combination? In addition, anti PDL-1 agents have become the backbone of treatment for MSI-high tumors (agnostic of tissue of origin) based on PMID 28596308. Assessing this would require an in vivo model (with intact tumor microenvironment) which is beyond the scope of this study. But the authors should at least comment and discuss this as a future direction of investigation.

4. In Fig EV1 C and D for the Tp53R172H/+ lanes, why is there only one band in the infected lanes for the RAS activated and WNT activated models? Given that this is a heterozygous allele, shouldn't 2 bands be seen similar to the KRAS G12D/+ experiments?

5. The authors only briefly characterize the organoid models in Figure 1A. They should consider moving some/all the data from Fig EV2 to Fig 1A as these are important stains that characterize and differentiate the various organoid models.

6. To clarify, the results obtained in EV2 were performed on organoids after selection and grown in no EGF (for the RAS activated model), and no WNT/Rspondin (for the Diffuse and WNT activated models).

7. At the end of the first page of the Results section, the authors mention that the WNT activated model has a "rather small organoid size", however in Fig EV2 C the WNT activated model H&E looks larger compared to the WT. Perhaps chose a more representative picture.

8. Can the authors comment on why the diffuse model had the least number of proliferating cells? Could this be due to culturing these less adherent cells in ECM/Matrigel? Would these cells grow better/proliferate in non adherent growth conditions?

9. Proteomic analysis of these different organoid subtypes is a unique approach performed by the authors. Are the authors able to show "positive controls" or expected increased proteomic pathways such as RAS pathway for the RAS activated model and WNT regulation for the WNT activated model? In addition, how do the proteomic profiles of these genetically engineered murine organoid models compare to the human subtypes (ie PMID 29520031). Further comment on this point would be warranted in the discussion section.

10. For the diffuse subtype, why would these organoids display increased adherent junction assembly as a proteomic signature with loss of CDH1? They also look very loosely adherent based on the images the authors show.

11. The authors should be commended for performing a large drug library screen using their organoid models. They document interesting disparate sensitivities of each line to chemotherapy and targeted therapies. Again, this data begs the question on how the sensitivity profiles of these murine organoid models compare to the human subtypes (ie PMID 28747339). In addition, can the authors comment on how these particular targeted pathways were selected and other pathways such as VEGF (with known drugs approved for GC such as ramucirumab not assessed)?

12. The authors found that PI3K/mTOR inhibitors did not result in differential responses between the models and normal organoids. Based on TCGA data, this pathway was more selectively mutated for the EBV and MSI subgroups, 80 and 42% respectively. Did the authors find proteomic evidence of PI3K/mTOR pathway activation?

13. The authors demonstrate variable response of the murine organoids to EGFR/MAPK pathway inhibition. The reviewer believes that demonstrating the activity of this pathway by immunoblot assay or IHC would strengthen the data and possibly lead to explanations as to the variable responses seen. Extension of these studies could also be considered for the human PDO data again to explain the difficulty in predicting a response based on the presence of certain pathway alterations.

14. The authors next generate a number of human GC PDOs. What are the demographics for these patients (ie sex, stage, age) and how specifically were the samples obtained via biopsy or surgical resection? In addition, some correlation between PDO and primary tumor should be shown as was demonstrated in the authors previous publication. Using these PDOs, the authors assess for RTK/MAPK pathway alterations, would it be possible for the authors to do a more expansive genomic mutational assessment and actually attempt to subgroup these PDOs into the TCGA subtypes.

15. In the discussion, the authors state that targeted therapies are thought to at least accompany, if not replace classical chemotherapy for GC treatment. Can they provide citations? No single agent targeted therapies have been approved for GC in the frontline setting PMID 28736629. In addition, the authors state that therapeutic targeting of the ERBB pathway represents a promising goal for GC. HER2 targeting agents in combination with chemotherapy is standard of care now for metastatic GC based on the ToGA study that the authors already reference.

16. The authors demonstrate that the PDOs have different sensitivities to MEK1/2 inhibition in Fig5D. What about EGFR and BRAF inhibition?

17. Can the authors comment on a particular mechanism or hypothesize about a mechanism as to why RTK/MAPK activated GCs have sensitivity to HDAC inhibition? Would this be a generalizable mechanism for other cancers?

Minor Comments:

1. The syntax of the sentence at the end of the first paragraph of the Introduction starting "Due to their highly individual pattern..." is difficult to understand. The authors should consider rewriting.

2. In the second paragraph of results, the authors should clarify that the Normal gastric corpus organoids are Normal Murine gastric corpus organoids.

3. In the sentence "The CDH1 loss in the diffuse organoid model...", "let as reported" is an unclear phrase.

4. For Fig EV3B, would please keep the location and position of the stars consistent and ideally above the bar graphs. Would also define PI for the axis in A and apcp in B. Also, the reviewer may have missed this but no mention of the methodology for the cell cycle assay was mentioned in the M&M section.

5. Proteomic GSEA analysis methodology should also be mentioned and detailed in the M&M section.

6. Page 2 of the results last sentence starting "The elevated RTK/RAS..." "let to translational activation" is an unclear phrase.

7.Page 5 of the results, sentence starting "within the cancer PDOS, a significantly higher sensitivity of PDOS with altered vs unaltered RTK/MAPK pathway could be observed, it is unclear sensitivity to what drug?

8. Fig EV5B, what does the "normal" and the dotted line for this graph represent?

9. Fig 5 E, please indicated what the green and black X axis coloring represent.

10. Page 2 of the Discussion, the phrase "Even so classical chemotherapy..." is unclear.

Dear Jingyi Hou,

We very much appreciated your and the reviewer's valuable comments concerning our manuscript 'Sensitivity towards HDAC inhibition is associated with RTK/MAPK pathway activation in gastric cancer' (EMM 2022-15705). Please find below a point-by-point response to the reviewers' comments. The revised manuscript includes additional data addressing the questions raised by the reviewers, which we think strengthened the manuscript substantially. Figures and text were modified accordingly, with the changes in the text in red colour.

Comments from Reviewer 1:

Referee #1 (Remarks for Author): The study is original and relevant. The study is very well performed and overall the conclusions are adequate to the results obtained. It is well written although English deserves reviewing for small mistakes.

Thank you for the positive comments. The revised manuscript has been revised by a native English speaker and we hope that errors have been corrected.

Comments from Reviewer 2:

Referee #2 (Remarks for Author): Seidlitz et all used 3 murine organoid lines to generate gastric organoid models carrying Kras/Tp53; Apc/Tp53 and Cdh1/Apc mutations/inactivations; aiming to re-capitulate the RTK/MAPK, Wnt and diffuse pathways. They used these three organoid models for proteomic analysis and large scale drug screening, covering 196 drugs. The authors observed that the RAS cancer model is sensitive to HDAC inhibition. They then used 14 human gastric cancer organoids to validate the findings and concluded that there is a novel treatment approach for RTK/MAPK pathway altered gastric cancer.

Key issues: The manuscript could be improved by providing more detail and clarity on the methods and results as detailed below:

1. What does three experiments mean, is it triplicate wells for each assay, or is it single well for each assay, but 3 biological replicates done on different passage? For the viability plot presented in figure 3-5 with or without error bar, are they based on viability assay from triplicate wells of one experiment, or an average of three biological replicates but one well per experiment?

We agree with the reviewer that the description of the viability analyses needs clarification. We tested each organoid line at three different passages independent from each other. Within each experiment, two identically treated wells were used for each drug concentration and the untreated control. All technical replicates (3 passages x 2 wells = 6 wells) were averaged and the standard deviation calculated resulting in one value +/- SD per experiment. We changed the description accordingly in the “Material and Methods” section within the ‘Drug library screening and viability measurement` paragraph.

2. Table EV4. How are the molecular background of the human gastric cancer organoids determined? The criteria to classify the RTK/MAPK altered versus non-altered are not clear. Is the green text denoting those that were considered altered? Should there be difference between gain and amplification? How is oe (?over-expression) detected, is it by transcriptome or other means? If by transcriptome, what is the cut-off? The un-altered group also has HRAS CNV gain (e.g. DD191_2). How to distinguish path (green color text) from ?non-pathogenic mutation. Are all mutation confirmed to be somatic (i.e. subtracted from paired normal)? The unaltered group seems to have some cases with RTK member with oe also.

Thank you for this comment, the information was indeed missing in the previous manuscript. Based on current knowledge from literature on the impact of alteration on this pathway, mutations in the EGFR family and also of downstream signaling mediators such as RAS, RAF and other MAP-kinases that can lead to an activation of MAPK signaling were taken into account. Pathogenic mutations were identified using the cosmic data base and given FATHMM (Functional Analysis Through Hidden Markov Models) prediction, with scores above 0.90 being considered pathogenic. In addition, EGFR or ERBB2 overexpressing organoids (>3x fold change compared to normal organoids) were considered as RTK/MAPK altered. We now clarified the missing information in the “Expanded View Materials and Methods” section. Nevertheless, the selection of criteria remains indeed subjective and it can be discussed, if additional or different mutations should be considered. However, our selection of RTK/MAPK pathway altered PDOs seems to be confirmed by their higher sensitivity to downstream intervention via trametinib (Fig6 D).

3. Two worksheets were provided for table EV3. The column heading of Normal, RAS activated, Diffuse and WNT activated for the two worksheets do not have same sequence of arrangement and there seems to be a frameshift in the data. The log10IC50 provided in the table also do not seem to be on the same scale as those used in Fig EV4. For example, for the drug Belinostat, the Log10IC50 for the ras-activated group is 2.61 in the first worksheet of

Table EV3, 3.2 in the 2nd worksheet of Table EV3. Based on figure EV4B, the dose for IC 50 is between 0.1 to 1 nM (it is not sure whether the log10IC50 is based on nM or microM as Figure EV4B has some drug plotted in nM and some in uM scale); which in log10 uM range should be between -3 to -4. However, the number provided in table EV3 seems to be in a different scale.

Thank you very much for detecting this incongruity. Indeed, there was a shift in the column order. We now corrected Table EV3. The unit for the log10IC50 transformation was nM for all drugs. Indeed, not all drugs were scaled to nM. In order to avoid high numbers some were scaled to µM for clearer depiction. To be consistent, we now changed all values to nM.

Other specific comments: The supplementary tables should provide table title and legend for clarity.

We now added a title as well as a legend to each table and explained all abbreviations.

The authors presented results for Trametinib in Fig 5 (for human gastric cancer organoids), but not in Fig. 4. It would be better to show similar drug response result in Fig. 4 as well to see if they are concordant.

The murine organoids were screened for trametinib response using pre-printed drug plates, while the trametinib treatment in the human PDOs were performed individually and with a different concentration range (please see also the “Drug library screening and viability measurement” section in Material and Methods). Thus, the different organoid types, test setups and different concentration ranges for trametinib make a direct comparison difficult. Nevertheless, we now depict the trametinib response curve of murine organoids in Fig EV3.

Fig. 5G. The authors presented aggregated data for each HDACi for statistical comparison between the two groups, and showed a significant difference, but the difference for the individual HDACi does not reach statistical significance (Fig. EV5B). Thus the trend seems to be weak.

Indeed, correlating the responsiveness between trametinib and single HDAC inhibitors resulted only in a trend. Nevertheless, drug treatments revealed for some PDOs variations in individual sensitivities to different HDAC inhibitors. Thus, we could document for some PDOs a profound heterogeneity in terms of response within the drug class of HDAC inhibitors. This weakened the single drug correlations. Thus, in order to overcome individual comparisons and obtain a general picture, we aggregated the results of single HDAC inhibitor treatments. We think this analysis is appropriate to underline the overall correlation.

The manuscript could be improved by better integration of the proteomic part with the drug

screening data, e.g. is there any relationship between discoveries from the proteomic profiling with the drug response data?

Thanks for this comment. The proteomic data was obtained in organoids without treatment. We therefore do not have data that allows to analyze the effect of certain treatments on changes in the proteome. The integration of the proteomic data with the drug screening data is therefore difficult, but we agree that further investigations using our setup might result in a better understanding of the observed drug effects. However, re-visiting the proteomic data we were able to observe some trends that might be relevant in explaining one of the main findings of our manuscripts: the significant sensitivity of the RAS activated organoids towards HDAC inhibition (Fig 4 and Fig EV3). The GSEA analysis of the RAS activated organoids resulted in a trend of downregulation, although not significant, of the biological processes “chromatin organization” (NES -1.44, q.value=0.214), “chromatin remodeling” (NES -1.37, q.value=0.259), “ATP dependent chromatin remodeling” (NES -1.59, q.value=0.133), “histone deacetylation” (NES -1.18, q.value=0.434) and “regulation of histone modification” (NES -1.18, q.value=0.434) (Table EV2). It thus seems that the introduced mutations in the RAS activated organoid line have an impact on chromatin structure and modification. The effect was not seen in the other two models and could explain the sensitivity of the RAS activated organoids towards HDAC inhibition. However, further studies would be necessary to prove this hypothesis. We now discuss this data in the “Results” section on page 7 and in the “Discussion” on page 12-13.

Comments from Reviewer 3:

Referee #3 (Remarks for Author): Gastric cancer remains a difficult cancer to treat. Molecular (genomic) characterization efforts led by the TCGA have not yet translated to clinically meaningful improvements in diagnosis or treatment. In this report by Seidlitz et al., they expand their previous efforts at molecularly recapitulating the human molecular subtypes of gastric cancer with the generation of a WNT activated mouse organoid model. The authors then perform proteomic analysis and a 196 drug/compound screen to elucidate further molecular signatures and treatment sensitivities. Findings from these in vitro murine models were explored in human PDOs resulting in the key new finding that gastric cancers with RTK/MAPK pathway activation may be susceptible to HDAC inhibitors.

Major Comments:

1. The authors mention molecular classification based on TCGA studies. They should include

mention of the other molecular classification studies including the Singapore-Duke and ACRG studies (PMID 23684942 and 25894828).

We agree with the reviewer and include now the ACRG and the Singapore-Duke classification systems to the "Introduction" section of the paper (page 3).

It would also benefit the authors to make mention (Introduction or Discussion) that these detailed gastric cancer molecular studies have not translated to new improved GC diagnoses or treatments. In addition, these (TCGA) molecular classifications have had limited clinical applicability and are not readily used for any clinical decision making.

This is indeed an important points and we mention this fact now in the "Introduction" after describing the different molecular gastric cancer classification systems (page 3).

2. For the current manuscript, the authors use certain alleles to generate three subtypes. However, in their previous publication (PMID 29703791), Seidlitz et al., used slightly different alleles to generate the corresponding in vivo mouse models. Why have the authors chosen to use differing alleles for these murine organoid models? For example, previous CIN mouse model had KRAS, TP53, and SMAD4 alleles; and current model adopts only KRAS and TP53 alleles. And previous GS models used CDH1, APC, and KRAS alleles and CDH1, KRAS, and SMAD4 alleles; and current model adopts only the CDH1 and APC alleles. Was this done for simplicity or are there fundamental differences between these 3 allele and 2 allele models?

Indeed, we used the two allele models in order to have only one mitogenic signaling pathway activated per model. In the Seidlitz *et al.* 2019 publication, we aimed to model *in vitro* full blown gastric cancer and therefore combined three alleles that closely mimicked gastric cancer subtypes. Of course, drug screening and detailed proteome analyses of these models would also generate very interesting data sets, especially concerning the interaction of several oncogenic pathways. The aim of the current manuscript, as the reviewer correctly assumed, was more to analyze in not too complex but still relevant models the impact of individual mitogenic pathway activations on the gastric epithelium.

In the human disease, mutations are accumulated gradually over many years. How does concomitant activation/inactivation of alleles recapitulate the molecular pathogenesis seen in the human disease?

True, the mutations in the used model systems are activated at once. We nevertheless did not intend to model the molecular pathogenesis of gastric cancer over time (also long term cultures of the models would definitely be of interest), but rather to analyze the direct impact of mitogenic signaling pathway activation of the gastric epithelium and its impact to drug

response. More complex models with individual alterations patterns (as a result of *in vitro* accumulation of additional mutations) would make correlative analyses to drug responses difficult.

3. One of the main strengths of this manuscript is the generation of the third molecular GC model. The authors mention that for the MSI TCGA subtype 78% of cases have WNT pathway alterations and 77% of cases have TP53 alterations. Can the authors provide a reference for these numbers?

The number of WNT and TP53 pathway alterations in the MSI subtype is based on our own analyses of publicly available data from the TCGA study, similarly as we did in the Seidlitz *et al*. 2019 publication for the GS and CIN subtype. We now added the original study as reference (page 4).

It is inferred by the reviewer that the authors despite naming the model WNT activated are modeling the MSI TCGA subtype. If this is the case, then why have the authors not considered deleting MMR proteins as an allelic combination?

Indeed, the combination of WNT and TP53 alterations is very frequent in MSI gastric cancer. And indeed, modeling MSI gastric cancer would necessitate of course also alterations in one of the MMR genes. As this model would be genetically unstable (at least we assume this), it would genetically change over time and thus be not a very good “model”, as it would not have a fixed genetic landscape and results using this “model” over time could not be compared to each other. Furthermore, distributing this model would result in different results from different groups, which would cause confusion in the scientific community. Thus, we opted not to delete an MMR gene, and in line with this also not call the model “MSI model”, but rather name it according to the mitogenic pathway that we altered.

In addition, anti PDL-1 agents have become the backbone of treatment for MSI-high tumors (agnostic of tissue of origin) based on PMID 28596308. Assessing this would require an in vivo model (with intact tumor microenvironment) which is beyond the scope of this study. But the authors should at least comment and discuss this as a future direction of investigation.

We completely agree in principle with the reviewer concerning MSI gastric cancer treatment. As outline above, we did on purpose not delete an MMR gene to prevent a high mutational burden in our model. Thus, immunotherapy will in our view be unlikely to have an effect on the WNT activated model, even when tested in complex organoids model systems incl. immune cells. In our view, a promising system to test immunotherapy would be a mouse model in which MMR gene deletion is achieved via the Anxa10-CreER^{T2} recombinase.

4. In Fig EV1 C and D for the Tp53R172H/+ lanes, why is there only one band in the infected lanes for the RAS activated and WNT activated models? Given that this is a heterozygous allele, shouldn't 2 bands be seen similar to the KRAS G12D/+ experiments?

Indeed, the reviewer is right: due to heterozygosity of *Tp53* one would expect to find a second band (wild-type) after induction. However, it is known that mutations in one allele of the Tp53 gene are often followed by loss of the remaining wild-type *Tp53* allele (= loss of heterozygosity (LOH)). Several publications have described this phenomenon in mice with the $Tp53^{R172H}$. For example, Shetzer and colleagues published in 2014 a LOH in mesenchymal stem cells derived from mice heterozygous for the $Tp53^{R172H}$. The authors observed at day 12 (passage 7) after taking cells into culture a loss of the WT *Tp53* allele. Interestingly, only those stem cells with *Tp53* LOH later induced malignant tumors in mice (Shetzer et al., 2014, Cell death and differentiation, PMID: 24832469).

Jackson *et al*. have made similar observations in murine advanced lung adenocarcinoma. After application of Adeno-Cre and recombination of the conditional alleles. The authors analyzed the effect of the *Tp53* hotspot mutations R270H as well as R172H in combination with $Kras^{G12D}$ on lung tumor development. Interestingly, they observed a loss of the WT allele for both hotspot mutations in the tumors (Jackson et al 2005, Cancer Research, PMID: 16288016). A recent review nicely summarized the *TP53* LOH phenomenon in human gastric cancer patients (Blanchet et al., 2021, Cancers). We therefore interpret the observed data in our genotyping PCRs as LOH of *Tp53*, resulting in a single band in the genotyping PCR. We thank the reviewer to pinpoint this phenomenon also in our model. We added this information to the legend of EV Fig 1C to emphasize the phenomenon.

5. The authors only briefly characterize the organoid models in Figure 1A. They should consider moving some/all the data from Fig EV2 to Fig 1A as these are important stains that characterize and differentiate the various organoid models.

We agree with the reviewer and have now moved the data from Fig EV2 to Figure 1.

6. To clarify, the results obtained in EV2 were performed on organoids after selection and grown in no EGF (for the RAS activated model), and no WNT/Rspondin (for the Diffuse and WNT activated models).

Exactly. After Adeno Cre infection, the organoids were selected based on their mutations. For the RAS activated model, EGF was omitted from the medium. For the diffuse and WNT activated model, WNT/Rspondin was left out of the medium. After successful activation of the mutations validated by PCR, organoid models were further cultured in the appropriate

selection medium for all performed experiments. We have made this clear now in the manuscript in the “Material and Methods” section (page 14).

7. At the end of the first page of the Results section, the authors mention that the WNT activated model has a "rather small organoid size", however in Fig EV2 C the WNT activated model H&E looks larger compared to the WT. Perhaps chose a more representative picture.
The reviewer is completely right. We changed the HE stainings of the WNT activated model with more representative ones (Fig. 1).

8. Can the authors comment on why the diffuse model had the least number of proliferating cells? Could this be due to culturing these less adherent cells in ECM/Matrigel? Would these cells grow better/proliferate in non adherent growth conditions?
Indeed, the number of proliferating cells is less at a certain time in the diffuse model organoids. Surprisingly, the need to be splitted was the same in ratio and frequency compared to the other models. This could indicate that fewer proliferating cells potentially proliferate faster than in the other models, so the overall growth speed of the culture is the same. This hypothesis however would need further experiments for confirmation.

9. Proteomic analysis of these different organoid subtypes is a unique approach performed by the authors. Are the authors able to show "positive controls" or expected increased proteomic pathways such as RAS pathway for the RAS activated model and WNT regulation for the WNT activated model?
This is a relevant comment. Pathway analyses did not reveal a general upregulation of the respective GO terms. We have re-analyzed our data and have now included some “positive control” observations in the results section. For example, the diffuse organoids (*Cdh1 fl/fl*, *Apc fl/fl*) showed a downregulated expression of CDH1. The WNT activated organoids revealed an upregulation of the WNT target gene Mmp7, which is transcriptionally regulated by β-catenin. We have included these observations in the “Results” section (page 5).

In addition, how do the proteomic profiles of these genetically engineered murine organoid models compare to the human subtypes (ie PMID 29520031). Further comment on this point would be warranted in the discussion section.
We need to apologize for not discussing the paper from the Qin lab (Ge *et al*. 2018). The group described three different subtypes of diffuse gastric cancer based on proteomic analyses of tumor and nearby tissues: (1) a cell cycle enriched, (2) an EMT enriched and (3) an immune response enriched subtype. Interestingly, the immune response subtype showed next to upregulated immunological processes also an enrichment of adhesion pathways. In

line with this, we also observed within our diffuse organoid model an upregulation of adherens junction assembly with significantly increased expression of proteins such as PODXL, THBS1 and MUC18/MCAM. This data suggest a possible compensation mechanism induced by the loss of CDH1 function via upregulation of various proteins and pathways involved in cell adhesion. We discuss this correlation to human data in the "Discussion" on page 10-11.

10. For the diffuse subtype, why would these organoids display increased adherent junction assembly as a proteomic signature with loss of CDH1? They also look very loosely adherent based on the images the authors show.

Indeed, one would rather expect the opposite due to the loss of *Cdh1*. We can only hypothesize why we observed in our proteomic dataset a significantly increased adherent junction assembly. As outlined above (comment to 9), we found in addition to an overall activation of the pathway a significant upregulation of the cell adhesion molecule podocalyxin (PODXL). PODXL is a cell surface sialomucin of the CD34 family that is normally expressed in kidneys, vascular endothelial cells, hematopoietic progenitor cells, and mesothelia. The protein plays an important role in regulating cell adhesion and cell morphology. PODXL has been reported to be overexpressed in many epithelial carcinomas. There is evidence that overexpression of PODXL is associated with poor prognosis, aggressive tumor growth, and unfavorable treatment outcomes. We also observed significant upregulation of the cell adhesion molecule thrombospondin-1 (THBS1) and the cell surface glycoprotein MUC18 (MCAM). In contrast, downregulation of the desmosome proteins desmoglein-2 (DSG2) and desmocollin-2 (DSC2), as well as the integrins ITGA7 and ITGA1 was observed.

Thus, the loss of CDH1 seems to disarrange the adherens signaling pathways with an overall upregulation of proteins involved in its assembly. We hypothesize, as also already outlined above (comment to 9), that the altered expression of cell-cell adhesion proteins, particularly the increase in adherens junction assembly proteins, compensates for the loss of CDH1. Similar observations were also described by Chen et al. in 2014. The authors induced CDH1 loss in the breast cancer cell line MCF10A using zinc finger nuclease technology. They observed a similar altered adherens junction assembly phenotype in their model (PMID: 25079037). We included the above mentioned points in the "Results" (page 6) and "Discussion" part (pages 10-11).

11. The authors should be commended for performing a large drug library screen using their organoid models. They document interesting disparate sensitivities of each line to chemotherapy and targeted therapies. Again, this data begs the question on how the

sensitivity profiles of these murine organoid models compare to the human subtypes (ie PMID 28747339).

This is an interesting suggestion. Nevertheless, when trying to investigate this, it becomes apparent that the chemotherapy in large published datasets is heterogenous. This is also the case in the study referred to by the reviewer. The administered adjuvant chemotherapy in the studied patient population was “standard adjuvant chemotherapy [either single-agent 5-fluorouracil (5-FU) or a combination of 5-FU and cisplatin/oxaliplatin, doxorubicin, or paclitaxel]”. Thus, even if a good and bad response to adjuvant chemotherapy could be revealed for the CIN and GS subtype, respectively, it is unclear to which chemotherapy precisely this is true. And response varies in our models from drug to drug for the different models (Fig. 4 and EV3).

In addition, can the authors comment on how these particular targeted pathways were selected and other pathways such as VEGF (with known drugs approved for GC such as ramucirumab not assessed)?

The initial medium-scale drug library test was composed of small molecule inhibitors targeting various pathways. In the chosen test setup antibodies such as ramucirumab and trastuzumab were not tested as their use in pre-printed drug plates was technically not optimized yet. We nevertheless also tested small molecules inhibiting VEGFR (i.e.: pazopanib, sunitinib, lenvatinib, tivozanib, dovitinib, linsitinib). Although covering a wide range of concentrations, these anti-angiogenic agents only had minor effects on the organoid viability. This is potentially due to the missing micromilieu incl. vasculature in the organoids used in this study (purely consisting of epithelial cells). In the future, more advanced organoid culture systems including endothelial cells would possible be more appropriate to test anti-angiogenic drugs.

12. The authors found that PI3K/mTOR inhibitors did not result in differential responses between the models and normal organoids. Based on TCGA data, this pathway was more selectively mutated for the EBV and MSI subgroups, 80 and 42% respectively. Did the authors find proteomic evidence of PI3K/mTOR pathway activation?

Indeed, we could not see a differential response between the different organoids models to PI3K/mTor inhibitors. We re-analyzed our generated proteomic data of the three models regarding PI3K/mTOR pathway activation. In the Rebuttal Table 1 (below) proteins related to the PI3K/mTOR pathway (chosen from Panther classification system) are listed per model with their corresponding Log2 values. No striking pattern which would indicate a significantly activation in any of the three models could be overserved. In line with this, no enrichment of the whole pathway was found in the GSEA analysis (Table EV1).

Rebuttal Table 1

Gene name	RAS activated / Normal	(-)Log10 pValue_RAS activated	Significant RAS activated vs. Normal	Diffuse / Normal	(-)Log10 pValue_Diffuse	Significant Diffuse vs. Normal	WNT activated / Normal	(-)Log pValue_WNT activated	Significant WNT activated vs. Normal
Gnai1	NaN	NaN		0,107	0,181		-0,023	0,072	
Gna14	0,071	0,086		NaN	NaN		-0,022	0,077	
Insr	-0,414	0,884		NaN	NaN		-0,130	0,513	
Ccnd2	NaN	NaN		0,484	0,469		-0,247	0,782	
Grb2	-0,102	0,237		0,024	0,035		-0,172	1,151	
Hras	0,720	1,092		NaN	NaN		-0,229	0,444	
Rps6kb1	NaN	NaN		NaN	NaN		0,016	0,041	
Pten	-0,410	0,902		NaN	NaN		-0,092	0,195	
Gnb2	0,466	1,004		-0,134	0,205		0,105	0,256	
Pik3r1	0,153	0,295		NaN	NaN		0,055	0,285	
Inppl1	-0,016	0,024		-0,168	0,311		0,026	0,094	
Pdpk1,Pdk1	0,018	0,029		-0,415	0,971		-0,104	0,533	
Inpp5a	0,581	1,254		-0,145	0,340		-0,107	0,786	
Foxo1	NaN	NaN		NaN	NaN		0,195	0,558	
Gnai3	0,299	0,605		0,063	0,126		-0,009	0,038	
Casp9	-0,845	2,149	DOWN	-0,230	0,558		0,143	0,418	
Foxo3	-0,033	0,042		NaN	NaN		0,325	0,707	
Sos1	-0,145	0,198		-0,196	0,171		0,217	0,703	
Gnb1	0,304	0,512		-0,067	0,111		-0,009	0,026	
Pik3ca	NaN	NaN		NaN	NaN		-0,123	0,732	
Ccnd1	-0,653	0,814		0,416	0,807		-0,274	1,223	
Kras	0,717	1,834	UP	-0,210	0,495		0,099	0,816	
Akt3	-0,191	0,288		NaN	NaN		NaN	NaN	
Gnaq	0,443	1,191		0,024	0,050		-0,002	0,010	
Gsk3b	-0,231	0,406		-0,014	0,028		-0,177	1,059	
Gnai2	0,466	0,937		0,108	0,245		-0,034	0,252	
Nras	0,357	0,942		-0,605	1,366	DOWN	0,004	0,023	
Pik3cb	NaN	NaN		0,114	0,144		-0,090	0,243	
Gna11	0,227	0,546		-0,393	1,396	DOWN	-0,052	0,284	
Akt1	-0,330	0,630		0,269	0,493		0,084	0,806	
Jak2	NaN	NaN		NaN	NaN		0,054	0,090	
Ywhaz	-0,321	0,458		0,260	0,311		0,047	0,224	
Akt2	NaN	NaN		NaN	NaN		0,247	2,126	
Irs1	NaN	NaN		NaN	NaN		-0,125	0,380	

13. The authors demonstrate variable response of the murine organoids to EGFR/MAPK pathway inhibition. The reviewer believes that demonstrating the activity of this pathway by immunoblot assay or IHC would strengthen the data and possibly lead to explanations as to the variable responses seen. Extension of these studies could also be considered for the human PDO data again to explain the difficulty in predicting a response based on the presence of certain pathway alterations.

Based on the reviewer's comment, we performed Western blot after inhibition of MAPK signaling pathway in the three murine organoid models. We specifically analyzed the phosphorylation status of downstream ERK1/2 after inhibition of MEK1/2 by trametinib. We

choose trametinib because a differential responses of the organoid lines to the drug was documented (Fig 4C, the RAS activated organoid line was more sensitive compared to the diffuse or WNT activated organoid lines) and we used this drug also for the characterization of the human PDOs.

After inhibition with 10µM trametinib for 60min, we harvested the organoids, isolated protein and performed Western blot (please see Rebuttal Fig. 1 below).

(A) We analyzed ERK1/2, pERK1/2 and as a housekeeping protein GAPDH in the RAS activated, diffuse and WNT activated organoid models. Untreated (DMSO) control organoids of all models showed a strong phosphorylation status of ERK1/2. The pathway is therefore strongly active in all models, also only the RAS activated model has a mutated *Kras*. The activated MAPK signaling pathway in all models might be explained by the supplementation of EGF in the growth medium. In all three lines, a complete loss of the ERK1/2 phosphorylation after trametinib inhibition was observed (Rebuttal Fig. 1A).

(B) We also tested normal organoids and observed a similar pattern. The untreated (DMSO) control organoids showed active MAPK signaling. Normal organoids were also cultivated with EGF in the growth medium. Upon treatment with trametinib, ERK1/2 is completely dephosphorylated after 60min (Rebuttal Fig. 1B).

(C) We then raised the question whether the *Kras* mutation vs. the concomitant supplementation of EGF in the medium of the diffuse and WNT activated organoids during trametinib treatment could explain the differential response. We therefore cultured the diffuse and WNT activated organoids for one week before trametinib treatment in medium without EGF and subsequently analyzed the pERK1/2 level in untreated (DMSO) control and treated organoids. Interestingly, both models even without EGF supplementation showed active MAPK signaling as demonstrated by the phosphorylation level of ERK1/2 (Rebuttal Fig. 1C). We again observed a loss of pERK1/2 in treated organoids. Cultivation of the lines in the medium without EGF for a longer time period than 1 week was not possible, as they did not grow adequately without the supplementation.

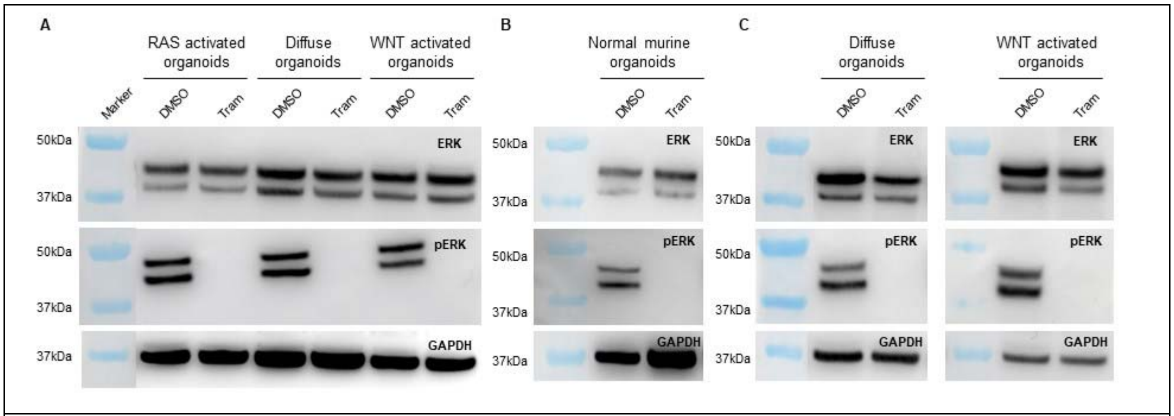

Rebuttal Fig. 1

In summary, all three models show an active MAPK signaling. The signaling in the non-*Kras* mutated models seems to be not only dependent on the EGF supplementation in the medium, as active signaling was still observed after 1 week on absence of EGF. This hints to an activation of the MAPK pathway through other mechanisms, potentially through the activation of the WNT pathway in both other models. Nevertheless, the activation via other mechanism is not sufficient to maintain long term growth of the organoids. All three models show an inhibitor specific loss pERK1/2 after trametinib and thus a loss of MAPK signaling. We could therefore not uncover any rationale in the differential response of the organoids using the performed analyses of the activation status of the MAPK pathway via Western blot. For this reason, we chose not to include the generated data in the manuscript, as the underlying mechanism is unclear. It nevertheless indeed is an interesting question to answer in the future with more extensive analyses.

14. The authors next generate a number of human GC PDOs. What are the demographics for these patients (ie sex, stage, age) and how specifically were the samples obtained via biopsy or surgical resection? In addition, some correlation between PDO and primary tumor should be shown as was demonstrated in the authors previous publication.

Valid point, we now summarized the demographics and added this information in the Table EV4 “PDO information/patient information”. As suggested by the reviewer, we extended the comparative histological analysis (HE and PAS) between the PDO and the primary tumor to five more primary tumor / corresponding PDO couples. As previously demonstrated by us (Seidlitz et al 2018, Gut) and others, similarities between primary tumors and PDOs could be demonstrated (Fig. EV4). For example, mixed morphologies of intestinal and diffuse growing patterns in the primary tissue of DD483 can also be seen in the organoid culture of DD483, which contains organoids with cystic shape typical for intestinal growing tumors but also poorly adhering single cells characteristic for diffuse growing tumors. We added these findings to the “Results” section on page 8.

Using these PDOs, the authors assess for RTK/MAPK pathway alterations, would it be possible for the authors to do a more expansive genomic mutational assessment and actually attempt to subgroup these PDOs into the TCGA subtypes.

The main purpose of analyzing the PDOs was to demonstrate an association between MAPK pathway activation and HDAC sensitivity also in human cancer. To this end, a classification was needed concerning the MAPK signaling pathway (Table EV4). A further subgrouping into the TCGA subtypes is in our view not expedient.

15. In the discussion, the authors state that targeted therapies are thought to at least accompany, if not replace classical chemotherapy for GC treatment. Can they provide citations? No single agent targeted therapies have been approved for GC in the frontline setting PMID 28736629.

We wanted to strengthen the importance of functionally testing targeted therapies in organoids. But we actually completely agree with the reviewer, targeted therapies have so far not and are unlikely in the future to completely replace (with the potential exception of immunotherapy in MSI gastric cancer) classical chemotherapy. We have therefore changed by removing "if not replace" the sentence in the "Discussion" (page 11).

In addition, the authors state that therapeutic targeting of the ERBB pathway represents a promising goal for GC. HER2 targeting agents in combination with chemotherapy is standard of care now for metastatic GC based on the ToGA study that the authors already reference.

Indeed, the sentence was not clearly written. We now rephrased it and point out that the ERBB signaling pathway is already a promising therapeutic target in HER2 positive gastric carcinoma (page 12).

16. The authors demonstrate that the PDOs have different sensitivities to MEK1/2 inhibition in Fig5D. What about EGFR and BRAF inhibition?

This comment resulted in interesting additional data. We performed additional drug assays using the same PDOs for a BRAF inhibitor (LY3009120) and a pan-EGFR inhibitor (afatinib). We observed different sensitivities between the PDO lines and similar to trametinib highest sensitivity was observed in normal gastric PDOs. In contrast to trametinib, no significant difference between RTK/MAPK altered and unaltered PDOs could be observed for afatinib ($p=0.7588$) and LY3009120 ($p=0.2612$). Data was added to Fig EV5 and described on page 9. Corresponding AUC and standard deviations were added in the Table EV5 and Table EV6. Thus, treatment of the whole cohort of MAPK activated gastric cancer was successful only on the level of MEK1/2, but not higher up in the signaling cascade.

17. Can the authors comment on a particular mechanism or hypothesize about a mechanism as to why RTK/MAPK activated GCs have sensitivity to HDAC inhibition? Would this be a generalizable mechanism for other cancers?

We cannot answer this question currently based on own data, but completely agree with the reviewer that it will be very interesting to address this in the future. As it is a very relevant question which other readers possibly might also be interested in, we added a hypothesis to the discussion part (pages 12-13). As mitogenic signals drive the cell cycle into S phase and cells into proliferation, they are normally under control of a variety of check points mainly regulated by tumor suppressor proteins. Strong activation of mitogenic pathways such as MAPK pathway is frequently observed in a variety of cancer types. In order to escape cell cycle control cancer cells can undergo a variety of changes. One of this mechanism is known to be epigenetic silencing. HDACs mediate such gene silencing via reducing DNA accessibility. As deacetylated histones bind DNA stronger, transcriptional regulators cannot act and gene expression is silenced. The inhibition of HDACs blocks this silencing process, so chromatin can open up and silenced genes can be transcribed. Cancer cells that relied on HDAC mediated gene silencing could face an activation of tumor suppressors and would therefore be likely to enter cell cycle arrest or apoptosis. As we have seen in this study, that RTK/MAPK altered PDOs tend to be more sensitive to HDAC inhibition, we hypothesize that HDAC mediated gene silencing might be one of pivotal adaption processes in MAPK activated gastric cancer. This adaption process can be reset by HDAC inhibitors. Future research looking into chromatin remodeling could reveal if this hypothesis is true or false.

Minor Comments:

1. The syntax of the sentence at the end of the first paragraph of the Introduction starting "Due to their highly individual pattern..." is difficult to understand. The authors should consider rewriting.

We have rephrased the sentence and hope it is now more understandable (pages 3-4): “Each PDO line has an individual pattern of molecular alterations with hundreds of mutations and deregulated signaling pathways. Due to this, they represent unique avatars of a patient, rather than models for a particular cancer (subtype).”

2. In the second paragraph of results, the authors should clarify that the Normal gastric corpus organoids are Normal Murine gastric corpus organoids.

We have now clarified this (page 4).

3. In the sentence "The CDH1 loss in the diffuse organoid model...", "let as reported" is an unclear phrase.

We have rephrased the sentence. It now reads as follows: “The *Cdh1* loss in the diffuse organoid model let to a complete change of organoid morphology towards a grape like structure” (pages 4-5).

4. For Fig EV3B, would please keep the location and position of the stars consistent and ideally above the bar graphs.

Thank you for pointing this out. We have now positioned the significance stars in a consistent manner in the center over the graphs.

Would also define PI for the axis in A and apcp in B.

We apologize for the mistake. Apcp means apoptotic cells. PI is propidium iodide and was analyzed in the PE/Texas Red channel during FACS analysis. We have corrected this in the figure.

Also, the reviewer may have missed this but no mention of the methodology for the cell cycle assay was mentioned in the M&M section.

The methodology for the cell cycle assay can be found in the Expanded View Material and Methods section.

5. Proteomic GSEA analysis methodology should also be mentioned and detailed in the M&M section.

Detailed information on the methodology can be found in the Expanded View Material and Methods section.

6. Page 2 of the results last sentence starting "The elevated RTK/RAS..." "let to translational activation" is an unclear phrase.

We rephrased the sentence to avoid ambiguity: “An activation of translational processes was detected in the RAS activated organoids, …” (page 6).

7. Page 5 of the results, sentence starting "within the cancer PDOS, a significantly higher sensitivity of PDOS with altered vs unaltered RTK/MAPK pathway could be observed, it is unclear sensitivity to what drug?

The reviewer is right. We added the missing information to the sentence: “Within the cancer PDOs, a significantly higher sensitivity of PDOs with altered vs. unaltered RTK/MAPK pathway towards MEK1/2 inhibition by trametinib could be observed.” (page 9).

8. Fig EV5B, what does the "normal" and the dotted line for this graph represent?
“Normal” represents the value of the normal gastric PDOs as a reference parameter. We have explained this now in the figure legend.

9. Fig 5 E, please indicated what the green and black X axis coloring represent.
In Fig 5 E, the colors correspond to the different murine models as indicated by the legend at the top of the figure. In Fig 6E, the color-coding corresponds to the RTK/MAPK status: altered (green), unaltered (black) and normal (orange) as indicated by the legend below panel B-D. We have added a description of the color code to the figure legend. The color code is now also mentioned in the text of the “Results” section (page 9).

10. Page 2 of the Discussion, the phrase "Even so classical chemotherapy..." is unclear.
See answer to point 15 further up. We have rephrased the sentence to: “Classic chemotherapy is still the backbone of gastric cancer treatment up to today. However, targeted therapies are expected to accompany them increasingly in the future.” (page 11).

1st Revision - Editorial Decision 25th Jul 2022

25th Jul 2022

Dear Dr. Stange,

Thank you for the submission of your revised manuscript to EMBO Molecular Medicine. We have now received the enclosed report from the two referees who were asked to re-assess it. As you will see, the referees are now supportive, and I am pleased to inform you that we will be able to accept your manuscript pending the following editorial-level amendments:

1. Please reduce the keyword number to five.

2. We updated our journal's competing interests policy in January 2022 and request authors to consider both actual and perceived competing interests. Please review the policy https://www.embopress.org/competing-interests and update your competing interests if necessary.
Please use the heading "Disclosure statement and competing interests".

3. Data availability: Please make sure the proteomics datasets will be made publicly accessible upon the acceptance of the manuscript.

4. Please rename "Grant Support" as "Acknowledgements".

5. Remove the list of "Abbreviations" from the main manuscript file.

6. CRediT has replaced the traditional author contributions section because it offers a systematic machine-readable author contributions format that allows for more effective research assessment. Please use the free text boxes beneath each contributing author's name to add specific details on the author's contribution. More information is available in our guide to authors. Remove the author contribution section from the manuscript file.

7. EV tables

- Tables EV1-3 are rather large. They should be updated to Datasets using the nomenclature Dataset EV1, Dataset EV2, and so on.
- Renumber the remaining EV tables.
- Update all corresponding callouts in the manuscript.

8. The "Expended View Material and Methods" should be merged with the "Materials and Methods" section in the main manuscript file. Please remove Abbreviation.

9. I have slightly modified the synopsis text (see attached). Please let me know if it is fine as is or if you would like to introduce further modifications.

10. Our data editors have seen the manuscript, and they have made some comments and suggestions that need answering (in track change mode, see attached). Please send back a track changes file as we will need to go through the changes.

11. As part of the EMBO Publications transparent editorial process initiative (see our Editorial at http://embomolmed.embopress.org/content/2/9/329), EMBO Molecular Medicine will publish online a Review Process File (RPF) to accompany accepted manuscripts.

In the event of acceptance, this file will be published in conjunction with your paper and will include the anonymous referee reports, your point-by-point response, and all pertinent correspondence relating to the manuscript. Let us know if you DISAGREE with this or if you want to remove or keep any figures from it prior to publication.

Please note that the Authors checklist will be published at the end of the RPF.

Please submit your revised manuscript within two weeks.

I look forward to reading a new revised version of your manuscript.

Kind regards,
Jingyi

Jingyi Hou
Editor
EMBO Molecular Medicine

*

***** Reviewer's comments *****

Referee #2 (Remarks for Author):

The revised version shows substantial improvement and has clarified most of the previous questions. We have no further comment.

Referee #3 (Comments on Novelty/Model System for Author):

The authors here generate 3 genetic murine GC organoid subtypes that mimic aspects of the human TCGA subtypes. They perform unique proteomic characterization and drug/compound screening of these organoids. Certain aspects of their findings are recapitulated in actual human PDOs. Key findings are differential RTK/MAPK pathway inhibition in the subtypes and a sensitivity of the RAS activated model to HDAC inhibition. These organoids models are technical and may represent certain key aspects of the human molecular subtypes. However, these organoid models lack a tumor microenvironment and none of these models were explored in vivo. Given the importance and controversies of immunotherapy/anti-PDL1 blockade in GC, this is still a major area that needs to be studied.

Referee #3 (Remarks for Author):

In this report by Seidlitz et al., they generate 3 genetic murine GC organoid subtypes that mimic aspects of the human TCGA subtypes. The authors then perform proteomic analysis and a 196 drug/compound screen to elucidate further molecular signatures and treatment sensitivities. Findings from these in vitro murine models were explored in human PDOs resulting in the key new finding that gastric cancers with RTK/MAPK pathway activation may be susceptible to HDAC inhibitors. The authors have eloquently and sufficiently addressed the previous Major and Minor comments raised by the reviewer. It will be interesting moving forward to see how/if approaches such as this will be translatable clinically. As the authors point out, they are using a simple oncogene driven approach here. Whereas, human gastric cancer (at least the CIN subtype) is no doubt more heterogeneous. The major philosophical question that remains is can a similar approach be implemented using only human PDOs? In other words, can we use "omics" and human gastric cancer PDOs to personalize cancer treatment and exploit new treatment sensitivities at an individual patient level? An a priori "basket" trial strategy in which predetermined targetable pathways is unlikely to be fruitful as shown by Ooft et al for colorectal cancer (PMID: 33887686). This study is important in of itself to show the utility of PDOs as a pre-clinical tool, but also the potential for HDACis as a novel treatment for GC.

The authors performed the requested changes.

2nd Aug 2022

Dear Dr. Stange,

We are pleased to inform you that your manuscript is accepted for publication and is now being sent to our publisher to be included in the next available issue of EMBO Molecular Medicine.

We would like to remind you that as part of the EMBO Publications transparent editorial process initiative, EMBO Molecular Medicine will publish a Review Process File online to accompany accepted manuscripts. If you do NOT want the file to be published or would like to exclude figures, please immediately inform the editorial office via e-mail.

Please read below for additional IMPORTANT information regarding your article, its publication and the production process.

Congratulations on your interesting work,

Jingyi

Jingyi Hou
Editor
EMBO Molecular Medicine

Follow us on Twitter @EmboMolMed
Sign up for eTOCs at embopress.org/alertsfeeds

EMBO Press Author Checklist

Corresponding Author Name: Daniel E. Stange

Journal Submitted to: EMBO Molecular Medicine
Manuscript Number: EMM 2022-15705

USEFUL LINKS FOR COMPLETING THIS FORM
The EMBO Journal - Author Guidelines
EMBO Reports - Author Guidelines
Molecular Systems Biology - Author Guidelines
EMBO Molecular Medicine - Author Guidelines

Reporting Checklist for Life Science Articles (updated January 2022)
This checklist is adapted from Materials Design Analysis Reporting (MDAR) Checklist for Authors. MDAR establishes a minimum set of requirements in transparent reporting in the life sciences (see Statement of Task: 10.31222/osf.io/9sm4x). Please follow the journal's guidelines in preparing your manuscript.
Please note that a copy of this checklist will be published alongside your article.

Abridged guidelines for figures
1. Data
The data shown in figures should satisfy the following conditions:
- the data were obtained and processed according to the field's best practice and are presented to reflect the results of the experiments in an accurate and unbiased manner.
- ideally, figure panels should include only measurements that are directly comparable to each other and obtained with the same assay.
- plots include clearly labeled error bars for independent experiments and sample sizes. Unless justified, error bars should not be shown for technical replicates.
- if n<5, the individual data points from each experiment should be plotted. Any statistical test employed should be justified.
- Source Data should be included to report the data underlying figures according to the guidelines set out in the authorship guidelines on Data Presentation.

2. Captions
Each figure caption should contain the following information, for each panel where they are relevant:
- a specification of the experimental system investigated (eg cell line, species name).
- the assay(s) and method(s) used to carry out the reported observations and measurements.
- an explicit mention of the biological and chemical entity(ies) that are being measured.
- an explicit mention of the biological and chemical entity(ies) that are altered/varied/perturbed in a controlled manner.
- the exact sample size (n) for each experimental group/condition, given as a number, not a range;
- a description of the sample collection allowing the reader to understand whether the samples represent technical or biological replicates (including how many animals, litters, cultures, etc.).
- a statement of how many times the experiment shown was independently replicated in the laboratory.
- definitions of statistical methods and measures:
  - common tests, such as t-test (please specify whether paired vs. unpaired), simple χ2 tests, Wilcoxon and Mann-Whitney tests, can be unambiguously identified by name only, but more complex techniques should be described in the methods section;
  - are tests one-sided or two-sided?
  - are there adjustments for multiple comparisons?
  - exact statistical test results, e.g., P values = x but not P values < x;
  - definition of 'center values' as median or average;
  - definition of error bars as s.d. or s.e.m.

Please complete ALL of the questions below.
Select "Not Applicable" only when the requested information is not relevant for your study.

Materials

Newly Created Materials	Information included in the manuscript?	In which section is the information available? (Reagents and Tools Table, Materials and Methods, Figures, Data Availability Section)
New materials and reagents need to be available; do any restrictions apply?	Not Applicable	

Antibodies	Information included in the manuscript?	In which section is the information available? (Reagents and Tools Table, Materials and Methods, Figures, Data Availability Section)
For **antibodies** provide the following information:<br>- Commercial antibodies: RRID (if possible) or supplier name, catalogue number and or/clone number<br>- Non-commercial: RRID or citation	Yes	Expanded View Material and Methods

DNA and RNA sequences	Information included in the manuscript?	In which section is the information available? (Reagents and Tools Table, Materials and Methods, Figures, Data Availability Section)
**Short novel DNA or RNA including primers, probes**: provide the sequences.	Yes	Expanded View Material and Methods

Cell materials	Information included in the manuscript?	In which section is the information available? (Reagents and Tools Table, Materials and Methods, Figures, Data Availability Section)
**Cell lines:** Provide species information, strain. Provide accession number in repository **OR** supplier name, catalog number, clone number, and/**OR** RRID.	Not Applicable	
**Primary cultures:** Provide species, strain, sex of origin, genetic modification status.	Yes	Material and Methods, Table EV4
Report if the cell lines were recently **authenticated** (e.g., by STR profiling) and tested for mycoplasma contamination.	Not Applicable	

Experimental animals	Information included in the manuscript?	In which section is the information available? (Reagents and Tools Table, Materials and Methods, Figures, Data Availability Section)
**Laboratory animals or Model organisms:** Provide species, strain, sex, age, genetic modification status. Provide accession number in repository **OR** supplier name, catalog number, clone number, **OR** RRID.	Not Applicable	
**Animal observed in or captured from the field:** Provide species, sex, and age where possible.	Not Applicable	
Please detail **housing and husbandry conditions**.	Not Applicable	

Plants and microbes	Information included in the manuscript?	In which section is the information available? (Reagents and Tools Table, Materials and Methods, Figures, Data Availability Section)
**Plants:** provide species and strain, ecotype and cultivar where relevant, unique accession number if available, and source (including location for collected wild specimens).	Not Applicable	
**Microbes:** provide species and strain, unique accession number if available, and source.	Not Applicable	

Human research participants	Information included in the manuscript?	In which section is the information available? (Reagents and Tools Table, Materials and Methods, Figures, Data Availability Section)
If collected and within the bounds of privacy constraints report on age, sex and gender or ethnicity for all study participants.	Yes	Table EV4

Core facilities	Information included in the manuscript?	In which section is the information available? (Reagents and Tools Table, Materials and Methods, Figures, Data Availability Section)
If your work benefited from core facilities, was their service mentioned in the acknowledgments section?	Not Applicable	

Design

Study protocol	Information included in the manuscript?	In which section is the information available? (Reagents and Tools Table, Materials and Methods, Figures, Data Availability Section)
If study protocol has been **pre-registered, provide DOI in the manuscript**. For clinical trials, provide the trial registration number **OR** cite DOI.	Not Applicable	
Report the **clinical trial registration number** (at ClinicalTrials.gov or equivalent), where applicable.	Not Applicable	

Laboratory protocol	Information included in the manuscript?	In which section is the information available? (Reagents and Tools Table, Materials and Methods, Figures, Data Availability Section)
Provide DOI OR other citation details if **external detailed step-by-step protocols** are available.	Not Applicable	

Experimental study design and statistics	Information included in the manuscript?	In which section is the information available? (Reagents and Tools Table, Materials and Methods, Figures, Data Availability Section)
Include a statement about **sample size** estimate even if no statistical methods were used.	Yes	Material and Methods
Were any steps taken to minimize the effects of subjective bias when allocating animals/samples to treatment (e.g. **randomization procedure**)? If yes, have they been described?	Not Applicable	
Include a statement about **blinding** even if no blinding was done.	Not Applicable	
Describe **inclusion/exclusion criteria** if samples or animals were excluded from the analysis. Were the criteria pre-established?<br><br>If sample or data points were omitted from analysis, report if this was due to attrition or intentional exclusion and provide justification.	Not Applicable	
For every figure, are **statistical tests** justified as appropriate? Do the data meet the assumptions of the tests (e.g., normal distribution)? Describe any methods used to assess it. Is there an estimate of variation within each group of data? Is the variance similar between the groups that are being statistically compared?	Yes	Material and Methods

Sample definition and in-laboratory replication	Information included in the manuscript?	In which section is the information available? (Reagents and Tools Table, Materials and Methods, Figures, Data Availability Section)
In the figure legends: state number of times the experiment was **replicated** in laboratory.	Yes	Figure legends
In the figure legends: define whether data describe **technical or biological replicates**.	Yes	Figure legends

Ethics

Ethics	Information included in the manuscript?	In which section is the information available? (Reagents and Tools Table, Materials and Methods, Figures, Data Availability Section)
Studies involving **human participants**: State details of **authority granting ethics approval** (IRB or equivalent committee(s), provide reference number for approval.	Yes	Material and Methods
Studies involving **human participants**: Include a statement confirming that **informed consent** was obtained from all subjects and that the experiments conformed to the principles set out in the WMA Declaration of Helsinki and the Department of Health and Human Services Belmont Report.	Yes	Material and Methods
Studies involving **human participants:** For publication of **patient photos**, include a statement confirming that consent to publish was obtained.	Not Applicable	
Studies involving experimental **animals**: State details of **authority granting ethics approval** (IRB or equivalent committee(s), provide reference number for approval. Include a statement of compliance with ethical regulations.	Yes	Material and Methods
Studies involving **specimen and field samples:** State if relevant **permits** obtained, provide details of authority approving study; if none were required, explain why.	Not Applicable	

Dual Use Research of Concern (DURC)	Information included in the manuscript?	In which section is the information available? (Reagents and Tools Table, Materials and Methods, Figures, Data Availability Section)
Could your study fall under dual use research restrictions? Please check biosecurity documents and list of **select agents and toxins** (CDC): https://www.selectagents.gov/sat/list.htm	Not Applicable	
If you used a select agent, is the security level of the lab appropriate and reported in the manuscript?	Not Applicable	
If a study is subject to dual use research of concern regulations, is the name of the **authority granting approval and reference number** for the regulatory approval provided in the manuscript?	Not Applicable	

Reporting

The MDAR framework recommends adoption of discipline-specific guidelines, established and endorsed through community initiatives. Journals have their own policy about requiring specific guidelines and recommendations to complement MDAR.

Adherence to community standards	Information included in the manuscript?	In which section is the information available? (Reagents and Tools Table, Materials and Methods, Figures, Data Availability Section)
State if relevant guidelines or checklists (e.g., **ICMJE, MIBBI, ARRIVE, PRISMA**) have been followed or provided.	Yes	ICMJE
For **tumor marker prognostic studies,** we recommend that you follow the **REMARK** reporting guidelines (see link list at top right). See author guidelines, under 'Reporting Guidelines'. Please confirm you have followed these guidelines.	Not Applicable	
For **phase II and III randomized controlled trials**, please refer to the **CONSORT** flow diagram (see link list at top right) and submit the CONSORT checklist (see link list at top right) with your submission. See author guidelines, under 'Reporting Guidelines'. Please confirm you have submitted this list.	Not Applicable	

Data Availability

Data availability	Information included in the manuscript?	In which section is the information available? (Reagents and Tools Table, Materials and Methods, Figures, Data Availability Section)
Have **primary datasets** been deposited according to the journal's guidelines (see 'Data Deposition' section) and the respective accession numbers provided in the Data Availability Section?	Yes	Data availability section - mass spectrometry proteomics data
Were **human clinical and genomic datasets** deposited in a public access-controlled repository in accordance to ethical obligations to the patients and to the applicable consent agreement?	Not Applicable	
Are **computational models** that are central and integral to a study available without restrictions in a machine-readable form? Were the relevant accession numbers or links provided?	Not Applicable	
If publicly available data were reused, provide the respective **data citations in the reference list**.	Yes	References
